# Identification of the Transcription Factor Znc1p, which Regulates the Yeast-to-Hypha Transition in the Dimorphic Yeast *Yarrowia lipolytica*

**DOI:** 10.1371/journal.pone.0066790

**Published:** 2013-06-24

**Authors:** Azul Martinez-Vazquez, Angelica Gonzalez-Hernandez, Ángel Domínguez, Richard Rachubinski, Meritxell Riquelme, Patricia Cuellar-Mata, Juan Carlos Torres Guzman

**Affiliations:** 1 Departamento de Biologia, Division de Ciencias Naturales y Exactas, Universidad de Guanajuato, Campus Guanajuato, Guanajuato, Mexico; 2 Departamento de Microbiologia y Genetica, CIETUS/IBSAL, Universidad de Salamanca, Salamanca, Spain; 3 Department of Cell Biology, University of Alberta, Edmonton, Alberta, Canada; 4 Departamento de Microbiologia, Centro de Investigacion Cientifica y de Educacion Superior de Ensenada (CICESE), Ensenada, Baja California, Mexico; Université de Nice-CNRS, France

## Abstract

The dimorphic yeast *Yarrowia lipolytica* is used as a model to study fungal differentiation because it grows as yeast-like cells or forms hyphal cells in response to changes in environmental conditions. Here, we report the isolation and characterization of a gene, *ZNC1*, involved in the dimorphic transition in *Y. lipolytica*. The *ZNC1* gene encodes a 782 amino acid protein that contains a Zn(II)_2_C_6_ fungal-type zinc finger DNA-binding domain and a leucine zipper domain. *ZNC1* transcription is elevated during yeast growth and decreases during the formation of mycelium. Cells in which *ZNC1* has been deleted show increased hyphal cell formation. Znc1p-GFP localizes to the nucleus, but mutations within the leucine zipper domain of Znc1p, and to a lesser extent within the Zn(II)_2_C_6_ domain, result in a mislocalization of Znc1p to the cytoplasm. Microarrays comparing gene expression between *znc1::URA3* and wild-type cells during both exponential growth and the induction of the yeast-to-hypha transition revealed 1,214 genes whose expression was changed by 2-fold or more under at least one of the conditions analyzed. Our results suggest that Znc1p acts as a transcription factor repressing hyphal cell formation and functions as part of a complex network regulating mycelial growth in *Y. lipolytica.*

## Introduction

Fungi are ubiquitous organisms that survive and adapt to changing environmental conditions by rapidly initiating specific physiological responses [1]. The ability of some fungi to grow as yeast-like forms or as hyphal cells, depending on the environmental conditions, is called dimorphism. Among the dimorphic fungi are plant pathogens such as *Ustilago maydis* and *Ceratocistis ulmi*, saprotrophs such as *Yarrowia lipolytica*, and human pathogens such as *Candida albicans* and *Paracoccidioides brasiliensis*. Fungal dimorphism is a complex phenomenon involving extensive modification of the cellular machinery in response to environmental signals [2]. Dimorphism has been studied in *C. albicans* [3], *U. maydis* [4], *P. brasiliensis* [5], and *Saccharomyces cerevisiae* [6], among others. The use of *S. cerevisiae* as a model may be questionable because *S. cerevisiae* it forms linear chains of elongated cells, called pseudohyphae, in which the daughter cell remains attached to the mother cell [2]. While some aspects of the dimorphic transition are mechanistically similar in these organisms, there are important differences that warrant the study of a variety of different dimorphic species to understand the complexity of the dimorphic transition [7].

*Y. lipolytica* has been used industrially to produce heterologous proteins [8] and in biotechnological applications for several processes including the degradation of fatty acids and hydrocarbons and the production of organic acids [9], [10]. Moreover, the ability of *Y. lipolytica* to grow as yeast-like cells or to form true hyphal cells, depending on the environmental conditions, has made it a useful model for studying the processes of cellular differentiation [11]. The dimorphic transition in *Y. lipolytica* is induced by various effectors, such as *N*-acetylglucosamine, serum [11], the nature of the nitrogen source [12], citrate, and the pH of the medium [13].

*Y. lipolytica* is amenable to genetic and molecular analysis [14], [15], and mutants that are unable to form hyphal cells are readily isolated morphologically [16], making *Y. lipolytica* an excellent system for studying the molecular basis of the yeast-to-hypha transition. Several *Y. lipolytica* genes involved in dimorphism have been isolated and characterized. These genes participate not only in dimorphism but also in a variety of other cellular activities. Mutations in the putative transcription factors Hoy1p and Mhy1p prevent the yeast-to-hypha transition and block mycelial growth [16], [17], while mutations in *INP1*, which is involved in peroxisome inheritance, enhance the conversion from the yeast form to the mycelial form in oleic acid-containing medium [18]. A number of genes involved in signal transduction are also involved in the dimorphic transition in *Y. lipolytica*. These include mutants of *CLA4*, which are unable to form filaments or invade agar [19], *TPK1*, which are constitutive for the mycelial form [20], *RAC1*, which show alterations in cell morphology [21], and *STE11*, which have lost the capacity to mate and to grow in the mycelial form [22]. A completely sequenced *Y*. *lipolytica* genome [23], [24] has provided the opportunity to study fungal dimorphism on a global scale and to identify new genes involved in this process.

Here, we report the isolation and characterization of *ZNC1*, a new gene involved in the dimorphic transition in *Y. lipolytica*. We have determined the pattern of *ZNC1* expression, where Znc1p is localized, and the colonial and cellular morphology of the *ZNC1* null mutant. Our results suggest that Znc1p is a transcription factor that functions as a negative regulator of *Y*. *lipolytica* filamentation. We also used transcriptome profiling to analyze the global expression of genes during the dimorphic growth of *Y. lipolytica*.

## Materials and Methods

### Strains, Media and Microbiological Techniques

The *Y. lipolytica* strains used in this study are listed in Table 1. The non-filamentous mutant strain CHY33188 was isolated after the chemical mutagenesis of E122 cells by 1-methyl-3-nitro-1-nitrosoguanidine, as previously described [17]. The strains were grown in complete medium (YEPD) or supplemented minimal medium (YNB, YNA, YNBGlc or YNBGlcNAc), as required. The components of YEPD were as follows: 1% yeast extract, 2% peptone, 2% glucose; YNB: 0.67% yeast nitrogen base without amino acids; YNA: 0.67% yeast nitrogen base without amino acids, 2% sodium acetate; YNBGlc: 0.67% yeast nitrogen base without amino acids, 1% glucose; and YNBGlcNAc: 0.67% yeast nitrogen base without amino acids, 1% *N*-acetylglucosamine, 50 mM sodium citrate, pH 6.0. For osmotic stress, the YEPD medium was supplemented with 0.4 M NaCl. The minimal medium was supplemented with uracil, leucine or lysine, each at 150 µg·ml^−1^, as required. The growth conditions and transformation of *Y. lipolytica* have been previously described [7]. The *Escherichia coli* strains DH5α and TOP10 (Invitrogen) were grown in LB broth.

**Table 1:** *Y. lipolytica* strains used in this study.

Strain	Genotype	Source
E122	MAT *leu2-270 lys8-11 ura3-302*	C. Gaillardin^a^
CHY33188	MATA *leu2-270 lys 8-11 ura3-302 fil* ^−^	This study
R9	MATA *leu2-270 lys 8-11 ura3-302 fil^+^*	This study
*Yl*JC35-7	MATA *leu2-270 lys 8-11 ura3-302 znc1*::*URA3*	This study
*Yl*JC35-16	MATA *leu2-270 lys 8-11 ura3-302 znc1*::*URA3*	This study
*Yl*AM1AR	MATA *leu2-270 lys 8-11 ura3-302 znc1*::*URA3*/*ZNC1-GFP*	This study
*Yl*AM150	MATA *leu2-270 lys 8-11 ura3-302 znc1*::*URA3*/*ZNC1(*L_437_L_438_/R_437_R_438_)-*GFP*	This study
*Yl*AM160	MATA *leu2-270 lys 8-11 ura3-302 znc1*::URA3/*ZNC1(*C_22_C_25_/R_22_R_25_)-*GFP*	This study
*Yl*AM170	CHY33188 transformed with plasmid pINA445. MATA *leu2-270 lys 8-11* *ura3-302* (*LEU2*)	This study
*Yl*AM180	CHY33188 transformed with plasmid pINA443. MATA *leu2-270 lys 8-11* *ura3-302* (*URA3*)	This study
*Yl*AM190	E122 transformed with plasmid pINA443. MATA *leu2-270 lys 8-11* *ura3-302* (*URA3*)	This study
*Yl*AM200	E122 transformed with plasmid pJCTGCAN18. MATA *leu2-270 lys 8-11* *ura3-302* (*ZNC1*) (*LEU2)*	This study
*Yl*AM210	*Yl*JC35-16 transformed with plasmid pJCTGCAN18. MATA *leu2-270 lys 8-11* *ura3-302 znc1*::*URA3* (*ZNC1*) (*LEU2)*	This study
		

a C. Gaillardin. UFR Microbiologie et Génétique Moléculaire, AgroParisTech, Centre de Biotechnologie Agro-Industrielle, Thiverval-Grignon, France.

### Hyphal Cell Induction

The yeast-to-hypha transition was stimulated by incubating the cells in YNBGlcNAc medium, as previously described [11] with minor modifications. To begin, the cells grown in YEPD medium were used to inoculate YNBGlc medium at an initial OD_600nm_ of 0.1, and these cultures were incubated at 28°C until they were in exponential growth at an OD_600nm_ of 1.8. The cells were then collected by centrifugation, resuspended in water, and incubated at 28°C for 12 h with agitation. Next, the cells were again collected by centrifugation, resuspended in YNB medium and maintained at 4°C for 12 h. Subsequently, the cells were once again recovered by centrifugation, resuspended in YNB medium and used to inoculate YNBGlcNAc medium. Finally, the cells were incubated at 28°C and samples of these cultures were removed at 15, 60 and 180 min. The incubation continued for 12 h to complete the induction of the yeast-to-hypha transition.

### Cloning and Characterization of the *Y. lipolytica ZNC1* Gene

The *Y*. *lipolytica ZNC1* gene was isolated from a *Y*. *lipolytica* genomic DNA library in the replicative *E*. *coli* shuttle vector pINA445 [25] by functional complementation of the strain CHY33188. Plasmid DNA was introduced into yeast cells by electroporation, and Leu^+^ transformants were screened on YNA agar plates for filamentous colonies after 3 days of incubation at 28°C. The complementing plasmids were recovered via the transformation of *E*. *coli* DH5α cells. The plasmid pJCTGCAN18 was recovered from one filamentous colony (R9) and both strands of the 4.8-kbp genomic insert were sequenced. The smallest fragment capable of restoring hyphal growth was a region of 2,775 bp. The deduced polypeptide sequence of Znc1p was compared to other known protein sequences using the BLAST Network Service of the National Center for Biotechnology Information.

### Deletion of the *ZNC1* Gene

A linear DNA fragment consisting of the *URA3* gene flanked by 394 bp from the 5′-upstream region and 655 bp from the 3′-downstream region of the *ZNC1* gene was used to disrupt *ZNC1* by replacing its open reading frame (ORF) with the *URA3* gene and transforming the *Y. lipolytica* strain E122 into a uracil prototroph. DNA was isolated from 23 transformants and analyzed by PCR to check for the deletion of the *ZNC1* gene. Southern blotting confirmed that the transformants *Yl*JC35-7 and *Yl*JC35-16 correctly replaced the *ZNC1* gene with the *URA3* gene through a double homologous recombination event.

### GFP Tagging of Znc1p

DNA coding for green fluorescent protein (GFP) was amplified by PCR from the plasmid pGPDAsGFP [26] using the oligonucleotides 5′-TCGATCACTAGTATGAGTAAAGGAGAAGAACTT- 3′ and 5′-ACACATACTAGTTTTGTATAGTTCATCCATGCC - 3′, which contain the *Spe*I restriction site (underlined). The PCR product was inserted into the *Spe*I site of the plasmid pJCTGCAN18 to create pJCTGCAN203, which expresses Znc1p with a C-terminal fusion to GFP (Znc1p-GFP). The construct was verified by sequencing. Plasmid pJCTGCAN203 was used to transform the *Y. lipolytica znc1::URA3* strain *Yl*JC35-16 using a one-step transformation protocol [27], and Leu^+^ transformants were recovered and checked for GFP fluorescence.

### Fluorescence Microscopy

Cell nuclei were stained with 4,6-diamidino-2-phenylindole (DAPI) (Sigma-Aldrich). The images were acquired using a Nikon Optiphot-2 microscope with a SPOT RT-Color Camera and SPOT 4.6 software (Diagnostic Instruments). GFP and DAPI fluorescence were detected using the recommended filters (Chroma Technology). GFP images were also obtained using the inverted agar block method of sample preparation [28] with a Zeiss Model LSM510 META CLSM microscope equipped with a 100 Ph3 Plan Neofluar oil immersion objective (N.A. 1.3), (Departamento de Microbiologia, Centro de Investigacion Cientifica y de Educacion Superior de Ensenada (CICESE), Ensenada, Baja California, Mexico). The images were processed and analyzed using LSM-510 v3.2 software.

### Site-directed Mutagenesis of *ZNC1*

Specific mutations in the *ZNC1* gene were made using the QuikChange II XL Site-Directed Mutagenesis Kit (Agilent Technologies). The 2.2-kbp *Sal*I-*Xba*I fragment from pJCTGCAN18 that contains the *ZNC1* gene was cloned into pBlueScript II SK(+) to make pJCTGCAN227. Two cysteine residues within the Znc1p zinc finger region were replaced by two arginines (C_22_C_25_/R_22_R_25_) using the oligonucleotides 5′-GTCACACTTGGGGCGTAAGACTCGTAAGAGACGACG-3′ and 5′-CGTCGTCTCTTACGAGTCTTACGCCCCAAGTGTGAC-3′. Random plasmids were sequenced and the mutations were confirmed, one plasmid was selected which was designated pJCTGCAN277. Similarly, two leucine residues in the leucine zipper region were replaced by two arginines (L_437_L_438_/R_437_R_438_) using the oligonucleotides 5′- CCAAACGCTTCAGATGCTCGC. CGCGTAACCACTCTCATTTTGGC-3′ and 5′-GCCAAAATGAGAGTGGTTACGCGGCG. AGCATCTGAAGCGTTTGG-3′. Random plasmids were sequenced, and the mutations were confirmed, one plasmid was selected which was designated pJCTGCAN282. The 2.2-kbp *Sal*I-*Xba*I fragment from pJCTGCAN203 that expresses Znc1p-GFP replaced the 2.2-kbp *Sal*I-*Xba*I fragment from both pJCTGCAN277 to create pJCTGCAN294 (*YlZNC1* C_22_C_25_/R_22_R_25_-GFP) and pJCTGCAN282 to create pJCTGCAN297 (*YlZNC1* L_437_L_438_/R_437_R_438_-GFP). The *znc1::URA3* strain *Yl*JC35-16 was transformed with each plasmid using a one-step transformation protocol [27], and Leu^+^ transformants were recovered and checked for GFP fluorescence. Each of the plasmids used in this study are listed in Table S11 in File S2.

### RT-PCR Analysis

The relative abundance of the *ZNC1* mRNA under different conditions of cell growth was determined using reverse transcription-PCR (RT-PCR), as previously described [29]. The *HIS1* mRNA was used as an internal control due to the constitutive expression of the *HIS1* gene. For RT-PCR, 4 µg of total RNA digested with RQ1 RNase free-DNase (Promega) was used in cDNA synthesis. PCR amplification was carried out using the SuperScript III One-Step RT-PCR System, Platinum *Taq* DNA Polymerase (Invitrogen) and either the primers ZNC1iD_3 and ZNC1iR_4 for the amplification of *ZNC1* cDNA or HIS1F and HIS1R for the amplification of *HIS1* cDNA. The sequences of the primers are listed in the Table 2. The cDNA was amplified using the following parameters: 1 cycle of 30 min at 60°C, 2 min at 94°C; 30 cycles of 1 min at 96°C, 1 min at 55°C, 1 min at 72°C; and 1 cycle of 7 min at 72°C. Aliquots from each reaction were analyzed by electrophoresis on 2% agarose gels and staining with ethidium bromide. The results were documented using the Gene Genius Imaging System (Syngene) and analyzed with Quantity One software, v4.6.9 (Bio-Rad).

**Table 2:** Primers used in the RT-PCR assays.

Gene	Primer	Sequence (5′to 3′)	Fragment (bp)	Reference
*ACT1*	ACT20	TCCAGGCCGTCCTCTCCC	141	a
	ACT21r	GGCCAGCCATATCGAGTCGCA		
*BEM1*	BEMIF	GTGGACACAGAGGTCATTC	600	b
	BEMIR	CTGGACCTCTCGTTGTAGC		
*BMH1*	BMH1F	GGTCAACTACATGAAGGACG	600	c
	BMH1R	AATGACGGTAGAGTCTCGG		
*BMH2*	BMH2F	CGTTACGAAGACATGGTGG	600	c
	BMH2R	CAATGTCAGCAATGGCATCG		
*CLA4*	CLA4iD_3	ACCGACGCACAGATCACCGAGAAG	506	d
	CLA4iR_4	TTGGCGCAGAAACCGAAATCAGTA		
*HIS1*	HIS1F	TCAAGTTTGTCGGAGGCTC	400	b
	HIS1R	CCAGAATGTCACTAGCACC		
*HOY1*	HOY1iD_3	GAAGGCTATGCGCGGTCGGGAATCT	355	d
	HOY1iR_4	ATGCGGTGTGGTGGGGGTCAGGTTA		
*MHY1*	MHY1iD_3	CCTCCCCGGCCTCCACTTACT	329	d
	MHY1iR_4	GGCGAGATTGTCCGACCGAGAAA		
*RKA1*	RKA1iD_3	GAGCCTGCTGCGCCCTTCAC	663	d
	RKA1iR_4	CTCGCCCTTCTTAGTCACCTCAGC		
*STE7*	STE7iD_3	GGCCCGCTGACCGAAGAGATT	526	d
	STE7iR_4	TGGCCCGAGACGTGATGATAAAAG		
*STE11*	STE11iD_3	CCTCCCCACAGCGCTACCCCTTCC	480	d
	STE11iR_4	ATTGGCGCCCTTGATGTCTCG		
*STE12*	STE12iD_3	CAGCCGGCTCTACCTCCTTCCTCACAGT	112	d
	STE12iR_4	CGCCCCGCTCGTCTCGCTCAA		
*TPK1*	TPK1iD_3	ACCCCGTGGCCAAGTTTTATGC	555	d
	TPK1iR_4	TGTCGCCCACTCCACTCTGAATG		
*ZNC1*	ZNC1iD_3	ATGTCCTCCACCAAACCACGCAAGTTCC	297	d
	ZNC1iR_4	AGGCGGTGGTGGAGCTGGATAGCCATAG		

a [30],

b [7],

c [29],

d This study.

This technique was also used to validate and measure the expression of some genes from the microarray assay relative to the expression of the *ACT1* gene. The experiment was performed under the same parameters as described above. The following primers were used: ACT20 and ACT21r for *ACT1* [30], BEMIF and BEMIR for *BEM1* [7], BMH1F and BMH1R for *BMH1* [29], BMH2F and BMH2F for *BMH2* [29], CLA4iD_3 and CLA4iR_4 for *CLA1*, HOY1iD_3 and HOY1iR_4 for *HOY1*, MHY1iD_3 and MHY1iR_4 for *MHY1*, RKA1iD_3 and RKA1iR_4 for *RKA1*, STE7iD_3 and STE7iR_4 for *STE7*, STE11iD_3 and STE11iR_4 for *STE11*, STE12iD_3 and STE12iR_4 for *STE12* and TPK1iD_3 and TPK1iR_4 for *TPK1*. The sequence of each primer is listed in the Table 2.

### RNA Extraction and Northern Blot

Total RNA was prepared using TRIzol (Invitrogen) according to the manufacturer’s instructions. Prehybridization and hybridization were performed according to standard procedures, and a radiolabeled DNA fragment of *ZNC1* was used as a probe [31].

### Microarrays

For transcriptome analysis, we used 12 sampling points to compare gene expression between the *znc1::URA3* and wild-type cells during exponential growth and during the induction of the yeast-to-hypha transition. For the transcriptional profiling of cells in exponential growth, cells were grown in YEPD medium at 28°C until they achieved an OD_600_ = 1.8. For the transcriptional profiling of cells undergoing the yeast-to-hypha transition, cells were grown in a 1% *N*-acetylglucosamine and 50 mM citrate buffer, pH 6.0, at 28°C and sampled at 15, 60 and 180 min.

Frozen samples were mechanically disrupted with a bead beater in a 5 ml Teflon vessel (Mikro-Dismembrator, B. Braun, Biotech) with a tungsten carbide bead (∅∼7 mm) for 2 min at 2000 rpm. The RNA was extracted from the resulting powder with the TRIzol Reagent (Invitrogen). The quality of the RNA was visualized on a 1% TBE-agarose gel, and the quantity of the RNA was determined by measuring its absorbance at 260 nm. The mRNA obtained was reverse transcribed and labeled using the CyScribe First-Strand cDNA Labeling kit (Amersham GE Healthcare). Each sample was labeled with Cy5, and a mixture of all of the RNA samples was labeled with Cy3 and used as a reference. The resulting labeled cDNAs were further purified using the Illustra CyScribe GFX Purification kit (GE Healthcare) and the eluate was concentrated using Microcon-YM50 columns (Millipore). Eurogentec (Belgium) designed the microarray probes and produced the slides. The samples were hybridized manually using 5 µl each of the Cy3- and Cy5-labeled cDNA and the single-stranded DNA from salmon testes (Sigma-Aldrich). The DNA and cDNA were mixed, denatured at 95°C for 2 min, and quickly chilled on ice. Then, 35 µl of the hybridization buffer DIG Easy Hyb (Roche) was added, and the solution was incubated with a microarray slide and covered with a rimmed cover glass (LifterSlip, 22×50 mm, Erie Scientific). The slides were placed in a hybridization chamber (Corning) and hybridized for 12 h at 42°C by immersion in a water bath. After washing and drying, the microarrays were scanned using a GenePix 4000B Microarray Scanner (Molecular Devices) at a resolution where the Cy5/Cy3 average ratio was near 1. The Cy3 and Cy5 fluorescence signals were measured at 532 nm and 635 nm, respectively, with the GenePix Pro v4.0 software (Molecular Devices, USA).

### Data Filtration, Normalization and Analysis

The raw transcriptomic data were filtered and normalized with the GeneSpring GX software (Agilent Technology). A preliminary filtration of the dataset was performed with the quality flags provided by GenePix software. First, the scanned images of each microarray were visually inspected with the GenePix software. The spots with low quality data, such as a lack of uniformity or little difference between the intensity of the signal and the background of the microarray were labeled. Spots with a quality flag value below “0″ were removed from the analysis. Next, the fluorescence values obtained for each fluorophore were imported into the data set and the values tagged as invalid during the previous inspection were eliminated. The remaining data were subjected to an intensity-dependent normalization (Lowess).

Data from at least three independent experiments, including one dye-swap experiment, were evaluated using GeneSpring GX software. The Cy5/Cy3-ratios were first normalized based on their overall fluorescence intensity then were exported into Excel spreadsheets. Because each ORF was present twice on each array and three independent experiments were performed, at least six signal intensity ratios reflecting the activity of a test sample to a reference sample were compared.

To determine which genes are differentially expressed, two filters were applied to the normalized data. The non-parametric Wilcoxon-Mann-Whitney test was applied first followed by the Benjamini-Hochberg false discovery rate (p value ≤ 0.02). These tests identified the genes that are differentially expressed in the mutant strain compared to the parental strain with a probability of error of 2% for the set of selected genes.

Among the genes identified by the statistical test, only those that had a variation mutant vs. parental strain ≥ 2.0, a filter for differential expression commonly used in publications on microarrays, were considered. Each gene was required to have at least a two-fold change in expression to be labeled as significantly regulated. Cluster analysis and Venn diagrams were produced using the GeneSpring GX software with the standard settings. Gene designations were based on annotations in Génolevures (http://www.genolevures.org/). The functional classification of the genes was performed according to the gene ontology terms defined during the genome annotation of *Y. lipolytica* and by comparisons with the homologous genes in *S. cerevisiae.*

### Microarray Data Accession Number

The array data are available from the ArrayExpress database (http://www.ebi.ac.uk) under the accession number E-MTAB-517 (http://www.ebi.ac.uk/arrayexpress/experiments/E-MTAB-517).

### Nucleotide Sequence Accession Number

The sequence data reported here are available from EMBL/GenBank/DDBJ under the accession number AJ575099. The nucleotide sequence of the *ZNC1* gene corresponds to the ORF YALI0B05038g in the *Y. lipolytica* genome (http://www.genolevures.org/).

## Results

### Isolation and Characterization of the *ZNC1* Gene

**Figure 1:**
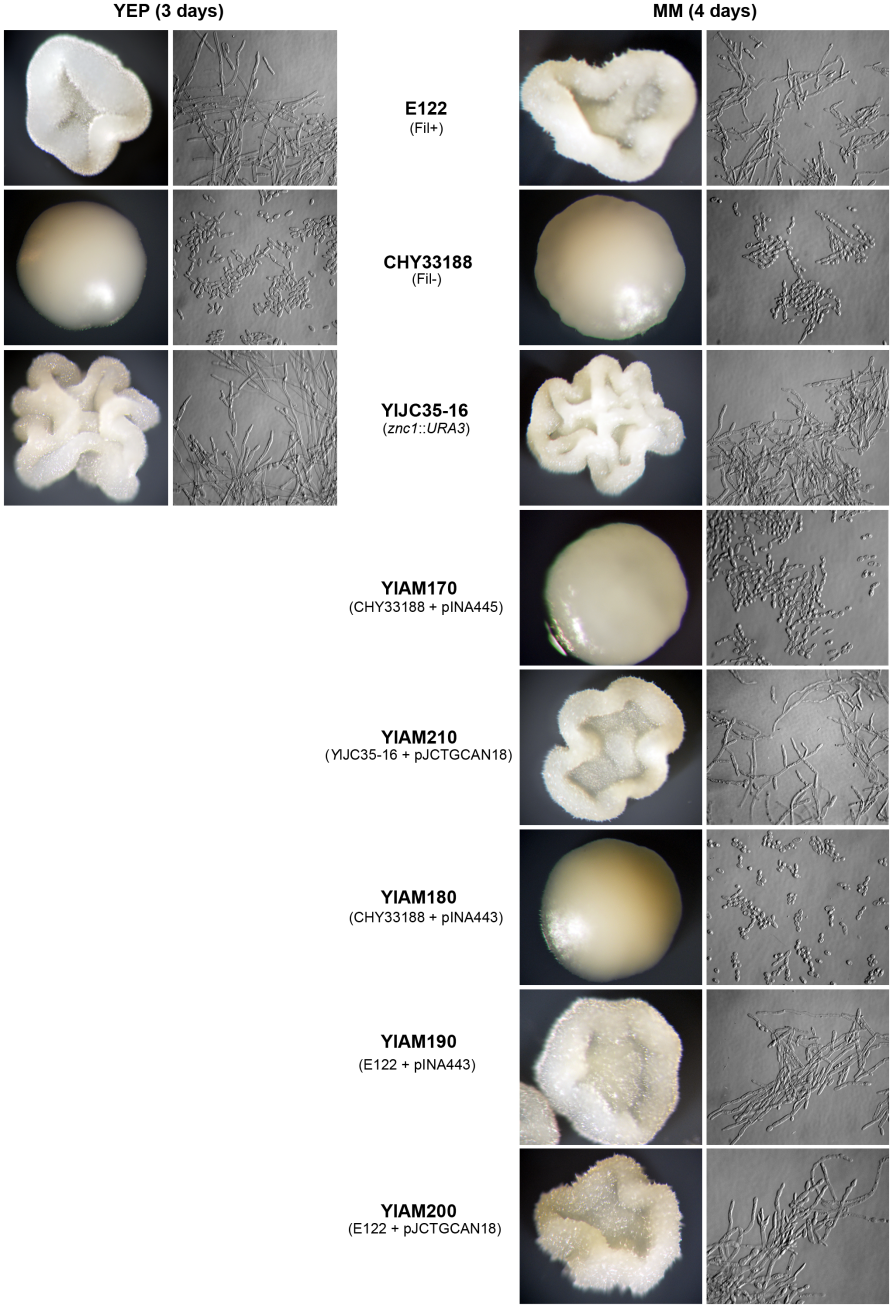
*Y. lipolytica* colony and cell morphology. Wild-type strain **E122**. Mutant strain **CHY33188.** Strain ***Yl*** **JC35-16** (*znc1::URA3).* Strain ***Yl*** **AM170** (mutant strain CHY33188 transformed with pINA445). Strain ***Yl*** **AM210** (*Yl*JC35-16 transformed with pJCTGCAN18). Strain ***Yl*** **AM180** (mutant strain CHY33188 transformed with pINA443). Strain ***Yl*** **AM190** (Wild-type strain E122 transformed with pINA443). Strain ***Yl*** **AM200** (Wild-type strain E122 transformed with pJCTGCAN18). Colonies and cell morphology were imaged after 3 days of incubation on YEPD agar plates at 28°C.

The *Y. lipolytica* non-filamentous mutant strain CHY33188 was isolated by the chemical mutagenesis of wild-type E122 cells and identified by its inability to form wild-type rough-surfaced colonies on YEPD agar plates after 3 days of incubation at 28°C (Fig. 1). Further experiments revealed that the CHY33188 strain was able to grow only as the yeast form in both rich medium and minimal medium supplemented with the hyphal cell inducer *N*-acetylglucosamine. The CHY33188 strain was then transformed with a *Y. lipolytica* genomic DNA library contained in the replicative *E. coli* shuttle vector pINA445 [17]. Of approximately 19,700 Leu^+^ transformants, four formed rough-surfaced colonies that displayed the filamentous phenotype. One recovered plasmid, pJCTGCAN18, contained a 4.8-kbp fragment capable of restoring filamentous growth to CHY33188 cells. Sequencing both strands of this fragment revealed a 2,349-bp ORF that restored filamentous growth to CHY33188 cells. This ORF sequence is contained at locus YALI0B05038g of the complete *Y. lipolytica* genome (www.genolevures.org). A putative TATA box (TATATA) was found between nucleotides −174 and −179 relative to the A nucleotide of the potential start codon. The 5′-upstream region contains consensus sequences for elements implicated in fungal nutrition and the regulation of development, including four copies of the stress response sequence CCCCT [32] and the sequence TGACT, which is implicated in binding the transcriptional activator Gcn4p in *S. cerevisiae* [33]. The 3′-downstream region contains a typical transcriptional termination motif (TAG…TATGT…TTTT) and the signal for polyadenylation (TAATAAA). The resulting protein is 782 amino acids in length (Fig. S1) and has a predicted molecular mass of 85,544 Da and an isoelectric point of 7.36. Analysis of the deduced protein sequence using PROSITE (www.expays.org) revealed a Zn(II)_2_C_6_ fungal-type DNA-binding domain (amino acids 21–51), a bipartite nuclear localization signal profile (amino acids 16–30), a proline-rich region (amino acids 94–253), a histidine-rich region (amino acids 189–209), and a leucine zipper region (amino acids 422–443) (Fig. S1). The Zn(II)_2_C_6_ fungal-type DNA-binding domain has the typical CysX_2_CysX_6_CysX_5-12_CysX_2_CysX_6-8_Cys pattern [34], and the leucine zipper domain has a LeuX_6_LeuX_6_LeuX_6_Leu pattern. We have redesignated the ORF at locus YALI0B05038g as the gene *ZNC1* and suggest that it encodes a potential transcription factor.

### *ZNC1* mRNA Levels Decrease During the Yeast-to-hypha Transition

**Figure 2:**
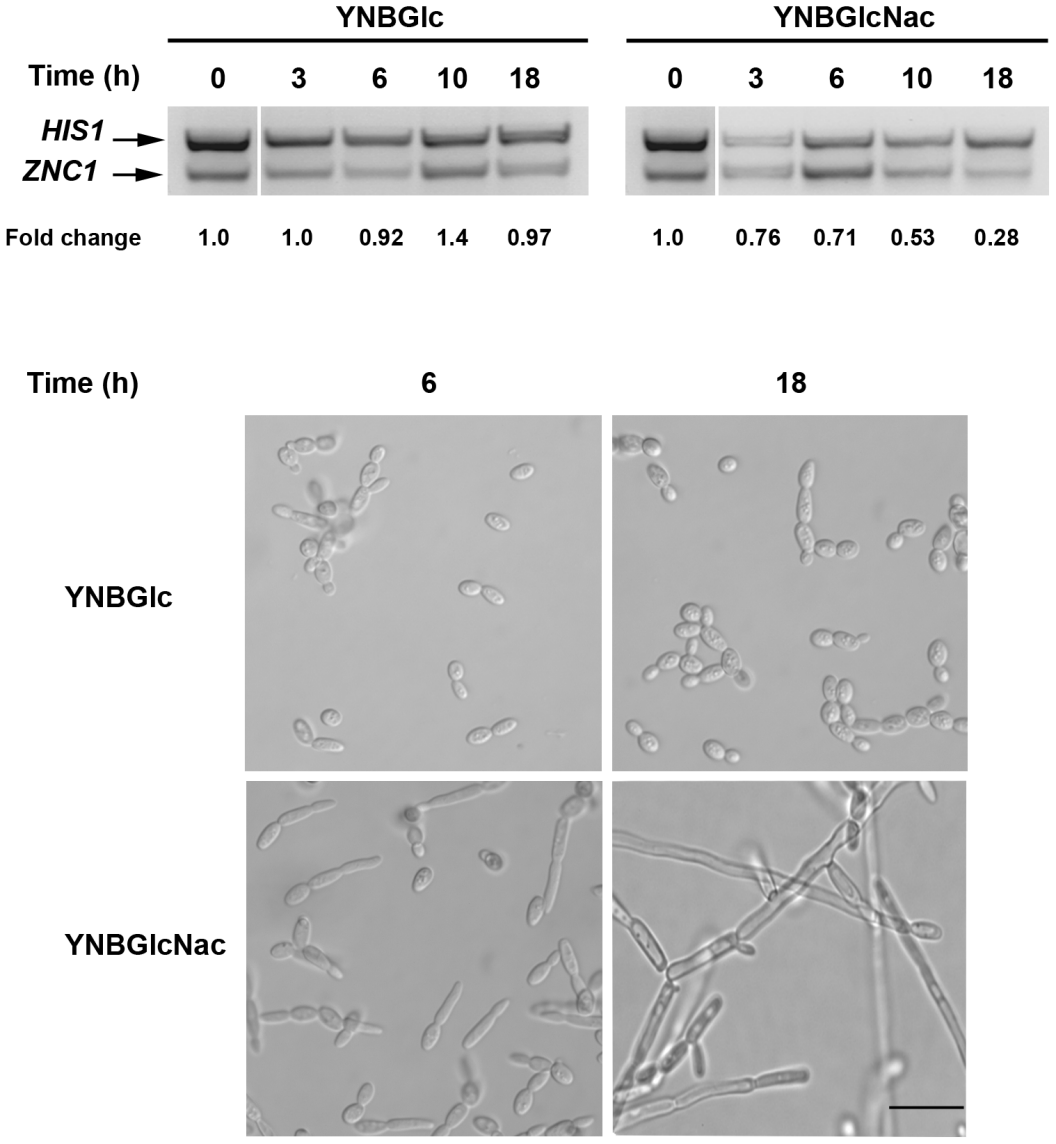
Expression analysis of the *ZNC1* gene during the dimorphic transition. *Y. liplolytica* E122 cells were incubated at 28°C in YNBGlc medium to form yeast-like cells or in YNBGlcNAc medium to induce the yeast-to-hypha transition. At specific times, total RNA was isolated and used for RT-PCR to compare the quantities of Z*NC1* transcripts. *HIS1* was used as a loading control. The micrographs show the morphology of cells at specific times. The bar in the micrographs = 10 µm.

RNA extracted from cells harvested after 0, 3, 6, 10 and 18 h of incubation in YPD medium was subject to Northern blotting using a *ZNC1* gene fragment, as a probe, revealing a single RNA species of approximately 2.6-kb (data not shown). To analyze *ZNC1* mRNA levels during the yeast-to-hypha transition, we performed RT-PCR using *HIS1* mRNA as an endogenous internal standard because its expression levels are constant during the yeast-to-hypha transition [7]. The dimorphic transition was induced in exponentially growing E122 cells cultured in YNBGlc medium for 15 h and the cells were then inoculated at a final density of 10^5^ cells·ml^−1^ in YNBGlcNAc medium for the induction of the yeast-to-hypha transition or in fresh YNBGlc medium. RT-PCR revealed that the *ZNC1* mRNA levels were reduced during the formation of hyphae cells (Fig. 2).

### Deletion of the *ZNC1* Gene Increases the Formation of Hyphal Cells

**Figure 3:**
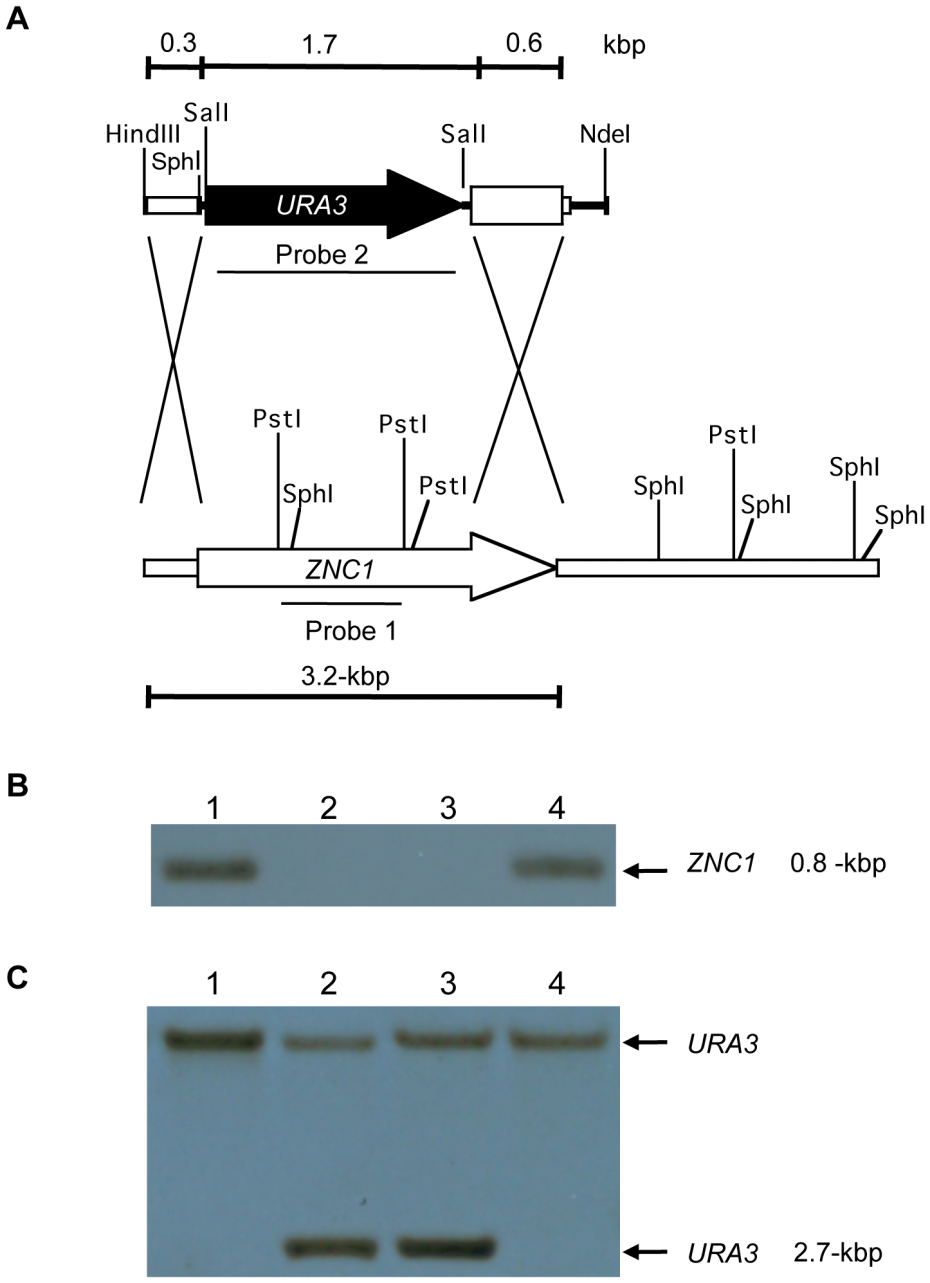
Disruption of the *ZNC1* gene. (**A**) Schematic representation of the *ZNC1* gene replacement strategy. Arrows represent the *URA3* and *ZNC1* ORFs. (**B**) Southern blot of genomic DNA from the wild-type strain E122 (lanes 1 and 4) and the *znc1::URA3* strains *Yl*JC35-7 (lane 2) and *Yl*JC35-16 (lane 3) digested with *Pst*I. A fragment of the *ZNC1* gene was used as the probe. (**C**) Southern blot of genomic DNA from the wild-type strain E122 (lanes 1 and 4) and the *znc1::URA3* strains *Yl*JC35-7 (lane 2) and *Yl*JC35-16 (lane 3) digested with *Sph*I. A fragment of the *URA3* gene was used as the probe.

To analyze the *ZNC1* null mutant phenotype, the *ZNC1* gene was replaced by a 1.7-kbp *Sal*I-*Sal*I fragment containing the *URA3* gene flanked by 394-bp and 655-bp fragments from the 5′- and 3′-ends, respectively, of the *ZNC1* gene. This linear construct was used to transform the strain E122 into a uracil prototroph. Several smooth colonies were analyzed by PCR and found to contain an intact *ZNC1* gene. Twenty-three colonies with a rough phenotype were then analyzed and PCR and Southern blotting (Fig. 3B and 3C) showed that two colonies, *Yl*JC35-7 and *Yl*JC35-16, had the *ZNC1* gene replaced by the *URA3* construct. The 0.8-kbp *Pst*I fragment from the wild-type *ZNC1* gene in the E122 strain (Fig. 3B, lanes 1 and 4) was not observed in either transformant (Fig. 3B, lanes 2 and 3). When the *URA3* gene was used to probe the *Sph*I-digested genomic DNA, only a single fragment was observed in the E122 strain (Fig. 3C, lanes 1 and 4), but two fragments corresponding to the *URA3* gene and the expected integration of *URA3* at the *ZNC1* locus were observed in both transformants (Fig. 3C, lanes 2 and 3).

To determine if deleting *ZNC1* affects growth in different media, wild-type E122, mutant CHY33188 and *Yl*JC35-16 cells were grown in YEPD pH 4, YEPD pH 7, YEPD medium supplemented with 0.4 M NaCl, minimal medium, minimal medium containing glucose or acetate and minimal medium containing *N*-acetylglucosamine. Strain E122 was able to grow as yeast or hyphal cells depending on the medium. In contrast, the CHY33188 mutant strain always grew as yeast cells. Contrary to expectations, the deletion of the *ZNC1* gene increased the formation of hyphal cells in the *Yl*JC35-16 strain, and filamentous growth was observed under all conditions (Fig. 1). In medium containing *N*-acetylglucosamine, the *Yl*JC35-16 strain formed hyphal cells earlier than the E122 strain did. The *znc1::URA3* deletion strain *Yl*JC35-16 is hyperfilamentous, regardless of growth conditions. This unexpected hyperfilamentous phenotype for the *znc1::URA3* strain led us to sequence the *ZNC1* gene from the mutant CHY33188 strain, which shows a non-filamentous phenotype. No nucleotide change was found in the *ZNC1* gene from the wild-type E122 and mutant CHY33188 strains, suggesting that there is a secondary mutation in the CHY33188 strain that is suppressed by *ZNC1*. Transformation of the *znc1::URA3* strain with the pJCTGCAN18 plasmid that contains the *ZNC1* gene (strain YlAM210) restored the wild-type phenotype (Fig. 1). In *C. albicans*, has been reported that during knockout of some genes, the phenotypic changes can be associated whit the selective *URA3* marker, rather than the target gene [35]. Until now, this process has not been described in *Y. lipolytica*; however, we performed the control experiments. Transformation of the mutant strain CHY33188 with either the plasmid pINA445 (strain *Yl*AM170) that contains the *LUE2* gene or the plasmid pINA443 (strain *Yl*AM180) that contains the *URA3* gene did not change the non-filamentous phenotype (Fig. 1). Similarly, transformation of strain E122, with either the plasmid pINA443 (strain YlAM190) that contains the *URA3* gene or the plasmid pJCTGCAN18 (strain YlAM200) that contains the *ZNC1* gene did not influence the wild-type phenotype (Fig. 1).

### Zn(II)2C6 and Leucine Zipper Domains Influence the Subcellular Localization of Znc1p

**Figure 4:**
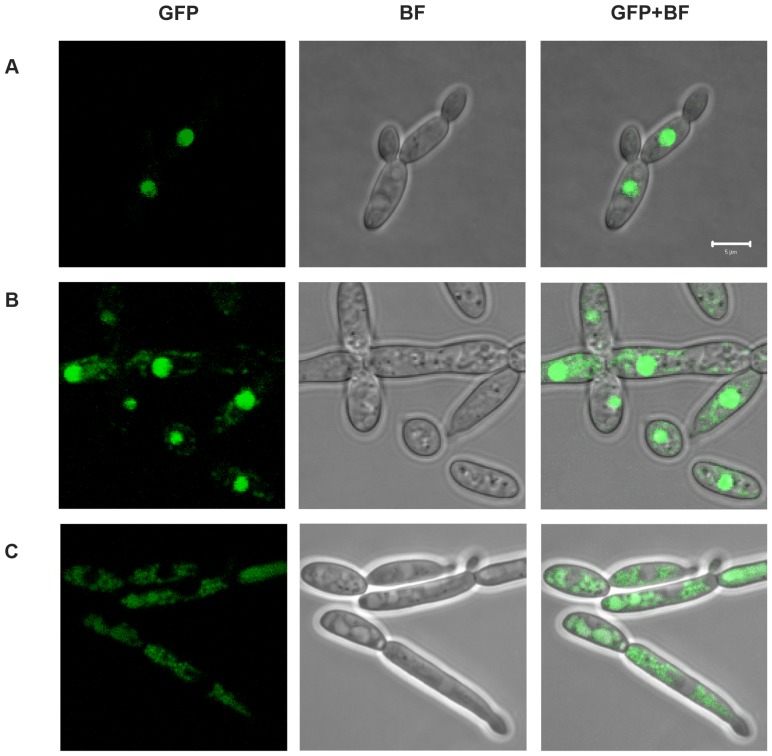
Znc1p localizes to the nucleus. (**A**) The *znc1::URA3* strain YlJC35-16 that was transformed with pJCTGCAN203 expressing Znc1p-GFP was observed by confocal laser scanning microscopy. (**B**) The *znc1::URA3* strain YlJC35-16 that was transformed with pJCTGCAN294 expressing the Znc1p-GFP mutant with cysteines 22 and 25 within the zinc-finger domain replaced with arginines was observed by confocal laser scanning microscopy. (**C**) The *znc1::URA3* strain YlJC35-16 that was transformed with pJCTGCAN297 expressing the Znc1p-GFP mutant with leucines 437 and 438 within the leucine zipper replaced with arginines was observed by confocal laser scanning microscopy. The cells were visualized using the GFP filter (GFP) or bright field (BF); merge of GFP and bright-field images (BF+GFP). The bar in the micrographs = 5 µm.

We assessed the subcellular localization of Znc1p to determine its putative role as a transcription factor. The *znc1::URA3* strain, *Yl*JC35-16, was transformed with pJCTGCAN203, which contains the *ZNC1* gene fused at its 3′-end to the *GFP* gene (*ZNC1*-*GFP*) to express Znc1p-GFP. Znc1p-GFP colocalized with the nuclear DAPI stain (data not shown). Znc1p-GFP-expressing cells were grown on solid YNBGlc medium and subjected to live-cell imaging using laser scanning confocal microscopy (Fig. 4A and Movies S1 and S2). The results confirmed that Znc1p is localized to the nucleus.

Znc1p contains two putative DNA binding domains: a Zn(II)_2_C_6_ zinc finger and a leucine zipper. Two site-directed mutations were made in these domains to determine if they play a role in the subcellular localization of Znc1p. The cysteine residues C_22_ and C_25_ within the Zn(II)_2_C_6_ zinc finger domain were replaced with arginine (R) in pJCTGCAN294 (*YlZNC1* C_22_C_25_/R_22_R_25_-GFP), and the leucine residues L_437_ and L_438_ within the leucine zipper were replaced with arginine in pJCTGCAN297 (*YlZNC1* L_437_L_438_/R_437_R_438_-GFP). The *znc1::URA3* strain *Yl*JC35-16 was transformed individually with each plasmid, the Leu^+^ transformants were recovered, and the subcellular localization of the mutant Znc1p-GFP chimeras was determined by laser scanning confocal microscopy. The GFP-tagged zinc finger mutant of Znc1p still localized primarily to the nucleus but was also weakly detected in the cytoplasm (Fig. 4B); conversely, the GFP-tagged leucine zipper mutant of Znc1p was found almost exclusively in the cytoplasm (Fig. 4C). Overall, the zinc finger domain and especially the leucine zipper of Znc1p are important for its correct localization to the nucleus.

### Comparison of the Transcriptional Profiles of Wild-type and *znc1*::URA3 Cells using Microarrays

Due to the increased hyphal cell formation in the *znc1::URA3* strain, we hypothesized that *ZCN1* represses the expression of genes required for *Y. lipolytica* filamentous growth. To understand the role of Znc1p in the gene regulatory network that is required to produce hyphal cells and to obtain a global view of the genes regulated by Znc1p during the yeast-to-hypha transition, we compared the transcriptional profiles of the *znc1::URA3* strain to that of the wild-type E122 strain using microarrays. Because we were especially interested in the potential role of *ZNC1* in the regulation of hyphal cell formation, we compared the transcriptional profiles of *znc1::URA3* cells versus E122 cells in exponential growth and at 15, 60 and 180 min during the yeast-to-hypha transition as induced by *N*-acetylglucosamine. The genes were annotated according to the Génolevures database for *Y. lipolytica* (http://www.genolevures.org/yali.html), in which the majority of *Y*. *lipolytica* genes have been assigned names and functions based on the homology of their encoded proteins to *S. cerevisiae* and *C. albicans* proteins. Genes whose encoded proteins do not share a significant level of similarity to known proteins have been designated with a systematic locus tag description. Only genes whose average expression in *znc1::URA3* cells versus wild-type E122 cells changed by ≥ 2-fold, a differential expression filter commonly applied in microarray analysis [36], [37], were considered.

**Figure 5:**
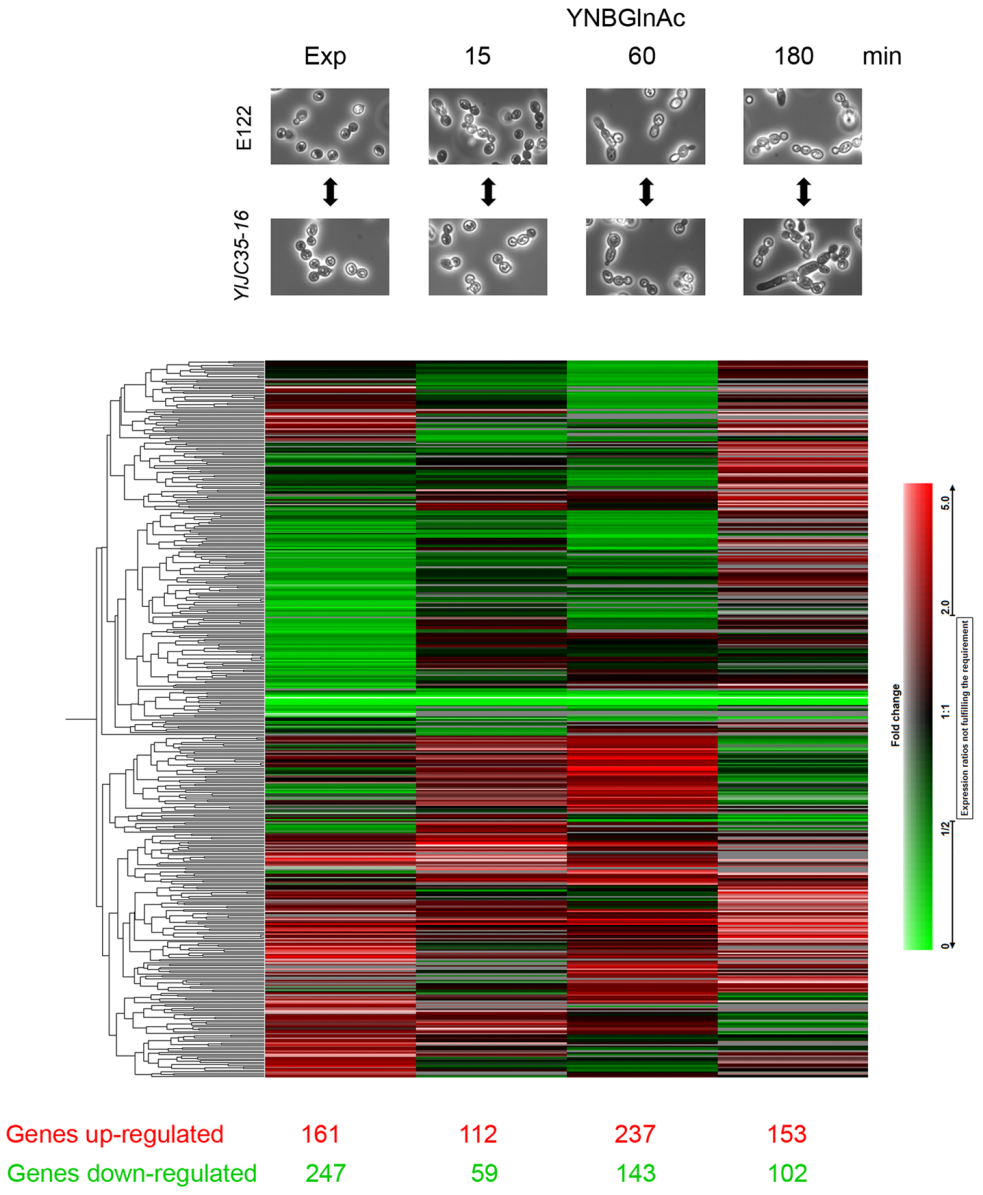
A two-way hierarchical cluster analysis of genes displaying ≥ 2-fold changes in expression level. The heat map shows the fold change in gene expression between the *znc1::URA3* strain *Yl*JC35-16 and the wild-type strain E122. The micrographs at the top compare the cell morphology of the E122 strain and the *znc1::URA3* strain during exponential growth (Exp) in YEPD medium or at the given time of incubation in *N*-acetylglucosamine medium (YNBGlnAc) for induction of dimorphic transition.

Figure 5 shows the number of differentially expressed genes for the *znc1::URA3* strain compared to the wild-type E122 strain for each condition as well as the hierarchical clustering of the different transcriptional profiles. The name and description of each gene are given in Tables S1-S8 in File S1. During exponential growth, 161 genes were up-regulated and 247 genes were down-regulated in the *znc1::URA3* strain. Most of the up-regulated genes are involved in metabolism (20.2%) or encode proteins with catalytic activity (18.2%) (Fig. S2). Similarly, most of the down-regulated genes are involved in metabolism (22.6%) or encode proteins with catalytic activity (24.6%) (Fig. S2).

During incubation in *N*-acetylglucosamine to induce the yeast-to-hypha transition, the greatest changes in gene expression were observed at 60 min, with 237 genes up-regulated and 143 genes down-regulated in the *znc1::URA3* strain. During this early step of the yeast-to-hypha transition (15, 60, and 180 min), we observed an increase in the percentage of up-regulated genes involved in metabolism (19.5% at 15 min, 28.2% at 180 min), those with catalytic activity (19.5% at 15 min, 21.4% at 180 min) and those with transporter activity (6.5% at 15 min, 16.2% at 180 min) (Fig. S2). Conversely, we observed a decrease in the percentage of up-regulated genes involved in cell organization and biogenesis (7.8% at 15 min, 3.4% at 180 min), transcription factor activity (7.8% at 15 min, 3.4% at 180 min) and signal transduction (3.9% at 15 min, 0.9% at 180 min) (Fig. S2). The percentage of down-regulated genes involved in transcription factor activity (16.7% at 15 min, 28.6% at 180 min) and signal transduction (0% at 15 min, 7.1% at 180 min) was increased (Fig. S2). Based on these data, we can speculate that the family of genes involved in cell organization and biogenesis, with transcription factors and in signal transduction activity may be important at the beginning of the hypha development. After the dimorphic pattern is initiated, the genes related to metabolism and transporter and catalytic activity become the principal factors.

Venn diagrams of the genes commonly up- and down-regulated during the yeast-to-hypha transition showed that 22 genes were up-regulated at both 15 and 60 min, 14 genes at both 60 and 180 min, and 2 genes at both 15 and 180 min (Fig. S3 and Table S9 in File S1). These two genes are YALI0F13937g, which encodes a protein similar to the *C. boidinii* NAD-dependent formate dehydrogenase, and YALI0C20251g, which is similar to the *S. cerevisiae GRE2* gene, a stress-response gene implicated in filamentous growth [38], [39]. Only three genes were up-regulated in *znc1::URA3* cells at 15, 60 and 180 min after induction: YALI0C11165g and YALI0B08426g, which encode hypothetical proteins, and YALI0E23859g, which is similar to the *S. cerevisiae PHO89* gene encoding a phosphate transporter that acts in the early phase of cell growth [40].

Ten genes were down-regulated in the *znc1::URA3* strain at both 15 and 60 min of incubation in *N*-acetylglucosamine (Fig. S3 and Table S10 in File S1). YALI0C15004g, encoding a protein with no similarity to any characterized protein, was the only gene down-regulated in *znc1::URA3* cells at 60 and 180 min of induction. Nine genes were down-regulated at 15, 60 and 180 min, including YALI0E05819g, which encodes a protein similar to the *S. cerevisiae STA1*-encoded extracellular enzyme glucan 1,4-α-glucosidase, and YALI0E11517g, which encodes the *Y. lipolytica* homolog of the *S. cerevisiae* cell wall protein encoded by *CWP1*.

**Figure 6:**
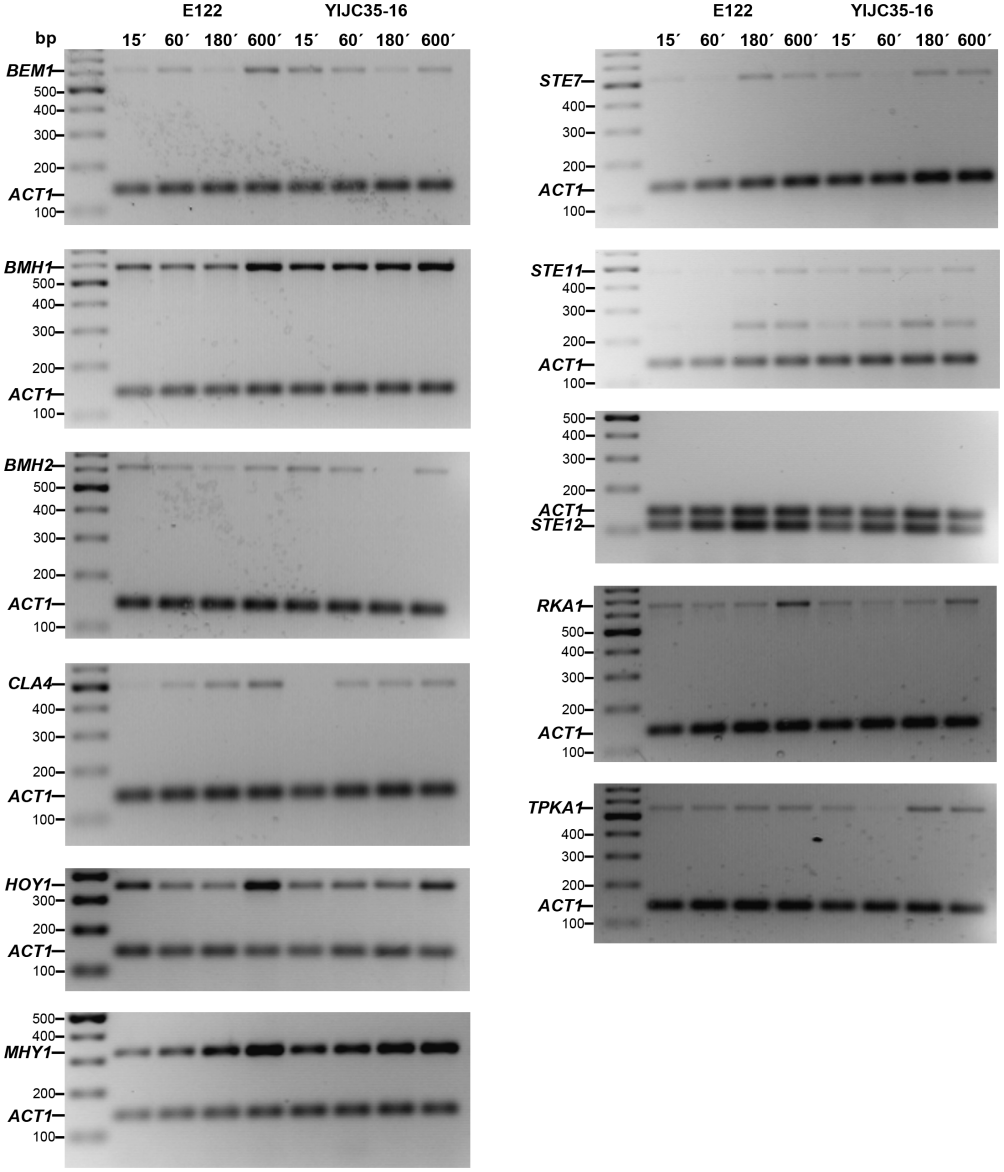
Gene expression during the dimorphic transition. *Y. liplolytica* E122 and *Yl*JC35-16 cells were incubated at 28°C in YNBGlcNAc medium to induce the yeast-to-hypha transition. At 15, 60, 180 and 600 min, total RNA was isolated and used for semi-quantitative RT-PCR analysis to compare the abundance of transcripts. *ACT1* was used as a loading control.

Of the genes previously reported to be involved in the dimorphic transition in *Y. lipolytica*, our analysis identified only two of them. The *STE12* gene is up-regulated at 15′ post-induction (fold change = 2.24) and the *HOY1* gene is up-regulated at 180′, post-induction (fold change = 2.62). To analyze how the lack of the gene *ZNC1* affects the expression levels of the genes involved in the dimorphic transition induced by N-acetylglucosamine, we obtained microarray data on 11 of these genes and performed RT-PCR assays during the dimorphic transition and compared their data with the microarray expression levels. Figure 6 shows the expression levels during the yeast-to-hypha transition at 15, 60, 180 and 600 min of incubation in *N*-acetylglucosamine. We included an additional time point at 600 min to compare the expression level with that previously reported for some of the genes listed. Table 3 shows a comparison between microarray data and the RT-PCR densitometry. Based on these data, we speculate that Znc1p is most likely not involved in controlling the expression of *HOY1* and *MHY1*, which encode putative transcription factors, or the *TPK1* gene, which encodes the catalytic subunit of PKA, because their expression levels increased in *znc1::URA3* cells during the yeast-to-hypha transition. This result has previously been reported for wild-type cells [16], [17], [20]. The expression level of *STE12* decreased in *znc1::URA3* cells, and the expression level of *BMH2* decreased in the same cell type during the dimorphic transition. This result is different from what was previously reported for wild-type cells [21], [29], [41], suggesting that these genes could be regulated by Znc1p. Genes whose expression levels showed changes between the *znc1::URA3* and wild-type E122 strains include *STE7*, *STE11*, *CLA4, BMH1* and *RKA1*, suggesting they may also be regulated by Znc1p. The expression level of the *TUP1* gene, which in *C. albicans* encodes a transcription factor that represses the genes responsible for initiating filamentous growth [42], [43], was unaffected. Together, these data illustrate that a complex gene network involving distinct regulatory pathways functions in *Y. lipolytica* during mycelial growth.

**Table 3:** Quantitative comparison of the expression levels of the genes selected from microarrays by RT-PCR.

	Fold change (Arrays)			Fold change (RT-PCR)		
Gene	15′	60′	180′	15′	60′	180′
*BEM1*	1.33	0.83	1.03	2.74	1.11	1.12
*BMH1*	1.23	0.88	0.92	1.02	1.55	1.61
*BMH2*	1.53	1.14	0.74	0.78	1.14	0.37
*CLA4*	1.24	1.00	0.93	0.78	1.23	0.66
*HOY1*	0.99	1.34	2.62	0.67	1.14	1.36
*MHY1*	0.88	0.52	1.65	1.13	1.13	1.06
*RKA1*	1.42	1.08	0.93	0.73	0.71	0.91
*STE7*	0.74	1.33	0.91	0.75	0.75	0.59
*STE11*	1.10	1.29	1.11	1.11	2.54	0.46
*STE12*	2.24	0.76	0.45	0.94	0.94	0.85
*TPK1*	0.81	0.61	1.56	0.79	0.28	1.89

## Discussion

The formation of yeast or hyphal cells in the dimorphic yeast *Y. lipolytica* depends on different stimuli including the nature of the carbon source, the presence of serum, or the pH of the culture medium [11], [13]. Several genes regulating dimorphic growth in *Y. lipolytica* have been identified. We now report the isolation of a new gene, *ZNC1*, involved in dimorphism in *Y. lipolytica* and provide an initial characterization of its protein product. Znc1p contains a zinc finger Zn(II)_2_Cys_6_-type domain, a leucine zipper domain, a putative nuclear localization signal (KRRRIK), and several sites for potential phosphorylation. Based on these domains, we predict that Znc1p functions as a Gal4p-type transcription factor. According to MacPherson *et al.* [34], the zinc cluster proteins can be categorized in two groups: those that are permanently present in the nucleus and those that reside in the cytoplasm and must be imported into the nucleus. Znc1p-GFP was found to localize to the nucleus relatively quickly. Once the nuclei begin to divide, GFP fluorescence is detected in the cytoplasm, but it relocalizes to the nucleus after division is complete. Thus, Znc1p belongs to the first group of transcription factors that are always into the nucleus. Mutation of the zinc-finger domain in Znc1p did not dramatically change its localization, but mutation of the leucine zipper domain resulted in mislocalization of Znc1p-GFP to the cytoplasm. In both mutations, the nuclear localization signal (NLS) remained unaltered. The presence of a leucine zipper motif in transcription factors from different organisms has been hypothesized to be involved in dimerization, DNA-binding and nuclear localization [34], [44]. The mislocalization of Znc1p-GFP by the mutation of the leucine zipper domain, may be due to compromised protein dimerization, and demonstrates the importance of Znc1p dimer formation for the nuclear localization of the protein. Dimerization of proteins by the leucine zipper domain and its influence on nuclear localization have been reported for other transcription factors. The sterol regulatory element-binding protein 2 (SREBP-2) exists as a stable dimer, and the replacement of leucine residues with alanine residues in the leucine zipper domain disrupted dimerization, resulting in this mutant protein not entering the nucleus [44]. In humans, the ß-catenin antagonist Chibby (Cby) forms stable homodimers via its leucine zipper domain. This homodimerization is required for the efficient binding of Chibby to the nucleus import receptor importin-α and subsequence nuclear import [45].

Fifty-four proteins with a Zn(II)_2_Cys_6_-type domain have been identified in *S. cerevisiae* [46], 77 proteins in *Neurospora crassa* [47] and 123 in *Aspergillus nidulans* [48]. Many of these proteins have been implicated in regulating fungal morphogenesis [48]–[50]. In *C. albicans*, *in silico* screening for potential Zn(II)_2_Cys_6_ transcription factors identified 70 potential proteins containing a Zn(II)_2_Cys_6_ motif [51], some of which regulate filamentous growth [34]. In a collection of 143 *C. albicans* mutant strains, each of which has a deletion in a specific transcriptional factor, two strains show an increase in filamentous formation in all of the 55 growth conditions tested [52]. In this sense, in *C. albicans* there are two major repressors of hyphal development: *TUP1* and *NRG1.* Deletion of the *TUP1* gene resulted in cells that are always filamentous [42]. This transcription factor represses the genes responsible for initiating filamentous growth [42], [43]. The C_2_H_2_ zinc finger protein Nrg1p represses hyphal development, and the *ngr1* mutant cells are constitutively filamentous [53]. The *NGR1* transcript level decreases during filamentous growth [54]. According to Lu *et al.* [55], the initiation of hyphal development in *C. albicans* requires a rapid disappearance of Nrg1p in response to the activation of the cAMP-PKA pathway, but the duration of hyphal development is controlled by a reduced occupancy of Nrg1p at hypha-specific gene promoters [55], [56].

Our morphological analysis revealed that the deletion of the *ZNC1* gene resulted in hyperfilamentous colonies and increased the formation of hyphal cells independent of stimuli, growth conditions, or stress, suggesting that *ZNC1* negatively regulates mycelium formation.

The *ZNC1* gene was isolated from a *Y*. *lipolytica* genomic DNA library by functional complementation of the strain. Because the sequence of the *ZNC1* gene in CHY33188 is unaltered, the inability to form hyphae cells in this strain it is not the result of a mutation. However, there must be another mutation in CHY33188 that is suppressed by the introduction of additional copies of the *ZNC1* gene. Until we can identify the mutation that is present in the CHY33188 strain we cannot speculate on the molecular mechanism of the suppression induced by the additional copies of the *ZNC1* gene. The same phenotype was observed in the mutant CHY3350, another smooth non-filamentous strain isolated by chemical mutagenesis of *Y. lipolytica* E122. The gene *YlBMH1* was isolated by its ability to restore hyphal growth upon introduction into CHY3350. As before, the inability to form hyphae cells was not the result of a mutation in the *BMH1* gene [29]. In both cases, the genomic DNA library used was constructed in the autonomously replicating vector pINA445. This plasmid contains the ARS68 sequence; vectors containing this sequence were present at 1–3 copies per cell and were very stable [57]. In both strains, the empty vector, pINA445, has no influence in the phenotype.

As expected for a negative regulator of mycelium formation, *ZNC1* expression is greater in cells exhibiting yeast morphology and is decreased during hyphal cell induction.

Multiple genes, including *ZNC1*, are responsible for the filamentous growth of *Y. lipolytica*. How these genes interact and relate to one another to control dimorphism in *Y. lipolytica* remains unclear. Recently, microarray experiments have identified genes that are potentially involved in the early stages of the dimorphic transition induced by the pH of the medium in *Y. lipolytica*. A total of 61 up-regulated and 165 down-regulated genes were identified. The primary functions of these genes were remodeling and biogenesis of the cell wall, membrane trafficking and glycosylation [58].

We used microarrays to try to identify the genes involved in the hyphal cell formation regulated by Znc1p by comparing the transcriptional profiles of the *znc1::URA3* strain and the parental E122 strain both during exponential growth and at 15, 60 and 180 min during the yeast-to-hypha transition in response to *N*-acetylglucosamine. In total, 1,214 genes showed changes in expression of 2-fold or more between *znc1::URA3* and wild-type cells under the various growth conditions. Most of these genes encode enzymes or proteins involved in metabolic processes, suggesting that Znc1p functions by regulating the distinct responses of *Y. lipolytica* to different growth conditions.

During exponential growth, there are 161 up-regulated genes, and the six most highly up-regulated are as follows: YALI0F13937g, which is highly similar to *C. boidinii* formate dehydrogenase; YALI0E23859g, which is similar to the *S. cerevisiae PHO89* Na+-coupled phosphate transport protein; YALI0C11165g has no similarity; YALI0C08473g, which is similar to the *S. cerevisiae STA1* extracellular alpha-1,4-glucan glucosidase; YALI0B08426g has no similarity and YALI0A12925g, which is similar to the *S. cerevisiae* zinc finger protein *RME1*. Interestingly, in *S. cerevisiae* haploid cells, *RME1* is constitutively expressed at high levels and regulates invasive growth and starch metabolism by modifying the transcription of the *STA2* gen and *MUC1* genes, which encode cell wall-associated proteins required for pseudohyphal formation and invasive growth [59]. In exponential growth, there are 247 down-regulated genes involved in metabolism, with heat shock proteins or with no similarity. The down-regulation of two interesting genes: YALI0B13156g, a calnexin precursor involved in the regulation of secretion, and YALI0D19162g, a Pal3 protein involved in ambient pH sensing, mating and meiosis is notable. However, the up- and down-regulation of these genes are not necessarily linked to Znc1p.

Both analyzing the transcriptional profiles of the *znc1::URA3* strain and the parental E122 strain during the early events of the yeast-to-hypha transition in response to *N*-acetylglucosamine (15, 60 and 180 min) as well as clustering the commonly up-regulated genes did not clearly shows that any particular cell function was increased; however, the gene YALI0C20251g, which is similar to the stress-induced *S. cerevisiae GRE2* gene involved in protection from oxidative damage [38], [60], showed increased expression. The *S. cerevisiae GRE2* gene is induced more than 10-fold during isoamyl alcohol-induced filament formation, and filament formation in *gre2* mutants are derepressed [39]. Similarly, the *C. albicans GRE2* gene is upregulated 2-fold during the induction of hyphal growth with 10% serum [37].

Under this condition, one of the nine down-regulated genes at 15, 60 and 180 min corresponds to YALI0E11517g, a CWP1 cell wall protein specific to the mycelial cells of *Y. lipolytica* [61]. This gene was most over-expressed at 30 min during a mycelium transition induced by a shift of pH of the medium [58].

During the induction of hyphal growth in *C. albicans* with 10% serum, 232 genes exhibited at least a 1.5-fold variation in their expression [37]. Most of the genes showed transient changes in expression, similar to what we observed for *Y. lipolytica*. Therefore, at least in *Y. lipolytica* and *C. albicans*, most of the genes exhibiting variable expression during filamentation are transiently modulated.

However, our global analysis of gene expression did not point to a particular function of Znc1p that readily explains the hyperfilamentous phenotype observed for cells lacking Znc1p.

We therefore selectively focused on the microarray data for the 15 genes that had been previously reported to influence the dimorphic process in *Y. lipolytica,* and performed RT-PCR with 11 of them to validate the microarray experiments. Based on this analysis, we defined three groups of genes. The first group contains genes such as *RAC1*, *ODC1* and *BMH2,* whose expression levels decreased in *znc1::URA3* cells during the yeast-to-hypha transition. An increase in expression of these genes was previously observed in wild-type cells [21], [29], [41], suggesting that Znc1p regulates these genes. The second group contains genes such as *STE7*, *STE11*, *CLA4, BMH1* and *RKA1* whose expression levels differ marginally between *znc1::URA3* cells and wild-type cells, again suggesting a role for Znc1p in the regulation of their expression. The third group contains genes whose expression is unaffected by the deletion of *ZNC1*; these include the *HOY1* and *MHY1* genes that encode known transcription factors [16], [17], [20], the *TPK1* gene that encodes the catalytic subunit of PKA [20], and the *TUP1* gene that also encodes a transcription factor [42], [43].

In *S. cerevisiae*, *C. albicans* and most dimorphic fungi, the dimorphic transition is regulated by the mitogen-activated protein kinase (MAPK) and the cAMP-PKA (PKA) signaling pathways [62]–[65]. In *Y. lipolytica*, the MAPK and PKA pathways have opposing actions in regulating dimorphism [20]. Mutants in the gene *STE11,* which encodes MAPKKK, grow constitutively in the yeast-like form [22], but cells with mutations in the *TPK1* gene, which encodes the PKA catalytic subunit, grow constitutively in the mycelial form [20].

The 14-3-3 proteins *Yl*Bmh1p and *Yl*Bmh2p are closely related to the *S. cerevisiae* proteins Bmh1p and Bmh2p [29]. *Sc*Bmh1p and *Sc*Bmh2p have been found to associate with Ste20p and are required for the RAS/MAPK signaling cascade during pseudohyphal development [5]. *YlBMH1* is involved in the regulation of both hyphal and pseudohyphal growth in *Y. lipolytica*, and the expression levels of *YlBMH1* are increased during the yeast-to-hypha transition [29]. Both *YlBMH1* and *YlBMH2* may be regulated by Znc1p and most likely function in the same MAPK cascade that regulates the hypha-specific genes. This MAPK cascade includes Cla4p (PAK), Ste11p (MAPKKK), and Ste7p (MAPKK), whose genes may also be regulated by Znc1p. Znc1p may also regulate the transcription factor Ste12p, but it remains to be determined if Ste12p and Znc1p converge in this MAPK pathway.

The addition of cAMP inhibited the PKA pathway and the yeast-to-hypha transition in *Y. lipolytica* [66]. The expression of the *RKA1* gene encoding the PKA regulatory subunit is up-regulated in mycelial cells, suggesting that the dimorphic transition is regulated by the PKA pathway via Rka1p [20]. Znc1p may be involved in regulating the dimorphic transition by the PKA pathway via the regulation of the *RKA1* gene by Znc1p.

We propose a working model for the regulation of hyphal growth in *Y. lipolytica* mediated by Znc1p. In our model, the transcription factors Hoy1p and Mhy1p promote the dimorphic transition. Mhy1p acts upstream of Rac1p [21], and Cla4p may act as a direct effector for Rac1p, as has been proposed in *U. maydis* [30]. Acting opposite to Hoy1p and Mhy1p, the transcription factor Tup1p represses the yeast-to-hypha transition. Independent of Tup1, Znc1p also represses the yeast-to-hypha transition, most likely acting through both the MAPK and PKA signaling pathways. Although ours is the first evidence to demonstrate involvement of a signaling pathway mediated by Znc1p in the yeast-to-hypha transition, additional studies are needed to confirm this observation and to implicate other proteins in this process, such as members of the Rho family of proteins and the Ras GTP-binding proteins. Mechanisms, such as the response to oxidative stress, that play a significant role in the differentiation of other fungi are also likely to be important for the yeast-to-hypha transition in *Y. lipolytica* and will need to be integrated into our model to provide a more comprehensive picture of the dimorphic transition in *Y. lipolytica*.

In summary, we have identified a gene, *ZNC1* that encodes a putative transcription factor involved in the dimorphic transition in *Y. lipolytica*. Our morphological examination of cells lacking the *ZNC1* gene, combined with a global analysis of comparative gene expression between the *znc1::URA3* and wild-type strains, shows that Znc1p acts to repress hyphal cell formation and reveals the presence of a complex regulatory network involving distinct, yet interrelated, pathways that function in the regulation of mycelial growth in *Y. lipolytica*.

## Supporting Information

Figure S1 Nucleotide and deduced amino acid sequences of the *ZNC1* gene. The 5′-upstream region contains the following elements: putative TATA box, shown by two continuous lines, CAAT boxes shown by a line of dots and dashes, stress response elements (CCCCT), shown by a dashed line, and a TGACT sequence for Gcn4p binding, shown by a single continuous line. The 3′-downstream sequence contains a typical transcriptional termination motif (TAG…TATGT…TTTT) and a sequence for polyadenylation (TAATAAA), both of which are underlined. The Zn(II)_2_C_6_ fungal-type DNA-binding domain at amino acids 21–51 and the leucine zipper region at amino acids 422–443 are boxed. The bipartite nuclear localization signal at amino acids 16–30 is underlined. The prolines in a proline-rich region at amino acids 94–253 are shadowed. The histidines in a histidine-rich region at amino acids 189–209 are boxed by dots and dashes. The *Spe*I restriction site sequence (ACTAGT) used for GFP tagging is shown in green letters.
(DOCX)

Figure S2 Functional categories of the genes regulated by Znc1p during exponential growth and during hypha formation at 15, 60 and 180 min of incubation in N-acetylglucosamine medium.
(TIF)

Figure S3 Venn diagrams of the transcriptome profiling of *Y. lipolytica* during the dimorphic transition. The numbers in the images represent the number of genes displaying ≥ 2-fold change in expression in *znc1::URA3, Yl*JC35–16 cells relative to wild-type E122 cells at 15, 60 and 180 min of incubation in *N*-acetylglucosamine medium. Colors represent different sections of Venn diagrams.
(TIF)

File S1 Contains Table S1-Table S10.
(PDF)

Files S2 Contains Table S11.
(PDF)

Movie S1 Nuclear dynamics in the *Y. lipolytica*, strain *Yl*AM1AR that expresses Znc1p-GFP as observed by laser scanning confocal microscopy. QuickTime movie. Total time: 2.24 min.
(ZIP)

Movie S2 The synthesis of Znc1p-GFP and its translocation to the nucleus as observed by laser scanning confocal microscopy using strain *Yl*AM1AR *Y*. *lipolytica* cells, expressing Znc1p-GFP. QuickTime movie. Total time: 53.1 min.
(ZIP)
