# Supplementary material for: Identification of the Transcription Factor Znc1p, which Regulates the Yeast-to-Hypha Transition in the Dimorphic Yeast Yarrowia lipolytica
Source: PLoS One. 2013 Jun 24;8(6):e66790. doi: 10.1371/journal.pone.0066790 (PMC3691278; doi:10.1371/journal.pone.0066790)
Supplement: Figure S1 — Nucleotide and deduced amino acid sequences of the ZNC1 gene. The 5′-upstream region contains the following elements: putative TATA box, shown by two continuous lines, CAAT boxes shown by a line of dots and dashes, stress response elements (CCCCT), shown by a dashed line, and a TGACT sequence for Gcn4p binding, shown by a single continuous line. The 3′-downstream sequence contains a typical transcriptional termination motif (TAG…TATGT…TTTT) and a sequence for polyadenylation (TAATAAA), both of which are underlined. The Zn(II)2C6 fungal-type DNA-binding domain at amino acids 21–51 and the leucine zipper region at amino acids 422–443 are boxed. The bipartite nuclear localization signal at amino acids 16–30 is underlined. The prolines in a proline-rich region at amino acids 94–253 are shadowed. The histidines in a histidine-rich region at amino acids 189–209 are boxed by dots and dashes. The SpeI restriction site sequence (ACTAGT) used for GFP tagging is shown in green letters. (DOCX) [file pone.0066790.s001.docx]

-1000 tttgtgttgcatacgacagccgaaaccgtcacaaaagcaaaaagagcattatgtcaccacccctgtgattgcaggctggt

-920 gttggtgcggatgaggaggtgggcgaggaaacatccttgagccgacaccggttgctcacaagggcgggctattgctcctc

-840 tacttttgaggagccttgggacccttctccaccaaacaatatattaccctgctcggttctccacggtttcgtaccaagca

-760 caaaattttacccccttgccccgctctgctgccgtgtttcatttacaagcgagagattctggttgactttttcttttttc

-680 ttttcttttcgttttcgttttcgttttcgttttcgttttctttttttttcgtttctttcctgccgtcgtcgctctcgctt

-600 cgtttttcgacctgcctccgccacccttggtttcgcttctctctgtgctgtgttgcacttttttctgttgtcttttgcgt

-520 tttgtgtttttgtgctttttcccttgccttgtttttgcgattctcgtaacaaagaaacaaaccgctctttgtacacttct

-440 tccaagtcgcatttagcttttcataataccccttgtacgtttgactttgggccttgtgcgttggatacgaaggtaggatc

-360 tgaaggtggggaggatggcgaaacgacgaaatgtggccatagaagaaaaaaaatgtcgaaaccattgtgtgcaccgcgtt

-280 aaggcgagcatccccatgtacttttttgcccgcttggccctctcaaaacccactctttacacacacccccctgtcagctt

-200 aacctacatgcttgtacatgaatatatacttgctacactgctaactctttcttgtcatcatcatcatcacaattgaatag

-120 gagacgagagacacgagccgctaacgagactgcaataccacaagaccgtccacccaacgatatagcggccagcttcaaca

-40 ccacactcaaccttcgtttaaacgtgactctactacacaaatgtcctccaccaaaccacgcaagttccactcacgaaagg

1 M S S T K P R K F H S R K

41 cgcacaagaagtcacacttggggtgtaagacttgcaagagacgacgaattaagtgcgatgagagactgcccagctgctca

14 A H **K K S H L** **G C K T C K R R R I** K **C** D E R L P S **C** S

121 caatgctcgcgcatcgactcaccatgtccatacctagacatgactcctcaagaactgaccttctttcgagaggccaaagt

41 Q **C**  S R I D S P **C** P Y L D M T P Q E L T F F R E A K V

201 caagagcgatgccaccagcttcattaacatgcgcagcatgaaccccacgacccaggtgcccatgcaaggctatggctatc

67 K S D A T S F I N M R S M N P T T Q V P M Q G Y G Y

281 cagctccaccaccgcctcacggaggaccgccaccggcaatgggaatgcaacaaccaatgatgcccatgggctatcctccc

94 **P** A **P P P P** H G G **P P P** A M G M Q Q **P** M M **P** M G Y **P P**

361 caaggggtccctccaccacaaatgcctctgcaacacatgcctcccatggcacccgctcccatggcacaaccgtactatgc

121 Q G V **P P P** Q M **P** L Q H M **P P** M A **P** A **P** M A Q **P** Y Y A

441 aatgcaatacccaccgcccccacagcatatgtatggtcacatgcccatgccaccccagacacagattccacagcaacatc

147 M Q Y **P P P P** Q H M Y G H M **P** M **P P** Q T Q I **P** Q Q H

521 ctgcagcacagccaccacacccgggtcctccaccaccatcaacacatctttctccccatcattcgccacaccagtctcat

174 **P** A A Q **P P** H **P** G **P P P P** S T **H** L S **P** **H H** S **P** **H** Q S **H**

601 cactcaccacactctccgcatgctcacaacgttcccagcgcacccccacctcctaccagtgcgccacaaggcccccactc

201 **H** S **P** **H** S **P** **H** A **H** N V **P** S A **P P P P** T S A **P** Q G **P** H S

681 ttacccaggtgcattctctggctccctgccaagcgttcagaatggcacaacactgccaccgctgggaagctctcctcctt

227 Y **P** G A F S G S L **P** S V Q N G T T L **P P** L G S S **P P**

761 tgagcgccgccggaaactccggagtgaaacttgagaatggacatcaggccgatggtgtgaagacggaaaacgcacagccc

254 L S A A G N S G V K L E N G H Q A D G V K T E N A Q P

841 gtatcgctgcctcccttgacttctacattgcccccgatttccgctatcaccgacagtggtggttctaacgactcgtttca

281 V S L P P L T S T L P P I S A I T D S G G S N D S F Q

921 attgccacctatcatccaagcccacaacccgaaacatgcgactttgccacaaaaatttgggaccggaaacaccgccagca

307 L P P I I Q A H N P K H A T L P Q K F G T G N T A S

1001 ttgtaaagcagggatttgagaccgacatgatgagacacgcttacggtgcatggatctccgacagcatcaagaacgccgag

334 I V K Q G F E T D M M R H A Y G A W I S D S I K N A E

1081 gaccaccctgttttgtaccactctcttttagccttcagccacggctatctgtacctcaagagccactccaaggtccctga

361 D H P V L Y H S L L A F S H G Y L Y L K S H S K V P D

1161 tgagaaagccaacaccggtggcatgacccccgaacaaatccgtgacttgtcgtctcaccaccggtccaaggctctcagca

387 E K A N T G G M T P E Q I R D L S S H H R S K A L S

1241 tgatccatacctacaccgacaatctgggctccaactccgaacagctgccaaacgcttcagatgctcttctggtaaccact

414 M I H T Y T D N **L** G S N S E Q **L** P N A S D A **L** L V T T

1321 ctcattttggcttgggacatatttctgcaggaggacgatatcaagccctatattgagctcagcaagggtctggctgccgt

441 L I **L** A W D I F L Q E D D I K P Y I E L S K G L A A V

1401 tctgcagtccctctctctcaacaccagccagtctcccaccactttctgcatggccgagtcactgtttcagagcatcaagt

467 L Q S L S L N T S Q S P T T F C M A E S L F Q S I K

1481 ccatccatattcctccttacgagtcagggttctggcaggagtttgtgagtaagtttgcttcagtgaagcaccagatcagc

494 S I H I P P Y E S G F W Q E F V S K F A S V K H Q I S

1561 gactccactctgcttctccagtacgccgaggttgaggagtttctcaatgaggtcaccaacatgctcaacaaccaaccccg

521 D S T L L L Q Y A E V E E F L N E V T N M L N N Q P R

1641 aaacttcaacaaccccacatcgtaccctccccagaagctgtatcagttcttgcgacagtggcttacaattttccccagcc

547 N F N N P T S Y P P Q K L Y Q F L R Q W L T I F P S

1721 gagctctgtccggtttccagacgtggaagacccacgacgagaagctgctctactcttactgccacgccgcttctcgagct

574 R A L S G F Q T W K T H D E K L L Y S Y C H A A S R A

1801 ctagatgctctgttccctgaggtgaggttccttttccagattggattcatcgggcctgtggatctggtgggactcgacaa

601 L D A L F P E V R F L F Q I G F I G P V D L V G L D N

1881 ctctctcgaagaaagcctgggacccaacactggctctaccaaccctctgacttaccctctgcgagttgtgggattcttca

627 S L E E S L G P N T G S T N P L T Y P L R V V G F F

1961 agatgcgaacttcgcttgcctctcgtatcttgtttgacgatgacccctttgagggctctgattcgctggtagagcgaaag

654 K M R T S L A S R I L F D D D P F E G S D S L V E R K

2041 aagagcagatttggaatgctcaaggagatatttgtcaactcctttgagaaccccaccgtgccccagttcgaccattacaa

681 K S R F G M L K E I F V N S F E N P T V P Q F D H Y K

2121 gtccaacggcgccaacgaaggtgagagccccgactctgtatccgcctctagtgtcactggtggcagttcgtctcctcgaa

707 S N G A N E G E S P D S V S A S S V T G G S S S P R

2201 cttcggtg**actagt**atcagtggagacgaaggaatggccatggccgcctccactgccaacgccgtgccagctgctgccgga

734 T S V T S I S G D E G M A M A A S T A N A V P A A A G

2281 gcttcttcctggaagcagacagcttttgcccgatatttcgtggatagaatggaaatcttgggcacctagttcggcagagt

761 A S S W K Q T A F A R Y F V D R M E I L G T •

2361 cttgttatagactcgaatatgtatatttaaataacaacaagcaaatatttcgcgggtcgtagctacgcgtatgtaccaag

2441 ccagtacaagtaggaacccgacagttggagagagcaagccacatgataacaatagcgtacaagtacttgtatctggtttc

2521 aagttgctttgttcatgtgcgtctgtgttcgtttaaagacggattgggtaataaagtcccctgagatgtatgtacaagta

2601 tgtattttcgagacagcatcacaaagccatactgtttcaccattgcggagtagctaaattatatccaccggcacaagtat

2681 cgtaccggcgctagtaaaggacacatttgtttatggcctgtctccaccttccccagaacagtcacaaaccatgatgaaca

2761 taccatatcatgttactgaagatcatgatccagagtttgttttgtacagtaatttgcgagtggcctcaggtcatgtacag

2841 tacttgtagcggattgagccgatcaaatcgatacccctgaggcacagatattggccccatctccttgaggtcccccatca

2921 cccctctgaaattaatttttaagcgcacaatgggattgaaattcctcagtaatatctgcattggtaatgtgctacccaaa

3001 tgcagtttttttcccccacggtaacagatttgcatgctgtttattatcattggctgtcgcatttttgcgactggtgcaga

3081 ccgcaattgtgaagccttgcttggtcggacacaaacggttagactggttttggacacatttaccttcgggcttgggggcg

3161 tgtttcgcaaacgcactctaaatgacgtcatgcgagagttgtgacacaagtagctaccactgtacaaagtagtacgctgt

3241 cccgatgcggcaggtttcaaaggatatggatgcaaaatatatccaacaagtgacaatattgggtgcttcgtaaaggcgcc

3321 acagggtgaacttgctggattcccagggtatgagctgaccccccactttttgtgaaaaacccattccccgtttctgttag

3401 aattcaagggaaatggtgagctctcactctttgttgcatctttgatttgcggaaaccctaatcttattatctactcgtag

3481 ttctggcttggttccaccctgttgtctggaaactagcttgctgcagcacactcctatagcaactatgcccgcatgcaacg

3561 tgaccgttgcgtcacccaacagtccccgttggaggttaagttgggctcatggcttgtaataaattgaataaaatcgctaa

3641 aatcgtagaaaatcctgatacaggacatatatcatggtaacaatattttgatgccaaacacgaacctatcccagtttctt

3721 cctcgaggggagctgggggaggaggatgtttcggtcggagttctcccagctctctgttcgtgtcaaagttcgttccacgt

3801 cttcttgcttccttcgctcggcaaccatgttactcattgtggaatgttaaaaaaatgacacacacttgtatttgaaaggt

3881 tgcagaacatttgacgagtcgctaaaaaaaaatgggtagctatatttcacattgtaagagacgataatccactggcgtgc

3961 ttctaattttctcgctgtcaattccccgaaaccggcagtttacccctctttttagtttcataatatccgtattttggtcg

4041 tatttgcgatgcggtgcctcgcgtattttgtctaaactgtgcttgtgcgggaggtccaagcgccaagggtaagggctacc

4121 aaatagggttccgaaagtcgtgctaagggtacgtttataggatacgtaaggatgaatctggcttacgctgccatgagatg

4201 aatatatatgctttctcgtcggtgctggacacgtcggtccgggtttctcaaattcaatttgccaaagaggcgcaatgaac

4281 tctgaacccgcatccgtcacatgaaccttgtaagcatgcagacgctcttatagttgcttgcatatgctgctttgaaacag

4361 gcaaacccaattgttgcatgcgggtggatccatgcggttcctgcgtcaagatgtcgtatttcgtatttgggtgggtcgtc

4441 ctagagtgcaagtggagagcggaaacgatccaca
