# Supplementary figures and images for: Identification of the Transcription Factor Znc1p, which Regulates the Yeast-to-Hypha Transition in the Dimorphic Yeast Yarrowia lipolytica
Source: PLoS One. 2013 Jun 24;8(6):e66790. doi: 10.1371/journal.pone.0066790 (PMC3691278; doi:10.1371/journal.pone.0066790)

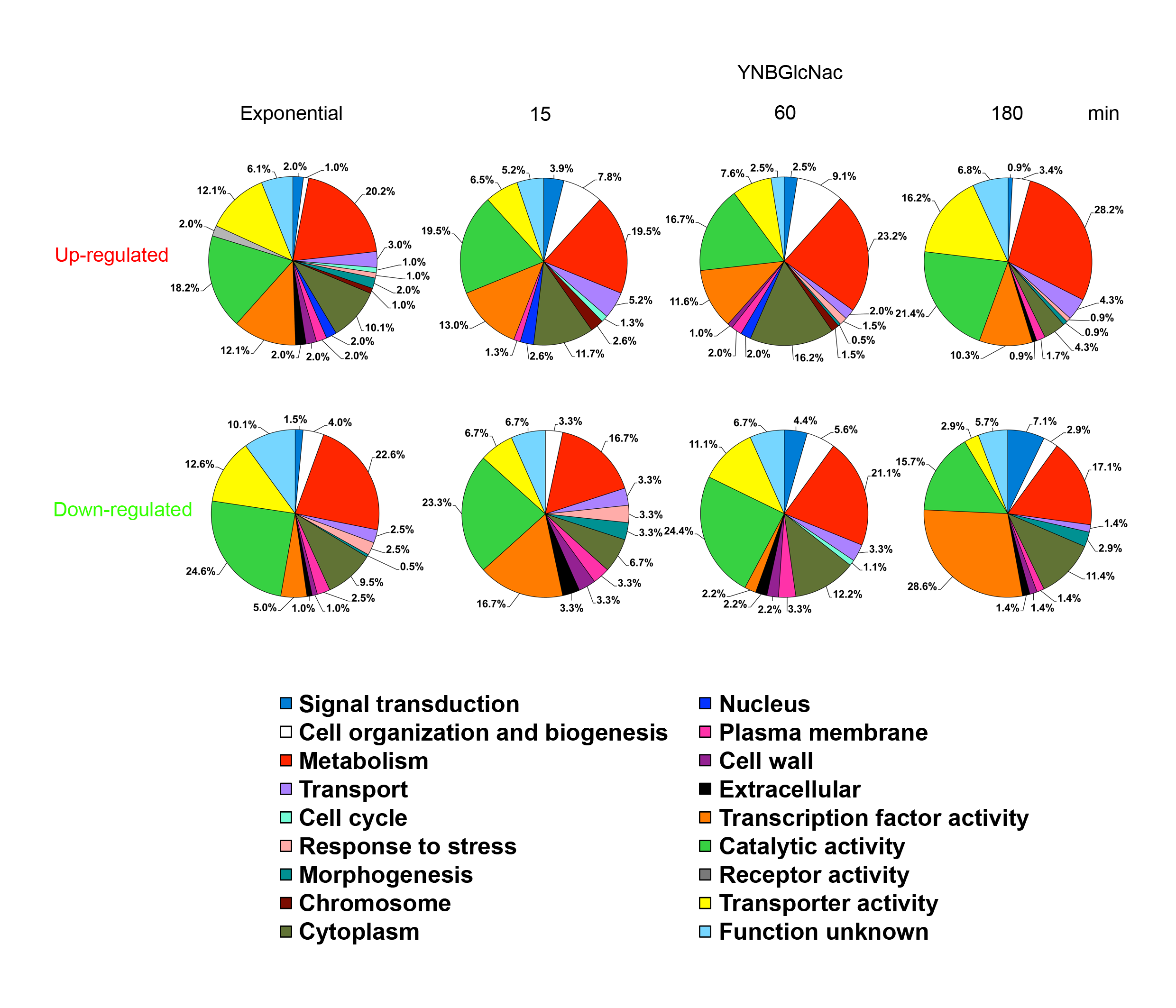

Supplement: Figure S2 — Functional categories of the genes regulated by Znc1p during exponential growth and during hypha formation at 15, 60 and 180 min of incubation in N-acetylglucosamine medium. (TIF) [file pone.0066790.s002.tif]

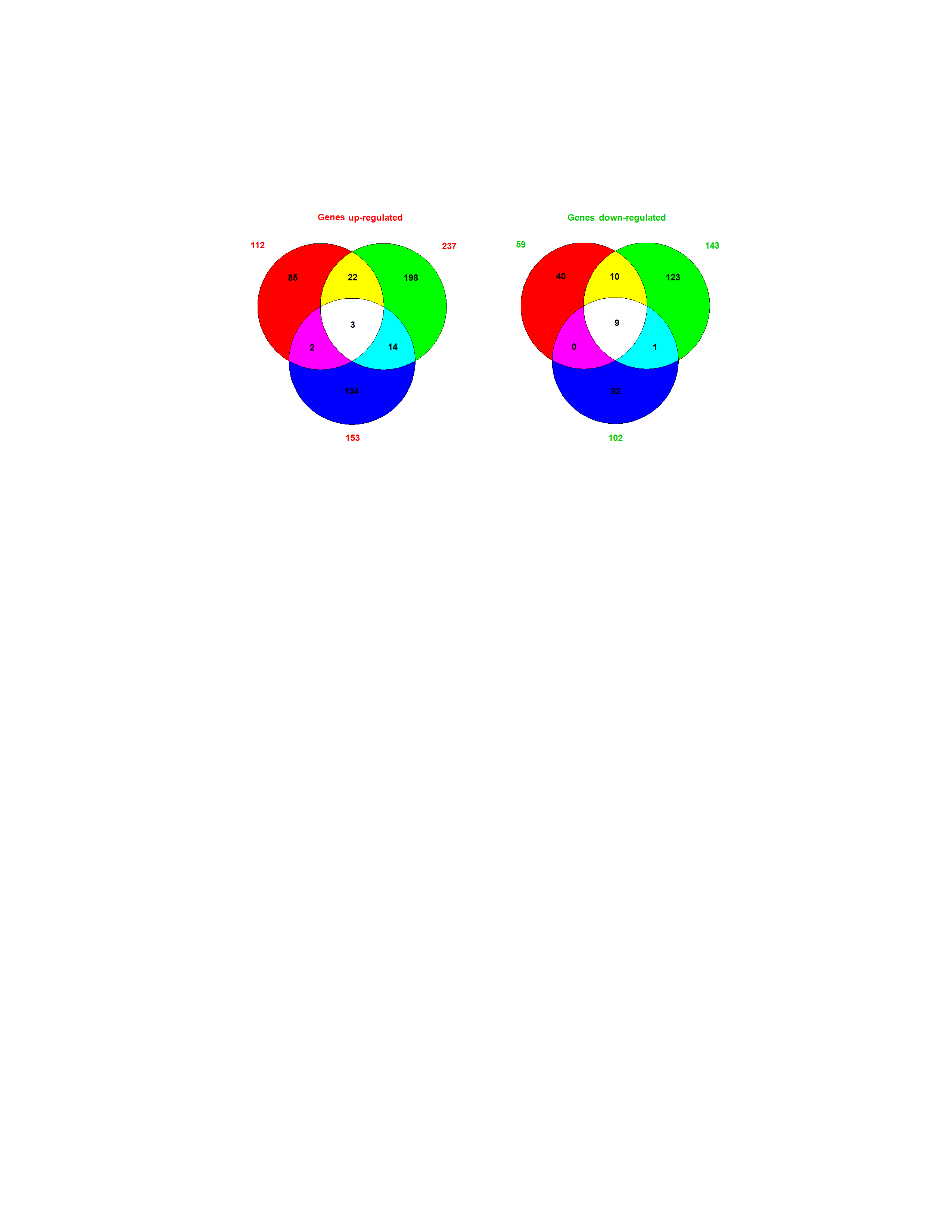

Supplement: Figure S3 — Venn diagrams of the transcriptome profiling of Y. lipolytica during the dimorphic transition. The numbers in the images represent the number of genes displaying ≥ 2-fold change in expression in znc1::URA3, YlJC35–16 cells relative to wild-type E122 cells at 15, 60 and 180 min of incubation in N-acetylglucosamine medium. Colors represent different sections of Venn diagrams. (TIF) [file pone.0066790.s003.tif]
