# Supplementary material for: Identification of the Transcription Factor Znc1p, which Regulates the Yeast-to-Hypha Transition in the Dimorphic Yeast Yarrowia lipolytica
Source: PLoS One. 2013 Jun 24;8(6):e66790. doi: 10.1371/journal.pone.0066790 (PMC3691278; doi:10.1371/journal.pone.0066790)
Supplement: File S1 — Contains Table S1-Table S10. (PDF) [file pone.0066790.s004.pdf]

Table S1. Genes up-regulated (161) during the yeast growth mediated by ZNC1.

Locus tag	Gene	Description	Fold change
YALI0F13937g		highly similar to tr|O93968 Candida boidinii Formate dehydrogenase or tr|O13437 Candida boidinii NAD-dependent formate dehydrogenase, start by similarity	18.25
YALI0E23859g		similar to sp|P38361 Saccharomyces cerevisiae YBR296c PHO89 Na+-coupled phosphate transport protein, high affinity, hypothetical start	18.14
YALI0C11165g		no similarity, hypothetical start	15.68
YALI0C08473g		weakly similar to sp|P08640 Saccharomyces cerevisiae YIR019c STA1 extracellular alpha-1, 4-glucan glucosidase, hypothetical start	13.35
YALI0B08426g		no similarity, hypothetical start	8.64
YALI0A12925g		weakly similar to sp|P32338 Saccharomyces cerevisiae Zinc finger protein RME1, hypothetical start	6.61
YALI0B09867g		no similarity, hypothetical start	6.20
YALI0E31603g		similar to KLLA0B14839g Kluyveromyces lactis IPF 6866.1, hypothetical start	6.12
YALI0F09691g		similar to tr|Q06593 Saccharomyces cerevisiae Similar to S. CEREVISIAE hypothetical protein HRD799 and tr|Q06593 Saccharomyces cerevisiae Similar to S. CEREVISIAE hypothetical protein HRD799, hypothetical start	5.50
YALI0F10373g		some similarities with sp|P18899 Saccharomyces cerevisiae YMR173w DDR48 heat shock protein, hypothetical start	5.48
YALI0C23452g		no similarity, hypothetical start	5.46
YALI0D19602g		similar to DEHA0E01276g Debaryomyces hansenii IPF 5727.1, hypothetical start	5.45
YALI0C23474g		no similarity, hypothetical start	5.24
YALI0D24145g		weakly similar to tr|Q08991 Saccharomyces cerevisiae YPL279C, hypothetical start	4.91
YALI0E32329g		some similarities with tr|Q95XU8 Caenorhabditis elegans, hypothetical start	4.61
YALI0E03938g		no similarity, hypothetical start	4.42
YALI0C03564g		no similarity, hypothetical start	4.00
YALI0D06017g		similar to sp|P40350 Saccharomyces cerevisiae YPL227c ALG5 Dolichyl-phosphate beta-glucosyltransferase, hypothetical start	3.99
YALI0E22286g		highly similar to tr|P78977 Yarrowia lipolytica Cell wall protein precursor YlCWP1 (strain cx39-74a), start by similarity	3.97
YALI0C09031g		no similarity, hypothetical start	3.94
YALI0E01364g		similar to tr|Q9HFS3 Pneumocystis carinii Guanine nucleotide-binding protein beta subunit, hypothetical start	3.87
YALI0F21637g		weakly similar to sp|Q12495 Saccharomyces cerevisiae YPR018w RLF2 chromatin assembly complex, subunit p90, hypothetical start	3.80
YALI0E03036g		similar to sp|P55059 Humicola insolens Protein disulfide isomerase precursor, hypothetical start	3.78

YALI0C19360g		no similarity, hypothetical start	3.71
YALI0E03366g		similar to sp|P38332 Saccharomyces cerevisiae YBR246w, start by similarity	3.70
YALI0E18788g	YlCWP1	tr|Q8TFK5 Yarrowia lipolytica Cell wall protein, identified start	3.69
YALI0B19976g		highly similar to tr|O93968 Candida boidinii Formate dehydrogenase (EC 1.2.1.2), start by similarity	3.67
YALI0E08756g		similar to DEHA0F10351g Debaryomyces hansenii, no start	3.64
YALI0E06039g		similar to sp|P48240 Saccharomyces cerevisiae YGR158c MTR3 involved in mRNA transport and DEHA0A13343g Debaryomyces hansenii, start by similarity	3.59
YALI0E27654g	YlPOX4	tr|O74937 Yarrowia lipolytica Acyl-CoA oxidase 4 (EC 1.3.3.6), peroxisomal, identified start	3.56
YALI0E01584g		similar to DEHA0E11000g Debaryomyces hansenii, hypothetical start	3.54
YALI0F05016g		no similarity, hypothetical start	3.53
YALI0B19602g		similar to tr|Q9Y7W9 Yarrowia lipolytica Mycelial growth factor-1, start by similarity	3.52
YALI0B19008g		similar to tr|Q9C101 Schizosaccharomyces pombe Putative membrane transporter, hypothetical start	3.51
YALI0E06809g		some similarities with tr|Q12057 Saccharomyces cerevisiae YOR104w, hypothetical start	3.50
YALI0F03685g		no similarity, hypothetical start	3.47
YALI0F15543g		no similarity, hypothetical start	3.43
YALI0E24827g		weakly similar to DEHA0E12430g Debaryomyces hansenii, hypothetical start	3.24
YALI0A11473g		similar to tr|Q8X1F2 Venturia inaequalis ATP-binding cassette transporter ABC4, start by similarity	3.23
YALI0F21659g		similar to tr|Q12436 Saccharomyces cerevisiae ORF YLR130C, start by similarity	3.20
YALI0B07623g		some similarities with sp|P53705 Candida albicans Integrin alpha chain-like protein (Alpha-INT1), hypothetical start	3.20
YALI0E32835g	YlPOX1	tr|O74934 Yarrowia lipolytica Acyl-CoA oxidase 1 (EC 1.3.3.6), peroxisomal, identified start	3.19
YALI0D09185g		some similarities with sp|P32323 Saccharomyces cerevisiae YNR044w AGA1 a-agglutinin anchor subunit, hypothetical start	3.15
YALI0A15488g	YlALK7	tr|O74133 Yarrowia lipolytica ALK7 cytochrome P450, identified start	3.15
YALI0E10747g		similar to DEHA0F17336g Debaryomyces hansenii, hypothetical start	3.10
YALI0F10901g		some similarities with sp|P08640 Saccharomyces cerevisiae YIR019c, hypothetical start	3.08
YALI0F02959g		similar to sp|P38227 Saccharomyces cerevisiae YBR043c, start by similarity	3.08
YALI0D23705g		similar to sp|P15303 Saccharomyces cerevisiae YPR181c SEC23 component of COPII coat of ER-golgi vesicles, start by similarity	3.04
YALI0A18271g		no similarity, hypothetical start	3.04
YALI0B11440g		similar to sp|O14199 Schizosaccharomyces pombe Hypothetical protein C5D6.06c in chromosome I, hypothetical start	3.02
YALI0D26598g		weakly similar to sp|P40963 Saccharomyces cerevisiae SAS2 protein, hypothetical start	2.97

YALI0A02178g	similar to DEHA0G23056g Debaryomyces hansenii IPF 4911.1, hypothetical start	2.97
YALI0D06369g	no similarity, hypothetical start	2.95
YALI0E02882g	similar to DEHA0G25817g Debaryomyces hansenii and sp|P45976 Saccharomyces cerevisiae YJR093c FIP1 component of pre-mRNA polyadenylation factor PF I, possible transmembrane segment, start by similarity	2.93
YALI0C10945g	similar to CA0697|CaVTC4 Candida albicans CaVTC4 putative polyphosphate synthetase, start by similarity	2.89
YALI0D10967g	similar to sp|Q92389 Yarrowia lipolytica Acid extracellular protease precursor (EC 3.4.23.-), start by similarity	2.88
YALI0B09669g	similar to tr|Q96V37 Pichia pastoris Ceramide glucosyltransferase, hypothetical start	2.87
YALI0F08833g	weakly similar to sp|P15424 Saccharomyces cerevisiae YDR194c MSS116 RNA helicase of the DEAD box family, hypothetical start	2.87
YALI0E07271g	similar to sp|P32528 Saccharomyces cerevisiae YBR208c DUR1_2 urea amidolyase, hypothetical start	2.86
YALI0F03113g	some similarities with sp|P32581 Saccharomyces cerevisiae YJL106w IME2 ser/thr protein kinase, hypothetical start	2.85
YALI0E14971g	no similarity, hypothetical start	2.84
YALI0C19668g	similar to sp|Q12335 Saccharomyces cerevisiae YDR032c PST2, start by similarity	2.84
YALI0E15686g	weakly similar to sp|P37267 Saccharomyces cerevisiae YGR174c CBP4 ubiquinol--cytochrome-c reductase assembly factor, hypothetical start	2.83
YALI0E31218g	no similarity	2.83
YALI0A20790g	no similarity, hypothetical start	2.81
YALI0F28061g	similar to tr|Q07015 Saccharomyces cerevisiae YDR257c RMS1 transcriptional regulator, start by similarity	2.79
YALI0E18161g	no similarity, frameshift	2.79
YALI0C23925g	weakly similar to sp|P47149 Saccharomyces cerevisiae YJR112w NNF1 nuclear envelope protein, hypothetical start	2.76
YALI0B21296g	no similarity, hypothetical start	2.75
YALI0B10219g	some similarities with CAGL0L04114g Candida glabrata and sp|P53919 Saccharomyces cerevisiae Hypothetical 54.9 kDa protein in SPC98-TOM70 intergenic region, hypothetical start	2.74
YALI0C15268g	no similarity, hypothetical start	2.72
YALI0B18854g	weakly similar to sp|Q03649 Saccharomyces cerevisiae YMR210w similarity to P.glauca late embryogenesis abundant protein and YBR177c and YPL095c P3. 17.f3.1, hypothetical start	2.69
YALI0D06479g	no similarity, hypothetical start	2.66
YALI0D10329g	weakly similar to tr|O42662 Schizosaccharomyces pombe Possible DNA methylase, hypothetical start	2.65

YALI0F25839g	similar to sp|P10281 Aspergillus oryzae ribonuclease T2 precursor and tr|Q02933 Saccharomyces cerevisiae YPL123c RNY1 ribonuclease T2, hypothetical start	2.64
YALI0C08833g	no similarity	2.62
YALI0C19426g	similar to sp|Q12381 Schizosaccharomyces pombe Pre-mRNA splicing factor prp1, start by similarity	2.62
YALI0F25245g	no similarity	2.60
YALI0E03498g	weakly similar to DEHA0D15796g Debaryomyces hansenii, hypothetical start	2.59
YALI0C04818g	some similarities with sp|P34248 Saccharomyces cerevisiae YKL100c similarity to C.elegans hypothetical protein, hypothetical start	2.59
YALI0E09218g	no similarity, hypothetical start	2.55
YALI0A20834g	weakly similar to sp|Q02959 Saccharomyces cerevisiae HOS3 protein, hypothetical start	2.51
YALI0E27203g	similar to sp|P41948 Saccharomyces cerevisiae YNL142w MEP2, hypothetical start	2.51
YALI0F03025g	weakly similar to tr|CAD70893 Neurospora crassa 100H1.080 gene, hypothetical start	2.51
YALI0E29953g	weakly similar to DEHA0C18260g Debaryomyces hansenii IPF 3283.1, hypothetical start	2.49
YALI0B13882g	similar to tr|Q96UB2 Neurospora crassa Conserved hypothetical protein, start by similarity	2.48
YALI0E35222g	similar to sp|P08540 Kluyveromyces lactis Potential acid phosphatase (EC 3.1.3.2), hypothetical start	2.48
YALI0F21945g	similar to sp|P30666 Schizosaccharomyces pombe DNA replication licensing factor mcm3 (Minichromosome maintenance protein 3) and sp|P24279 Saccharomyces cerevisiae YEL032w MCM3 replication initiation protein, hypothetical start	2.48
YALI0E11627g	similar to sp|P28868 Candida albicans CAG1 Guanine nucleotide-binding protein alpha subunit, hypothetical start	2.45
YALI0E03388g	similar to sp|Q09196 Schizosaccharomyces pombe Myosin regulatory light chain cdc4, start by similarity	2.43
YALI0D08635g	highly similar to sp|P35179 Saccharomyces cerevisiae YDR086c SSS1 ER protein-translocase complex subunit, start by similarity	2.43
YALI0C03586g	no similarity, hypothetical start	2.41
YALI0B10802g	similar to tr|Q8X005 Neurospora crassa Glycine rich protein (het-COR), hypothetical start	2.40
YALI0D25828g	similar to sp|Q08234 Saccharomyces cerevisiae Probable ATP-dependent transporter YOL074C/YOL075C, hypothetical start	2.40
YALI0F30063g	similar to sp|P47082 Saccharomyces cerevisiae YJR001w AVT1 neutral amino acid transporter, hypothetical start	2.39
YALI0D25872g	similar to sp|P32867 Saccharomyces cerevisiae SSO1 protein, hypothetical start	2.37
YALI0B11880g	highly similar to tr|Q08235 Saccharomyces cerevisiae Chromosome XV reading frame ORF YOL077C, start by similarity	2.37
YALI0E06519g	weakly similar to sp|P34244 Saccharomyces cerevisiae YKL101w HSL1 ser/thr protein kinase, coupling septin ring assembly to cell cycle progression, hypothetical start	2.35
YALI0F26081g	no similarity, hypothetical start	2.35

YALI0B04884g	similar to tr|Q873I9 Neurospora crassa B9B11.100 Related to pirin, hypothetical start	2.34
YALI0F30569g	similar to sp|P17064 Saccharomyces cerevisiae YER056c FCY2 purine-cytosine permease, hypothetical start	2.34
YALI0D06545g	similar to sp|P18414 Saccharomyces cerevisiae YBL040c ERD2 ER lumen protein-retaining HDEL receptor, start by similarity	2.33
YALI0D25938g	similar to sp|P08004 Saccharomyces cerevisiae YNL192w CHS1 chitin synthase I P2.162.f2.1, hypothetical start	2.33
YALI0B22550g	similar to tr|Q9VM58 Drosophila melanogaster CG10399 protein, start by similarity	2.30
YALI0F15411g	similar to CA4398|CaZRT1 Candida albicans CaZRT1, hypothetical start	2.30
YALI0B07601g	weakly similar to tr|Q06991 Saccharomyces cerevisiae SIMILARITY to hypothetical 81 kDa protein in CNB1-ORD1 intergenic region, hypothetical start	2.29
YALI0E21846g	similar to sp|P09032 Saccharomyces cerevisiae YOR260w GCD1 translation initiation factor eIF2bgamma subunit, hypothetical start	2.29
YALI0F23463g	similar to sp|P47143 Saccharomyces cerevisiae Putative adenosine kinase (EC 2.7.1.20) YJR105w ADO1, start by similarity	2.28
YALI0C01617g	some similarities with sp|P42951 Saccharomyces cerevisiae YJL100w, hypothetical start	2.28
YALI0F14025g	weakly similar to tr|Q12494 Saccharomyces cerevisiae YDR017c KCS1 potential transcription factor of the BZIP type, hypothetical start	2.27
YALI0A20614g	similar to tr|Q06506 Saccharomyces cerevisiae of the beta-transducin family of guanine nucleotide-binding, hypothetical start	2.27
YALI0C18183g	highly similar to DEHA0G23144g Debaryomyces hansenii IPF 4906.1, hypothetical start	2.26
YALI0D01936g	weakly similar to sp|P53938 Saccharomyces cerevisiae YNL080c hypothetical protein, start by similarity	2.26
YALI0E07920g	similar to DEHA0B03696g Debaryomyces hansenii, hypothetical start	2.25
YALI0A03399g	no similarity, hypothetical start	2.25
YALI0B09713g	weakly similar to sp|P07272 Saccharomyces cerevisiae Pyrimidine pathway regulatory protein 1, hypothetical start	2.25
YALI0C19844g	no similarity, hypothetical start	2.25
YALI0F21428g	some similarities with sp|P10667 Xenopus laevis Integumentary mucin A.1 precursor (FIM-A.1) (Preprospasmolysin), hypothetical start	2.25
YALI0E24673g	weakly similar to sp|P53301 Saccharomyces cerevisiae YGR189c CRH1, hypothetical start	2.24
YALI0C02409g	similar to sp|P39719 Saccharomyces cerevisiae Hypothetical 87.5 kDa protein in ACS1-GCV3 intergenic region, start by similarity	2.24
YALI0E09449g	similar to ca|CA5611|CaSUA5 Candida albicans CaSUA5, hypothetical start	2.24
YALI0B20086g	weakly similar to sp|P10662 Saccharomyces cerevisiae Mitochondrial 40S ribosomal protein MRP1, hypothetical start	2.24
YALI0E22957g	similar to DEHA0F02662g Debaryomyces hansenii, hypothetical start	2.23

YALI0E28548g	no similarity, hypothetical start	2.23
YALI0B12606g	no similarity, hypothetical start	2.22
YALI0F03905g	some similarities with sp|P06842 Saccharomyces cerevisiae YFL026w STE2 pheromone alpha-factor receptor, hypothetical start	2.22
YALI0F12595g	weakly similar to tr|CAD70921 Neurospora crassa 7F4. 170 Related to acid sphingomyelinase, hypothetical start	2.21
YALI0F19316g	no similarity, hypothetical start	2.21
YALI0B21032g	similar to tr|Q8C128 Mus musculus Splicing factor 3 subunit 1, start by similarity	2.20
YALI0A01023g	similar to sp|Q12691 Saccharomyces cerevisiae YDR038c ENA5 P-type ATPase involved in Na+ efflux, start by similarity	2.20
YALI0F24123g	highly similar to sp|P47913 Saccharomyces cerevisiae YOR312c RPL18B 60S large subunit ribosomal protein or sp|P47913 Saccharomyces cerevisiae YMR242c RPL18A 60s large subunit ribosomal protein, start by similarity	2.20
YALI0E01034g	some similarities with sp|Q09174 Schizosaccharomyces pombe SPCC736.04C Alpha-1, 2-galactosyltransferase, possible transmembrane segment, hypothetical start	2.20
YALI0F20900g	weakly similar to sp|Q9HDU6 Schizosaccharomyces pombe Probable 2-dehydropantoate 2-reductase (EC 1.1.1. 169) (Ketopantoate reductase), hypothetical start	2.19
YALI0B13046g	weakly similar to sp|P36033 Saccharomyces cerevisiae YKL220c FRE2 ferric (and cupric) reductase P9.3. f7.1 or sp|Q08905 Saccharomyces cerevisiae YOR381w FRE3 or sp|P53746 Saccharomyces cerevisiae YNR060w FRE4, start by similarity	2.18
YALI0E33583g	similar to sp|P33413 Saccharomyces cerevisiae YHL016c DUR3 urea transport protein singleton, start by similarity	2.18
YALI0B05434g	no similarity, hypothetical start	2.18
YALI0A10252g	weakly similar to sp|P38803 Saccharomyces cerevisiae YHR085w of the Brahma transcriptional activator, start by similarity	2.18
YALI0B06919g	no similarity, hypothetical start	2.18
YALI0B23408g	highly similar to sp|P40046 Saccharomyces cerevisiae YER072w VTC1 Negative Regulator of Cdc Fourty two (CDC42) singleton	2.17
YALI0C09141g	sp|O59949 Yarrowia lipolytica Elongation factor 1-alpha (EF-1-alpha), identified start	2.17
YALI0F09515g	no similarity	2.17
YALI0C00693g	highly similar to gi|7839181|sgd|S0007455 Saccharomyces cerevisiae YHR072wa NOP10 nucleolar rRNA processing protein, start by similarity	2.17
YALI0C10923g	no similarity	2.16
YALI0D04763g	no similarity, hypothetical start	2.16
YALI0F24365g	similar to KLLA-IPF1520.1 Kluyveromyces lactis, hypothetical start	2.16
YALI0C08701g	weakly similar to tr|O94395 Schizosaccharomyces pombe Putative ATP-dependent DNA helicase, start by similarity	2.15
YALI0C06754g	weakly similar to tr|Q96U38 Neurospora crassa Conserved hypothetical protein, start by	2.14

	similarity	
YALI0F27907g	similar to sp|P43601 Saccharomyces cerevisiae YFR021w unknown function, start by similarity	2.14
YALI0E03542g	no similarity, hypothetical start	2.13
YALI0D17798g	no similarity, hypothetical start	2.13
YALI0C23430g	no similarity, hypothetical start	2.12
YALI0E22220g	similar to sp|P54837 Saccharomyces cerevisiae YML012w ERV25 component of the COPII-coated vesicles 25 Kda, start by similarity	2.12
YALI0A19228g	similar to tr|Q9P7F3 Schizosaccharomyces pombe Ammonium transporter, hypothetical start	2.11
YALI0E14366g	similar to tr|Q9Y763 Phanerochaete chrysosporium 1, 4-benzoquinone reductase, start by similarity	2.10
YALI0E30965g	similar to sp|P32316 Saccharomyces cerevisiae ACH1 Acetyl-CoA hydrolase (EC 3.1.2.1) (Acetyl-CoA deacylase) (Acetyl-CoA acylase), hypothetical start	2.09
YALI0D10043g	similar to tr|Q86ZH9 Neurospora crassa 64C2.200 putative tartrate transporter, hypothetical start	2.09
YALI0D15906g	similar to tr|Q9P8F7 Yarrowia lipolytica Triacylglycerol lipase precursor, start by similarity	2.08
YALI0F03751g	weakly similar to sp|P40474 Saccharomyces cerevisiae YIL121w similarity to antibiotic resistance proteins, hypothetical start	2.08
YALI0D09339g	weakly similar to tr|O74251 Emericella nidulans Medusa transcriptional regulator, hypothetical start	2.07
YALI0C14344g	highly similar to tr|O93968 Candida boidinii Formate dehydrogenase (EC 1.2.1.2), start by similarity	2.06
YALI0C05082g	similar to sp|P40525 Saccharomyces cerevisiae YIL052c RPL34B ribosomal protein L34.e, hypothetical start	2.05

Table S2. Genes down-regulated (247) during the yeast growth mediated by ZNC1.

Locus tag	Gene	Description	Fold change
YALI0E20625g		similar to tr|Q9C2Y5 Aspergillus awamori Uracil phosphoribosyltransferase, hypothetical start	0.50
YALI0E20471g		similar to tr|CAD70735 Neurospora crassa 64C2.200 Related to putative tartrate transporter, hypothetical start	0.50
YALI0F27621g		no similarity, hypothetical start	0.50
YALI0C07953g		highly similar to sp|P46598 Candida albicans Heat shock protein 90 homolog, start by similarity	0.49
YALI0F16753g		weakly similar to tr|Q9XYW6 Drosophila melanogaster CHIP protein (RE01069p), hypothetical start	0.49
YALI0E23474g	YlALK3	tr|O74129 Yarrowia lipolytica, identified start	0.49
YALI0B23122g		similar to sp|O13725 Schizosaccharomyces pombe Hypothetical protein C15A10.05c in chromosome I, hypothetical start	0.49
YALI0E31889g		weakly similar to tr|Q8EL71 Oceanobacillus iheyensis Hypothetical conserved protein, hypothetical start	0.49
YALI0E24695g		similar to DEHA0F09559g Debaryomyces hansenii, start by similarity	0.48
YALI0D21593g		similar to tr|Q88EF6 Pseudomonas putida (strain KT2440) Hypothetical protein, start by similarity	0.48
YALI0C22748g		similar to tr|Q9WXD6 Brevibacterium linens 1, 4-butanediol diacrylate esterase, hypothetical start	0.48
YALI0F18590g		similar to sp|P14065 Saccharomyces cerevisiae YOR120w GCY1 galactose-induced protein of aldo/keto reductase or sp|Q12458 Saccharomyces cerevisiae YDR368w YPR1 strong similarity to members of the aldo/keto reductase family, start by similarity	0.48
YALI0E23408g		similar to DEHA0B05071g Debaryomyces hansenii, hypothetical start	0.48
YALI0C07612g		no similarity, hypothetical start	0.48
YALI0E20207g		weakly similar to tr|Q9C0M1 Candida albicans CaNAG5 protein, hypothetical start	0.48
YALI0B15840g		similar to tr|Q12449 Saccharomyces cerevisiae Hypothetical 39.4 kDa protein, start by similarity	0.47
YALI0E34045g		similar to tr|Q8X8M5 Escherichia coli Orf, hypothetical protein, hypothetical start	0.47
YALI0C10098g		similar to tr|Q9VVR8 Drosophila melanogaster CG4108 protein or tr|Q95SH2 Drosophila melanogaster GH26351p	0.47
YALI0F07953g		no similarity	0.47
YALI0C11209g		no similarity, hypothetical start	0.47
YALI0E11209g		no similarity, hypothetical start	0.47
YALI0B16852g		similar to DEHA0B12474g Debaryomyces hansenii IPF 10115.1, hypothetical start	0.47
YALI0E33935g		weakly similar to KLLA0F16709g Kluyveromyces lactis, hypothetical start	0.47

YALI0F30987g		similar to tr|Q9HDP7 Emericella nidulans Catalase, start by similarity	0.47
YALI0D22352g		highly similar to sp|P41797 Candida albicans Heat shock protein SSA1, start by similarity	0.46
YALI0C23859g	YlPOX5	tr|O74938 Yarrowia lipolytica Acyl-CoA oxidase 5 (EC 1.3.3.6), peroxisomal, identified start	0.46
YALI0E25828g		no similarity, possibly noncoding, hypothetical start	0.46
YALI0C06171g		similar to tr|Q884Q9 Pseudomonas syringae Oxidoreductase zinc-binding, hypothetical start	0.46
YALI0C20405g		similar to sp|P10768 Homo sapiens Esterase D (EC 3. 1.1.1), hypothetical start	0.46
YALI0D08206g		similar to sp|Q08108 Saccharomyces cerevisiae YOL011w PLB3 phospholipase B (lysophospholipase), hypothetical start	0.46
YALI0E02684g		highly similar to sp|P00890 Saccharomyces cerevisiae YNR001c CIT1 citrate (si)-synthase, mitochondrial, possible transmembrane segment, hypothetical start	0.46
YALI0D05379g		similar to sp|P38928 Saccharomyces cerevisiae YIL140w SRO4 required for axial pattern of budding, hypothetical start	0.46
YALI0A11847g		no similarity, hypothetical start	0.46
YALI0B19228g		no similarity, hypothetical start	0.46
YALI0B07403g		some similarities with sp|P38195 Saccharomyces cerevisiae YBL043w ECM13 involved in cell wall structure of biosynthesis, start by similarity	0.46
YALI0B09955g		no similarity, hypothetical start	0.46
YALI0E34375g		similar to wi|NCU00108.1 Neurospora crassa NCU00108. 1 hypothetical protein (93222 - 91750), hypothetical start	0.46
YALI0F27049g		no similarity	0.45
YALI0A10901g		no similarity	0.45
YALI0F23595g		similar to wi|NCU08303.1 Neurospora crassa NCU08303. 1 hypothetical protein (49528 - 52671), hypothetical start	0.45
YALI0E08382g		no similarity, hypothetical start	0.45
YALI0D00649g		similar to sp|P32317 Saccharomyces cerevisiae YEL052w AFG1 ATPase family gene, start by similarity	0.45
YALI0E31757g		weakly similar to tr|Q8X0W7 Neurospora crassa 123A4. 250 Related to NsdD protein, hypothetical start	0.45
YALI0E25091g		similar to DEHA0G19030g Debaryomyces hansenii, hypothetical start	0.45
YALI0F12551g		similar to sp|P35191 Saccharomyces cerevisiae YFL016c, start by similarity	0.45
YALI0B21406g		similar to DEHA0F08206g Debaryomyces hansenii IPF 8485.1, start by similarity	0.45
YALI0E25047g		no similarity, hypothetical start	0.44
YALI0A17314g		similar to tr|Q9P3X9 Neurospora crassa Peptidyl-prolyl cis-trans isomerase, hypothetical start	0.44
YALI0F00880g		similar to sp|P25491 Saccharomyces cerevisiae YNL064c YDJ1 mitochondrial and ER import protein, start by similarity	0.44

YALI0E06083g		similar to sp|P53285 Saccharomyces cerevisiae YGR141w, hypothetical start	0.44
YALI0E03674g		similar to sp|P43535 Saccharomyces cerevisiae YFR009w GCN20 positive effector of GCN2P, start by similarity	0.44
YALI0E03212g		similar to sp|P32891 Saccharomyces cerevisiae YDL174c DLD1 D-lactate ferricytochrome C oxidoreductase (D-LCR), possible transmembrane segment, hypothetical start	0.44
YALI0A03069g		no similarity, possibly noncoding, hypothetical start	0.44
YALI0D16445g		weakly similar to tr|O94461 Schizosaccharomyces pombe Putative phosphoglycerate mutase, start by similarity	0.44
YALI0D25630g	YlADH1	tr|Q9UW08 Yarrowia lipolytica Alcohol dehydrogenase 1, identified start	0.44
YALI0E23947g		similar to sp|P07703 Saccharomyces cerevisiae YPR110c RPC40 DNA-directed RNA polymerase I and III 40 kDa polypeptide, hypothetical start	0.44
YALI0D05687g		similar to tr|Q87R47 Vibrio parahaemolyticus Methylated-DNA-protein-cysteine methyltransferase-related protein, start by similarity	0.43
YALI0A01089g		no similarity, hypothetical start	0.43
YALI0C00297g		no similarity, hypothetical start	0.43
YALI0D07436g		similar to sp|P38431 Saccharomyces cerevisiae YPR041w TIF5 translation initiation factor eIF5, start by similarity	0.43
YALI0E20515g		weakly similar to DEHA0G10219g Debaryomyces hansenii, hypothetical start	0.43
YALI0A12815g		no similarity, hypothetical start	0.43
YALI0A10747g		weakly similar to CAGL0H02101g Candida glabrata, start by similarity	0.43
YALI0C03465g		similar to sp|P15992 Saccharomyces cerevisiae YBR072w HSP26 heat shock protein, hypothetical start	0.43
YALI0C14388g		similar to tr|O74529 Schizosaccharomyces pombe Probable methyltransferase, start by similarity	0.43
YALI0A19910g		similar to sp|P47137 Saccharomyces cerevisiae Probable oxidoreductase YJR096W, hypothetical start	0.42
YALI0D17138g		similar to sp|Q01662 Saccharomyces cerevisiae YLR244c MAP1 methionine aminopeptidase, isoform 1, start by similarity	0.42
YALI0F09559g		similar to sp|P22543 Saccharomyces cerevisiae YLR240w VPS34 phosphatidylinositol 3-kinase and DEHA0G14157g Debaryomyces hansenii, no start	0.42
YALI0B17798g		similar to tr|Q8X179 Emericella nidulans Cytoplasmic dynein intermediate chain, start by similarity	0.42
YALI0F26983g		similar to CA0775|CaIFK2 Candida albicans CaIFK2 probable monoxygenase, hypothetical start	0.42
YALI0E24211g		similar to CAGL0J08613g Candida glabrata, hypothetical start	0.42
YALI0B05918g		similar to sp|P38705 Saccharomyces cerevisiae Putative seryl-tRNA synthetase YHR011W (EC 6.1.1.11), hypothetical start	0.42

YALI0B03366g	similar to sp|P14306 Saccharomyces cerevisiae DKA1 protein, hypothetical start	0.42
YALI0E05511g	no similarity, hypothetical start	0.41
YALI0B05236g	no similarity, hypothetical start	0.41
YALI0C09240g	some similarities with sp|Q86JZ0|emb|AAO51355 Dictyostelium discoideum Hypothetical protein, hypothetical start	0.41
YALI0D06303g	highly similar to sp|P28241 Saccharomyces cerevisiae YOR136w IDH2 isocitrate dehydrogenase, hypothetical start	0.41
YALI0E18744g	no similarity, hypothetical start	0.41
YALI0B03124g	similar to tr|Q8NKC1 Schizosaccharomyces pombe Putative O-methyltransferase, possibly involved in homocysteine metabolism, hypothetical start	0.41
YALI0D11242g	similar to sp|P47077 Saccharomyces cerevisiae Hypothetical 77.7 kDa protein in CCT3-CCT8 intergenic region, start by similarity	0.40
YALI0B01408g	similar to sp|P30574 Candida albicans Carboxypeptidase Y precursor (EC 3.4.16.5) (Carboxypeptidase YSCY), start by similarity	0.40
YALI0E17853g	no similarity, hypothetical start	0.40
YALI0D02167g	similar to tr|Q839Q1 Enterococcus faecalis EF0111 Oxidoreductase, zinc-binding, start by similarity	0.40
YALI0B14014g	weakly similar to tr|Q8NK56 Cryptococcus neoformans SMG1, start by similarity	0.40
YALI0B12078g	similar to sp|Q02046 Saccharomyces cerevisiae Methylenetetrahydrofolate dehydrogenase [NAD+] (EC 1.5.1. 15), start by similarity	0.40
YALI0F09581g	no similarity, hypothetical start	0.40
YALI0A06974g	similar to ca|IPF6257 Candida albicans, start by similarity	0.40
YALI0E27962g	highly similar to sp|P31539 Saccharomyces cerevisiae YLL026w HSP104 heat shock protein, start by similarity	0.40
YALI0E07315g	similar to sp|P39994 Saccharomyces cerevisiae YEL020c, hypothetical start	0.39
YALI0E27049g	weakly similar to sp|Q04781 Saccharomyces cerevisiae YMR247c similarity to hypothetical protein S. pombe, hypothetical start	0.39
YALI0E27137g	similar to tr|Q12383 Saccharomyces cerevisiae YOL125W, hypothetical start	0.39
YALI0D12386g	similar to sp|P53111 Saccharomyces cerevisiae YGL157w and DEHA0A06347g Debaryomyces hansenii, start by similarity	0.39
YALI0D19360g	no similarity, hypothetical start	0.39
YALI0E00308g	weakly similar to sp|P38680 Neurospora crassa MTR N-amino acid transport system protein (Methyltryptophan resistance protein), possible transmembrane segments, hypothetical start	0.39
YALI0F22055g	similar to sp|P40487 Saccharomyces cerevisiae YIL103w, hypothetical start	0.39
YALI0D09889g	no similarity	0.39
YALI0E24387g	no similarity, hypothetical start	0.39
YALI0A17020g	no similarity, hypothetical start	0.39

YALI0B09625g		weakly similar to sp|P09368 Saccharomyces cerevisiae YLR142w PUT1 Proline oxidase, mitochondrial precursor (EC 1.5.3.-) (Proline dehydrogenase), hypothetical start	0.38
YALI0A18568g		weakly similar to tr|Q02770 Saccharomyces cerevisiae YPL064c CWC27 putative peptidyl-prolyl cis-trans isomerase, hypothetical start	0.38
YALI0E08184g		similar to DEHA0E16819g Debaryomyces hansenii IPF 11574.1, hypothetical start	0.38
YALI0C20977g		no similarity, hypothetical start	0.38
YALI0E18348g		similar to sp|P14065 Saccharomyces cerevisiae YOR120w GCY1 galactose-induced protein of aldo/keto reductase family, hypothetical start	0.38
YALI0A02416g		sp|P41927 Yarrowia lipolytica MT1_YARLI Metallothionein-I (MT-I)	0.37
YALI0B02794g		similar to sp|P25381 Saccharomyces cerevisiae Related to subtilase-type proteinase, hypothetical start	0.37
YALI0D04290g		weakly similar to sp|P40990 Saccharomyces cerevisiae YDL107w MSS2 ser/thr protein kinase, start by similarity	0.37
YALI0B02112g		no similarity, hypothetical start	0.37
YALI0D15114g		similar to tr|Q96UC2 Neurospora crassa Conserved hypothetical protein, start by similarity	0.37
YALI0E33429g		weakly similar to YG59_METJA sp|Q59053 Methanococcus jannaschii Hypothetical ATP-binding protein MJ1659, hypothetical start	0.37
YALI0F29909g		weakly similar to sp|Q03104 Saccharomyces cerevisiae YML128c GIN3 unknown function, start by similarity	0.37
YALI0E03278g		similar to sp|Q03254 Saccharomyces cerevisiae YMR277w FCP1 TFIIF interacting component of CTD phosphatase and DEHA0F03498g Debaryomyces hansenii, possible transmembrane segment, hypothetical start	0.36
YALI0D10285g		weakly similar to sp|P53971 Saccharomyces cerevisiae YNL023c FAP1 Shuttle craft like transcriptional regulator with 8 zf-NF-X1 zinc fingers and R3H domain, start by similarity	0.36
YALI0C21956g		similar to sp|P53145 Saccharomyces cerevisiae YGL099w, start by similarity	0.36
YALI0D21010g		similar to KLLA0B05709g Kluyveromyces lactis IPF 7503.1, hypothetical start	0.36
YALI0E34265g		similar to sp|P06115 Saccharomyces cerevisiae YGR088w CTT1 catalase T, cytosolic P2.391.f2.1, hypothetical start	0.36
YALI0B22660g	YlPEX19	tr|Q96W74 Yarrowia lipolytica Peroxin required for biogenesis of peroxisomes, identified start	0.36
YALI0E05137g		highly similar to sp|O13302 Ajellomyces capsulata IDH1 Isocitrate dehydrogenase [NAD] subunit 1, mitochondrial precursor, hypothetical start	0.36
YALI0C12364g		some similarities with sp|Q03125 Saccharomyces cerevisiae YDR043c NRG1 transcriptional repressor for glucose repression of STA1 gene expression, hypothetical start	0.36
YALI0E24189g		no similarity, hypothetical start	0.36
YALI0B14487g		similar to tr|Q871M5 Neurospora crassa B20D17.110 Probable nucleosome assembly protein I, start by similarity	0.36

YALI0C20060g		similar to sp|P41928 Yarrowia lipolytica MTP2 Metallothionein-II, start by similarity	0.36
YALI0C01738g		similar to sp|P35184 Saccharomyces cerevisiae Ribosome assembly protein SQT1, start by similarity	0.36
YALI0B13156g	YlCNX1	tr|Q9HFC6 Yarrowia lipolytica Calnexin precursor, identified start	0.35
YALI0E14190g		similar to sp|P17505 Saccharomyces cerevisiae YKL085w MDH1 malate dehydrogenase precursor, mitochondrial and tr|O94137 Piromyces sp. Malate dehydrogenase (EC 1.1.1. 37), no start	0.35
YALI0D21648g		some similarities with DEHA0G17006g Debaryomyces hansenii IPF 273.1, hypothetical start	0.35
YALI0C20669g		similar to tr|Q9C2D7 Neurospora crassa Related to lincomycin-condensing protein lmbA, start by similarity	0.35
YALI0D16753g		highly similar to tr|Q8TG27 Talaromyces emersonii Malate dehydrogenase, start by similarity	0.35
YALI0D19162g	YlRIM8	tr|Q9UVF5 Yarrowia lipolytica Pal3 protein, involved in ambient pH sensing, mating and meiosis, identified start	0.35
YALI0F18854g		similar to sp|Q03829 Saccharomyces cerevisiae Putative mitochondrial carrier YMR166C, start by similarity	0.35
YALI0C21021g		similar to tr|Q8NJZ8 Emericella nidulans Theta class glutathione S-transferase (EC 2.5.1.18), start by similarity	0.35
YALI0C00649g		weakly similar to DEHA0D03388g Debaryomyces hansenii or DEHA0D03410g Debaryomyces hansenii, start by similarity	0.35
YALI0F12133g		no similarity	0.35
YALI0A16291g		weakly similar to tr|Q06681 Saccharomyces cerevisiae YDR326c, hypothetical start	0.35
YALI0D22957g		weakly similar to sp|Q12303 Saccharomyces cerevisiae YLR121c YPS3 GPI-anchored aspartyl protease 3 (yapsin 3), hypothetical start	0.34
YALI0F18282g		similar to tr|Q8TFK5 Yarrowia lipolytica Cell wall protein, start by similarity	0.34
YALI0B22176g		no similarity, hypothetical start	0.34
YALI0D19272g		weakly similar to sp|Q09710 Schizosaccharomyces pombe Hypothetical protein C18B11.03c in chromosome I, hypothetical start	0.34
YALI0F23793g		similar to sp|Q04458 Saccharomyces cerevisiae YMR110c, start by similarity	0.34
YALI0A02673g		similar to CA1925|IPF18587 Candida albicans putative methyltransferase, start by similarity	0.34
YALI0E27715g		similar to sp|P24783 Saccharomyces cerevisiae YNL112w DBP2 ATP-dependent RNA helicase of DEAD box family, hypothetical start	0.34
YALI0B05522g		similar to tr|Q96TK5 Coccidioides immitis Aspartyl aminopeptidase, start by similarity	0.33
YALI0B07535g		weakly similar to sp|P19158 Saccharomyces cerevisiae YOL081w IRA2 GTPase-activating protein for RAS proteins, hypothetical start	0.33
YALI0C04477g		similar to tr|Q8X1Y6 Debaryomyces occidentalis SCR1 protein, start by similarity	0.33

YALI0A00847g	similar to tr|O59826 Schizosaccharomyces pombe Putative potassium channel subunit, start by similarity	0.32
YALI0C05687g	no similarity, hypothetical start	0.32
YALI0A15147g	similar to tr|Q9UW06 Yarrowia lipolytica Alcohol dehydrogenase 3, hypothetical start	0.32
YALI0B16192g	similar to sp|P87219 Candida albicans Sorbitol utilization protein SOU1 or sp|P87218 Candida albicans Sorbitol utilization protein SOU2, hypothetical start	0.32
YALI0F04620g	no similarity, hypothetical start	0.32
YALI0C22088g	similar to sp|P38716 Saccharomyces cerevisiae YHR112c similarity to cystathionine gamma-synthases P5.26. f3.1, start by similarity	0.32
YALI0D19426g	weakly similar to sp|P53332 Saccharomyces cerevisiae Hypothetical 34.3 kDa protein in TAF145-YOR1 intergenic region, hypothetical start	0.32
YALI0C05797g	no similarity, hypothetical start	0.32
YALI0E26873g	weakly similar to DEHA0E18865g Debaryomyces hansenii, hypothetical start	0.32
YALI0C16995g	highly similar to sp|P10963 Saccharomyces cerevisiae YKR097w PCK1 phosphoenolpyruvate carboxykinase, hypothetical start	0.31
YALI0B13354g	weakly similar to sp|P17106 Saccharomyces cerevisiae YJR060w CBF1 kinetochore protein, hypothetical start	0.31
YALI0D13662g	no similarity	0.31
YALI0E19745g	no similarity	0.31
YALI0F09119g	some similarities with DEHA0C11132g Debaryomyces hansenii, hypothetical start	0.31
YALI0F10175g	no similarity, hypothetical start	0.30
YALI0B21318g	no similarity, hypothetical start	0.30
YALI0F00682g	similar to tr|Q04432 Saccharomyces cerevisiae YDR533c hypothetical protein and CAGL0C00275g Candida glabrata and KLLA0D00704g Kluyveromyces lactis or KLLA0D00682g Kluyveromyces lactis, start by similarity	0.30
YALI0F08415g	similar to wi|NCU00678.1 Neurospora crassa and sp|Q04792 Saccharomyces cerevisiae YMR250w, start by similarity	0.30
YALI0C01221g	similar to tr|Q9P3P6 Neurospora crassa Related to 26s proteasome subunit p28, start by similarity	0.30
YALI0C02343g	similar to DEHA0B14806g Debaryomyces hansenii IPF 512.1, start by similarity	0.30
YALI0E34441g	no similarity, hypothetical start	0.30
YALI0B22308g	highly similar to tr|O74996 Yarrowia lipolytica Hexokinase, hypothetical start	0.30
YALI0E29381g	no similarity, hypothetical start	0.30
YALI0E07535g	similar to KLLA0F00704g Kluyveromyces lactis, hypothetical start	0.29
YALI0B15334g	weakly similar to tr|Q12483 Saccharomyces cerevisiae YPL002c SNF8 involved in glucose derepression singleton, start by similarity	0.29
YALI0D04510g	no similarity, hypothetical start	0.29
YALI0E05819g	weakly similar to sp|P08640 Saccharomyces cerevisiae YIR019c STA1 extracellular	0.29

	alpha-1, 4-glucan glucosidase, hypothetical start	
YALI0E15378g	tr|Q9P4D9 Yarrowia lipolytica Multifunctional beta-oxidation enzyme, identified start	0.29
YALI0A07733g	similar to tr|Q8WZH4 Neurospora crassa Probable DELTA3, 5-DELTA2, 4-dienoyl-CoA isomerase precursor (ECH1), start by similarity	0.29
YALI0F22561g	no similarity, hypothetical start	0.29
YALI0E34749g	similar to DEHA0B16379g Debaryomyces hansenii IPF 367.1, start by similarity	0.28
YALI0F26059g	weakly similar to sp|Q12524 Saccharomyces cerevisiae YLR151c PCD1 peroxisomal nudix hydrolase, hypothetical start	0.28
YALI0B20636g	weakly similar to tr|Q89QU1 Bradyrhizobium japonicum Bll3033 protein, hypothetical start	0.28
YALI0D11286g	no similarity, hypothetical start	0.28
YALI0E15400g	similar to sp|Q04458 Saccharomyces cerevisiae YMR110c similarity to aldehyde dehydrogenase, hypothetical start	0.27
YALI0D03245g	no similarity, hypothetical start	0.27
YALI0D04268g	similar to sp|P00431 Saccharomyces cerevisiae YKR066c CCP1 cytochrome-c peroxidase precursor, hypothetical start	0.27
YALI0F10219g	weakly similar to tr|Q9YBD7 Aeropyrum pernix Hypothetical protein APE1660 and DEHA0B08569g Debaryomyces hansenii, hypothetical start	0.27
YALI0B13904g	similar to sp|P53327 Saccharomyces cerevisiae YGR271w strong similarity to S.pombe RNA helicase or sp|P32639 Saccharomyces cerevisiae YER172c BRR2 RNA helicase-related protein, hypothetical start	0.27
YALI0E20427g	similar to wi|NCU05585.1 Neurospora crassa NCU05585. 1, hypothetical start	0.27
YALI0F00990g	no similarity, hypothetical start	0.27
YALI0D19338g	no similarity	0.26
YALI0C06259g	no similarity	0.26
YALI0C09658g	no similarity, hypothetical start	0.26
YALI0D12661g	similar to sp|Q9WU19 Mus musculus Hydroxyacid oxidase 1 (EC 1.1.3.15) (HAOX1), start by similarity	0.26
YALI0E19657g	weakly similar to sp|P53839 Saccharomyces cerevisiae YNL274c similarity to glycerate- and formate-dehydrogenases, start by similarity	0.25
YALI0C01287g	similar to tr|Q86TN4 Homo sapiens tRNA splicing 2 phosphotransferase 1, hypothetical start	0.25
YALI0E02310g	highly similar to sp|P40581 Saccharomyces cerevisiae YIR037w HYR1 glutathione peroxidase, hypothetical start	0.25
YALI0F08129g	weakly similar to tr|AAO79986 Enterococcus faecalis EF0111 gene Oxidoreductase, zinc-binding and sp|P28625 Saccharomyces cerevisiae YMR152w YIM1 mitochondrial inner membrane protease, start by similarity	0.25
YALI0E18326g	similar to sp|P36119 Saccharomyces cerevisiae YKR023w Hypothetical 60.8 kDa protein, hypothetical start	0.25

YALI0C15004g		no similarity, hypothetical start	0.24
YALI0D16291g		similar to sp|P53893 Saccharomyces cerevisiae YNL163c translation elongation factor eEF4, start by similarity	0.24
YALI0A17061g		similar to DEHA0C06897g Debaryomyces hansenii, hypothetical start	0.24
YALI0B18524g		some similarities with sp|P53137 Saccharomyces cerevisiae Hypothetical 72.0 kDa protein in TAF60-MLC1 intergenic region, hypothetical start	0.24
YALI0A00682g		no similarity, hypothetical start	0.23
YALI0A00176g		weakly similar to sp|P08640 Saccharomyces cerevisiae YIR019c STA1 extracellular alpha-1, 4-glucan glucosidase, start by similarity	0.23
YALI0E18568g	YlPOT1	sp|Q05493 Yarrowia lipolytica 3-ketoacyl-CoA thiolase, peroxisomal precursor, identified start	0.23
YALI0C04543g		weakly similar to tr|Q05468 Saccharomyces cerevisiae Chromosome IV COSMID 9651, hypothetical start	0.23
YALI0F09273g		similar to sp|P43550 Saccharomyces cerevisiae YFL053w DAK2 Dihydroxyacetone kinase 2 or sp|P54838 Saccharomyces cerevisiae YML070w DAK1 dihydroxyacetone kinase and DEHA0A03971g Debaryomyces hansenii, start by similarity	0.23
YALI0D20526g		weakly similar to sp|P22943 Saccharomyces cerevisiae 12 kDa heat shock protein (Glucose and lipid-regulated protein), hypothetical start	0.23
YALI0D13104g		similar to sp|P41805 Saccharomyces cerevisiae YLR075w GRC5 60S large subunit ribosomal protein, start by similarity	0.22
YALI0A21373g		no similarity, hypothetical start	0.22
YALI0F26917g		similar to DEHA0F11385g Debaryomyces hansenii, start by similarity	0.22
YALI0E31493g		weakly similar to tr|Q9C280 Neurospora crassa Conserved hypothetical protein, hypothetical start	0.22
YALI0E27940g		highly similar to sp|Q12522 Saccharomyces cerevisiae YPR016c TIF6 translation initiation factor 6 (eIF6), start by similarity	0.22
YALI0D27236g		weakly similar to tr|Q9XTH4 Caenorhabditis elegans Hypothetical protein K08H10.2a, hypothetical start	0.22
YALI0E11495g		similar to sp|P15274 Saccharomyces cerevisiae YML035c AMD1 AMP deaminase, start by similarity	0.22
YALI0A09383g		weakly similar to tr|Q9P6J0 Saccharomyces cerevisiae YGR260w TNA1 related to allantoate transport protein, start by similarity	0.21
YALI0E21307g		similar to sp|P00175 Saccharomyces cerevisiae YML054c CYB2 lactate dehydrogenase cytochrome b2, hypothetical start	0.21
YALI0F02497g		similar to sp|Q12428 Saccharomyces cerevisiae YPR002W Hypothetical 57.7 kDa protein in CIT3-HAL1 intergenic region, start by similarity	0.21
YALI0F12089g		similar to sp|P39109 Saccharomyces cerevisiae YDR135c YCF1 glutathione S-conjugate transporter vacuolar, hypothetical start	0.21

YALI0E13860g		weakly similar to sp|Q12303 Saccharomyces cerevisiae YLR121c YPS3 GPI-anchored aspartyl protease 3 (yapsin 3), hypothetical start	0.21
YALI0E09064g		similar to DEHA0F07326g Debaryomyces hansenii IPF 8538.1, hypothetical start	0.21
YALI0F08283g		weakly similar to sp|P53164 Saccharomyces cerevisiae YGL067w NPY1 NADH pyrophosphatase I, start by similarity	0.21
YALI0E14817g		similar to sp|P34220 Saccharomyces cerevisiae YBL055c, hypothetical start	0.21
YALI0D18678g		weakly similar to sp|P46974 Saccharomyces cerevisiae YJR127c ZMS1 transcription factor with similarity to regulatory protein ARD1P P3.74.f3.1, hypothetical start	0.21
YALI0B10087g		some similarities with KLLA0B09350g Kluyveromyces lactis IPF 7260.1, hypothetical start	0.20
YALI0E24057g		similar to sp|P50107 Saccharomyces cerevisiae YML004c GLO1 glyoxalase I, hypothetical start	0.20
YALI0F14817g		weakly similar to tr|O13652 Schizosaccharomyces pombe Hypothetical 102.0 kDa protein (PI059 protein), hypothetical start	0.20
YALI0A05885g		no similarity, hypothetical start	0.20
YALI0E27808g		similar to sp|P38911 Saccharomyces cerevisiae YML074c NPI46 proline cis-trans isomerase, hypothetical start	0.20
YALI0B10406g		similar to tr|Q9V6U5 Drosophila melanogaster LD24265p, hypothetical start	0.19
YALI0C06424g		similar to sp|P10870 Saccharomyces cerevisiae YDL194w SNF3 high-affinity glucose transporter/regulatory protein, start by similarity	0.19
YALI0E20405g		weakly similar to tr|O48868 Populus balsamifera 4-coumarate:CoA ligase 2, hypothetical start	0.18
YALI0E34419g		no similarity, hypothetical start	0.18
YALI0E00638g		similar to tr|Q9TEM3 Emericella nidulans MCSA Methylcitrate synthase precursor, hypothetical start	0.17
YALI0D18964g		similar to sp|P87218 Candida albicans Sorbitol utilization protein SOU2, hypothetical start	0.16
YALI0A08998g		similar to tr|Q9P702 Neurospora crassa Probable sugar transporter, hypothetical start	0.16
YALI0D24475g		no similarity, possibly noncoding, hypothetical start	0.15
YALI0A21285g		no similarity, hypothetical start	0.15
YALI0C06534g		no similarity	0.15
YALI0B12980g		similar to tr|Q96VK4 Emericella nidulans ABC transporter protein, start by similarity	0.12
YALI0B15312g		similar to sp|P53863 Saccharomyces cerevisiae YNL227c similarity to dnaJ-like proteins P10.5.f2.1, start by similarity	0.12
YALI0B10021g		similar to sp|P38861 Saccharomyces cerevisiae YHR170w NMD3 nonsense-mediated mRNA decay protein singleton, hypothetical start	0.12
YALI0A20350g	YlLIP2	tr|Q9P8F7 Yarrowia lipolytica Triacylglycerol lipase precursor, identified start	0.10
YALI0B08734g		similar to CAGL0I09350g Candida glabrata and sp|P38344 Saccharomyces cerevisiae 45.8 kDa protein in SHM1-MRPL37 intergenic region, hypothetical start	0.09
YALI0A21417g		similar to sp|O74267 Ashbya gossypii Threonine aldolase, hypothetical start	0.07

YALI0E02134g	similar to sp|P48510 Saccharomyces cerevisiae YMR276w DSK2 ubiquitin-like protein, possible transmembrane segment, hypothetical start	0.04
YALI0B04906g	similar to tr|Q96UC6 Neurospora crassa Conserved hypothetical protein, start by similarity	0.04
YALI0F25597g	similar to tr|Q8X1Y6 Debaryomyces occidentalis SCR1 protein and tr|Q9URI1 Candida albicans Multidrug resistance protein, hypothetical start	0.04
YALI0A21439g	no similarity	0.03
YALI0A21307g	similar to sp|P25297 Saccharomyces cerevisiae YML123c PHO84 high-affinity inorganic phosphate/H+ symporter, hypothetical start	0.02
YALI0A21461g	similar to sp|P36114 Saccharomyces cerevisiae YKR018c, hypothetical start	0.02
YALI0A21329g	weakly similar to CAGL0L02475g Candida glabrata, no start	0.01

Table S3. Genes up-regulated (112) during the yeast to hyphae transition at 15 minues induced by GlNAc mediated by ZNC1.

Locus tag	Gene	Description	Fold change
YALI0B07051g		no similarity, hypothetical start	20.34
YALI0A20438g		similar to sp|P36091 Saccharomyces cerevisiae Hypothetical 49.6 kDa protein in ELM1-PRI2 intergenic region, hypothetical start	12.08
YALI0F13937g		highly similar to tr|O93968 Candida boidinii Formate dehydrogenase or tr|O13437 Candida boidinii NAD-dependent formate dehydrogenase, start by similarity	10.60
YALI0E01100g		similar to sp|P21304 Saccharomyces cerevisiae YLR196w PWP1, start by similarity	9.86
YALI0D04048g		similar to sp|P40073 Saccharomyces cerevisiae YER118c SSU81 involved in the HOG1 high-osmolarity signal transduction pathway, hypothetical start	9.44
YALI0E23947g		similar to sp|P07703 Saccharomyces cerevisiae YPR110c RPC40 DNA-directed RNA polymerase I and III 40 kDa polypeptide, hypothetical start	9.00
YALI0C11165g		no similarity, hypothetical start	8.97
YALI0D14410g		weakly similar to DEHA0C15796g Debaryomyces hansenii IPF 3508.1, start by similarity	7.43
YALI0B04092g		similar to sp|Q03219 Saccharomyces cerevisiae Hypothetical 31.1 kDa protein in SIP18-SPT21 intergenic region, hypothetical start	6.99
YALI0B08052g		similar to DEHA0A06347g Debaryomyces hansenii, start by similarity	6.11
YALI0E26587g		similar to sp|P38786 Saccharomyces cerevisiae YHR062c RPP1 required for processing of tRNA and 35S rRNA, hypothetical start	6.05
YALI0E27390g		no similarity, possibly noncoding, hypothetical start	5.95
YALI0E31383g		weakly similar to KLLA0A04169g Kluyveromyces lactis IPF 8725.1, hypothetical start	5.81
YALI0F26367g		similar to sp|P36100 Saccharomyces cerevisiae YKL028w TFA1 large subunit of transcription factor TFIIE, hypothetical start	5.60
YALI0B10758g		highly similar to sp|P43063 Candida albicans cell division control protein 28, hypothetical start	5.56
YALI0E01210g		no similarity, hypothetical start	5.41
YALI0D25476g		similar to tr|O93921 Emericella nidulans Pantothenate kinase (EC 2.7.1.33), hypothetical start	5.32
YALI0F24717g		no similarity, hypothetical start	5.10
YALI0E23859g		similar to sp|P38361 Saccharomyces cerevisiae YBR296c PHO89 Na+-coupled phosphate transport protein, high affinity, hypothetical start	5.09
YALI0C09232g		some similarities with tr|Q12427 Saccharomyces cerevisiae YDR169c STB3 SIN3 protein-binding protein, hypothetical start	5.03
YALI0F03410g		no similarity, hypothetical start	4.99
YALI0F23485g		some similarities with wi|NCU02191.1 Neurospora crassa NCU02191.1 hypothetical protein, hypothetical start	4.93
YALI0F30393g		similar to sp|P25586 Saccharomyces cerevisiae YCL059c KRR1 unknown function, start	4.93

		by similarity	
YALI0D02189g		weakly similar to tr|Q05518 Saccharomyces cerevisiae YDR348c, hypothetical start	4.11
YALI0A15488g	YlALK7	tr|O74133 Yarrowia lipolytica ALK7 cytochrome P450, identified start	4.02
YALI0B10560g		similar to sp|P40991 Saccharomyces cerevisiae YNL061w NOP2 nucleolar protein, start by similarity	3.99
YALI0A18524g		no similarity, hypothetical start, frameshift	3.93
YALI0E14971g		no similarity, hypothetical start	3.74
YALI0E31779g		similar to CA0241|IPF15630 Candida albicans IPF15630, hypothetical start	3.73
YALI0A12925g		weakly similar to sp|P32338 Saccharomyces cerevisiae Zinc finger protein RME1, hypothetical start	3.71
YALI0E12683g		highly similar to sp|P05694 Saccharomyces cerevisiae YER091c MET6 5-methyltetrahydropteroyltriglutamate--homocysteine methyltransferase, start by similarity	3.60
YALI0F18260g		some similarities with tr|Q03973 Saccharomyces cerevisiae YDR174w HMO1 Non-histone protein, hypothetical start	3.55
YALI0F28193g		similar to sp|P53322 Saccharomyces cerevisiae YGR260w TNA1 similarity to allantoate transport protein, hypothetical start	3.41
YALI0E02904g		similar to sp|Q01159 Saccharomyces cerevisiae YGL130w CEG1 mRNA guanylyltransferase (mRNA capping enzyme, alpha subunit), hypothetical start	3.33
YALI0F00462g		similar to sp|O43315 Homo sapiens Aquaporin 9 (Small solute channel 1), hypothetical start	3.29
YALI0B19492g		similar to tr|Q8TGV8 Candida albicans Gap1 protein, start by similarity	3.28
YALI0A02981g		weakly similar to tr|Q9C232 Neurospora crassa Conserved hypothetical protein, hypothetical start	3.24
YALI0B09779g		highly similar to DEHA0G23012g Debaryomyces hansenii IPF 4913.1, start by similarity	3.20
YALI0D11792g		similar to CA0750|IPF6284 Candida albicans IPF6284 unknown function, hypothetical start	3.19
YALI0D05159g		similar to sp|P38174 Saccharomyces cerevisiae YBL091c MAP2 methionine aminopeptidase, isoform 2, start by similarity	3.19
YALI0D11396g		similar to sp|P35718 Saccharomyces cerevisiae YKL144c RPC25 DNA-direcred RNA polymerase III, 25 KD subunit singleton, start by similarity	3.16
YALI0A03861g		weakly similar to sp|P34218 Saccharomyces cerevisiae YBL052c SAS3 silencing protein, start by similarity	3.08
YALI0E04763g		similar to sp|Q12074 Saccharomyces cerevisiae YPR069c SPE3 putrescine aminopropyltransferase (spermidine synthase) or sp|Q12455 Saccharomyces cerevisiae YLR146c SPE4 spermine synthase, start by similarity	3.03
YALI0E27715g		similar to sp|P24783 Saccharomyces cerevisiae YNL112w DBP2 ATP-dependent RNA helicase of DEAD box family, hypothetical start	3.01
YALI0E03366g		similar to sp|P38332 Saccharomyces cerevisiae YBR246w, start by similarity	3.01

YALI0A18183g		similar to sp|P19807 Saccharomyces cerevisiae YGL077c HNM1 choline permease, hypothetical start	2.98
YALI0B18414g		weakly similar to tr|Q8BH76 Mus musculus DNA polymerase delta subunit 3, start by similarity	2.98
YALI0C00473g		weakly similar to DEHA0F03234g Debaryomyces hansenii IPF 8870.1, hypothetical start	2.96
YALI0F15785g		similar to sp|P41896 Saccharomyces cerevisiae YGR005c TFG2 TFIIF subunit (transcription initiation factor), 54 kD, hypothetical start	2.95
YALI0E20515g		weakly similar to DEHA0G10219g Debaryomyces hansenii, hypothetical start	2.92
YALI0C19822g		no similarity	2.91
YALI0F24145g		weakly similar to wi|NCU01815.1 Neurospora crassa NCU01815.1 predicted protein, hypothetical start	2.91
YALI0F28171g		no similarity, possibly noncoding, hypothetical start	2.89
YALI0B17732g		weakly similar to tr|O94345 Schizosaccharomyces pombe Putative mitochondrial ribosomal protein and sp|P22353 Saccharomyces cerevisiae YJL063c MRPL8 ribosomal protein L17, mitochondrial, hypothetical start	2.88
YALI0A20614g		similar to tr|Q06506 Saccharomyces cerevisiae of the beta-transducin family of guanine nucleotide-binding, hypothetical start	2.86
YALI0C02233g		no similarity, hypothetical start	2.82
YALI0D24343g		weakly similar to sp|P47045 Saccharomyces cerevisiae Hypothetical 54.2 kDa protein in BTN1-PEP8 intergenic region, hypothetical start	2.82
YALI0E31647g		similar to CA4625|IPF5742 Candida albicans IPF5742 thioredoxin-like protein (by homology), hypothetical start	2.80
YALI0F21483g		highly similar to tr|Q9UVE8 Yarrowia lipolytica Hypothetical 39.4 kDa protein, hypothetical start	2.73
YALI0D08140g		similar to sp|P36110 Saccharomyces cerevisiae YKR013w PRY2 protein precursor (Pathogen related in Sc 2), start by similarity	2.69
YALI0E12353g		weakly similar to sp|P32387 Saccharomyces cerevisiae YDR405w MRP20 60S ribosomal protein L41, mitochondrial [Precursor], hypothetical start	2.67
YALI0A05379g		similar to ca|CA0220|CaABZ1 Candida albicans para-aminobenzoate synthase, start by similarity	2.67
YALI0E05533g	YlLYC1	sp|P41929 Lysine acetyltransferase, identified start	2.65
YALI0C20251g		similar to sp|Q12068 Saccharomyces cerevisiae YOL151w GRE2, start by similarity	2.64
YALI0A09449g		weakly similar to sp|P41734 Saccharomyces cerevisiae YOR126c EST2 isoamyl acetate hydrolytic enzyme, start by similarity	2.62
YALI0A16720g		no similarity, hypothetical start	2.61
YALI0C10802g		some similarities with sp|P36521 Saccharomyces cerevisiae 60S ribosomal protein L11 mitochondrial precursor (YmL11), hypothetical start	2.57
YALI0E10483g		similar to DEHA0A10670g Debaryomyces hansenii, hypothetical start	2.56

YALI0D09163g	some similarities with tr|Q9FIE4 Arabidopsis thaliana Hypothetical protein, start by similarity	2.55
YALI0D13860g	similar to tr|Q06504 Saccharomyces cerevisiae YPR131c NAT3 N-acetyltransferase complex subunit ARD1P, start by similarity	2.53
YALI0C11341g	highly similar to tr|Q12230 Saccharomyces cerevisiae Hypothetical 38.1 kDa protein YPL004C, hypothetical start	2.50
YALI0B16214g	similar to sp|P25379 Saccharomyces cerevisiae YCL064c CHA1 L-serine/L-threonine deaminase P3.118.f2.1, hypothetical start	2.50
YALI0C19404g	some similarities with tr|Q06525| Saccharomyces cerevisiae YPR152c, start by similarity	2.49
YALI0D19140g	similar to sp|P30952 Saccharomyces cerevisiae YNL117w Malate synthase 1, glyoxysomal (EC 4.1.3.2), hypothetical start	2.48
YALI0B02222g	similar to sp|Q12184 Saccharomyces cerevisiae YPL252c Adrenodoxin homolog, mitochondrial precursor (Mitochondrial ferredoxin), hypothetical start	2.46
YALI0F26565g	some similarities with DEHA-IPF6705 Debaryomyces hansenii, hypothetical start	2.44
YALI0B08426g	no similarity, hypothetical start	2.43
YALI0F06116g	similar to sp|P32864 Saccharomyces cerevisiae YOR370c, start by similarity	2.41
YALI0C02717g	similar to KLLA0E08767g Kluyveromyces lactis, start by similarity	2.41
YALI0E26059g	weakly similar to CA5416|IPF1576 Candida albicans, hypothetical start	2.40
YALI0D03641g	no similarity, hypothetical start	2.37
YALI0B04488g	similar to sp|O14188 Schizosaccharomyces pombe Ras GTPase-activating-like protein rng2 (Ring assembly protein 2), hypothetical start	2.36
YALI0D17116g	highly similar to sp|Q12460 Saccharomyces cerevisiae YLR197w SIK1 involved in pre-rRNA processing, start by similarity	2.36
YALI0B10153g	similar to tr|Q9HF05 Emericella nidulans Oleate delta-12 desaturase, hypothetical start	2.35
YALI0C10450g	weakly similar to sp|Q04018 Saccharomyces cerevisiae YMR244w Hypothetical 37.4 kDa protein, hypothetical start	2.31
YALI0E08756g	similar to DEHA0F10351g Debaryomyces hansenii, no start	2.31
YALI0F23089g	similar to sp|P87041 Schizosaccharomyces pombe UDP-galactose transporter (Golgi UDP-Gal transporter), start by similarity	2.29
YALI0A13585g	weakly similar to sp|P38069 Saccharomyces cerevisiae YBR015c TTP1(MNN2), hypothetical start	2.29
YALI0A03905g	highly similar to sp|P36160 Saccharomyces cerevisiae YKR081c, start by similarity	2.28
YALI0F08833g	weakly similar to sp|P15424 Saccharomyces cerevisiae YDR194c MSS116 RNA helicase of the DEAD box family, hypothetical start	2.28
YALI0F22759g	weakly similar to ca|CA5787|IPF1136 Candida albicans unknown function, hypothetical start	2.27
YALI0E00550g	similar to sp|P36105 Saccharomyces cerevisiae YKL006w RPL14A ribosomal protein or sp|P38754 Saccharomyces cerevisiae YHL001w RPL14B ribosomal protein, hypothetical	2.26

		start	
YALI0B03784g		similar to tr|Q9HEC4 Neurospora crassa Conserved hypothetical protein, hypothetical start	2.25
YALI0C19624g		similar to sp|P38071 Saccharomyces cerevisiae YBR026c (MRF1) mitochondrial respiratory function protein, hypothetical start	2.24
YALI0B20328g		similar to sp|P39979 Saccharomyces cerevisiae YEL066w HPA3 histone and other protein acetyltransferase, hypothetical start	2.23
YALI0E13486g		highly similar to sp|O13473 Kluyveromyces lactis Centromere/microtubule binding protein CBF5 and KLLA0D04796g Kluyveromyces lactis IPF 5085.1, hypothetical start	2.22
YALI0F05258g		similar to sp|P40481 Saccharomyces cerevisiae YIL110w, start by similarity	2.21
YALI0F24959g		similar to sp|P05754 Saccharomyces cerevisiae YER102w RPS8B ribosomal protein S8B or sp|P05754 Saccharomyces cerevisiae YBL072c RPS8A ribosomal protein S8A, start by similarity	2.21
YALI0D03069g		similar to sp|P04161 Saccharomyces cerevisiae YDR408c ADE8 phosphoribosylglycinamide formyltransferase (GART), start by similarity	2.19
YALI0B15304g		similar to sp|Q12698 Saccharomyces kluyveri Amidophosphoribosyltransferase (EC 2.4.2.14)	2.18
YALI0D25828g		similar to sp|Q08234 Saccharomyces cerevisiae Probable ATP-dependent transporter YOL074C/YOL075C, hypothetical start	2.18
YALI0D16687g		weakly similar to tr|Q9FY91 Arabidopsis thaliana SIR2-family protein, hypothetical start	2.17
YALI0F24167g		no similarity, hypothetical start	2.17
YALI0E25047g		no similarity, hypothetical start	2.16
YALI0B21824g		weakly similar to tr|Q9UW95 Aspergillus parasiticus Cytochrome P450 monooxygenase, start by similarity	2.16
YALI0F00704g		no similarity, hypothetical start	2.12
YALI0E14707g		no similarity, hypothetical start	2.11
YALI0D11462g		similar to tr|Q06631 Saccharomyces cerevisiae YDR299w BFR2 involved in protein transport steps at the Brefeldin A blocks singleton, start by similarity	2.07
YALI0C21362g		similar to sp|P39676 Saccharomyces cerevisiae YGR234w YHB1 flavohemoglobin, start by similarity	2.07
YALI0E01584g		similar to DEHA0E11000g Debaryomyces hansenii, hypothetical start	2.06
YALI0E13277g	YlEF-3	tr|O93814 Elongation factor 3 (EF-3), hypothetical start	2.05
YALI0D00473g		no similarity, hypothetical start	2.02

Table S4. Genes down-regulated (59) during the yeast to hyphae transition at 15 minues induced by GlNAc mediated by ZNC1.

Locus tag	Gene	Description	Fold change
YALI0C20405g		similar to sp|P10768 Homo sapiens Esterase D (EC 3. 1.1.1), hypothetical start	0.49
YALI0F25289g		highly similar to sp|P22202 Saccharomyces cerevisiae YER103w SSA4 heat shock protein of HSP70 family, start by similarity	0.49
YALI0E19745g		no similarity	0.48
YALI0E11517g		weakly similar to tr|Q8TFK5 Yarrowia lipolytica CWP1 Cell wall protein, start by similarity	0.48
YALI0C00649g		weakly similar to DEHA0D03388g Debaryomyces hansenii or DEHA0D03410g Debaryomyces hansenii, start by similarity	0.47
YALI0F25487g		weakly similar to tr|O07408 Mycobacterium tuberculosis hypothetical protein and tr|Q9HU04 Pseudomonas aeruginosa hypothetical protein and tr|Q9DCP4 Mus musculus hypothetical protein, hypothetical start	0.47
YALI0E30085g		weakly similar to CA5663|IPF1251 Candida albicans IPF1251, hypothetical start	0.46
YALI0E04620g		some similarities with DEHA0D15444g Debaryomyces hansenii, hypothetical start	0.46
YALI0E15664g		weakly similar to DEHA0D10483g Debaryomyces hansenii, hypothetical start	0.46
YALI0B21318g		no similarity, hypothetical start	0.45
YALI0C12188g		no similarity, hypothetical start	0.45
YALI0A19910g		similar to sp|P47137 Saccharomyces cerevisiae Probable oxidoreductase YJR096W, hypothetical start	0.45
YALI0B12760g		no similarity, hypothetical start	0.44
YALI0D04004g		similar to sp|P47821 Saccharomyces cerevisiae RNA polymerase II holoenzyme cyclin-like subunit SSN8, hypothetical start	0.44
YALI0D12705g		similar to DEHA0B08327g Debaryomyces hansenii, hypothetical start	0.43
YALI0F29887g		no similarity, hypothetical start	0.43
YALI0C12859g		similar to sp|P17119 Saccharomyces cerevisiae YPR141c KAR3 kinesin-related protein, hypothetical start	0.42
YALI0F27005g		similar to CA0775|CaIFK2 Candida albicans CaIFK2 probable monoxygenase, hypothetical start	0.42
YALI0E29579g		similar to sp|P38628 Saccharomyces cerevisiae YEL058w PCM1 phosphoacetylglucosamine mutase singleton, hypothetical start	0.41
YALI0B20768g		similar to DEHA0A00979g Debaryomyces hansenii IPF 65.1, hypothetical start	0.41
YALI0E29403g		similar to sp|P53010 Saccharomyces cerevisiae YGL094c PAN2 component of PAB1P-stimulated poly(A) ribonuclease singleton, hypothetical start	0.40
YALI0A14322g		no similarity, hypothetical start	0.40
YALI0B20636g		weakly similar to tr|Q89QU1 Bradyrhizobium japonicum Bll3033 protein, hypothetical start	0.39
YALI0E14146g		some similarities with tr|Q96WV4 Schizosaccharomyces pombe SPBPJ4664.05 Unspecified membrane protein, hypothetical start	0.39

YALI0E34265g		similar to sp|P06115 Saccharomyces cerevisiae YGR088w CTT1 catalase T, cytosolic P2.391.f2.1, hypothetical start	0.38
YALI0F22605g		similar to CA1311|IPF14233 Candida albicans IPF14233 and DEHA-IPF10476.1 Debaryomyces hansenii, start by similarity	0.38
YALI0A09812g		similar to sp|Q04371 Saccharomyces cerevisiae YMR027w HRT2 high level expression reduced Ty3 transposition, start by similarity	0.38
YALI0F25685g		weakly similar to sp|P38355 Saccharomyces cerevisiae YBR287w unknown function, start by similarity	0.37
YALI0D15400g		similar to sp|P32451 Saccharomyces cerevisiae YGR286c BIO2 biotin synthetase, start by similarity	0.37
YALI0D12947g		weakly similar to sp|Q04746 Saccharomyces cerevisiae YMR065w KAR5 nuclear fusion protein, hypothetical start	0.37
YALI0E29381g		no similarity, hypothetical start	0.36
YALI0E28371g		similar to sp|P46675 Saccharomyces cerevisiae YLR045c STU2 suppressor of a cs tubulin mutation, start by similarity	0.35
YALI0A01826g		weakly similar to DEHA0C16203g Debaryomyces hansenii IPF 3466.1, hypothetical start	0.35
YALI0A18139g		highly similar to sp|Q07478 Saccharomyces cerevisiae YDL084w SUB2 Probable ATP-dependent RNA helicase involved in pre-mRNA splicing, start by similarity	0.34
YALI0D19866g		similar to CAGL0I08613g Candida glabrata, hypothetical start	0.33
YALI0D22957g		weakly similar to sp|Q12303 Saccharomyces cerevisiae YLR121c YPS3 GPI-anchored aspartyl protease 3 (yapsin 3), hypothetical start	0.33
YALI0C20060g		similar to sp|P41928 Yarrowia lipolytica MTP2 Metallothionein-II, start by similarity	0.33
YALI0D00231g		no similarity, hypothetical start	0.33
YALI0E27291g		similar to tr|Q96VC8 Yarrowia lipolytica Glyoxylate pathway regulator, hypothetical start	0.33
YALI0F22319g		no similarity, hypothetical start	0.32
YALI0F01320g	YlALK2	tr|O74128 Yarrowia lipolytica ALK2 (CytP450 familly), identified start	0.32
YALI0A10186g		no similarity, hypothetical start	0.31
YALI0F21109g		weakly similar to tr|CAD70735 Neurospora crassa 64C2.200 Related to putative tartrate transporter and sp|P53322 Saccharomyces cerevisiae YGR260w TNA1, hypothetical start	0.31
YALI0E03894g		some similarities with tr|Q8X051 Neurospora crassa B1K11.070, hypothetical start	0.31
YALI0B19734g		weakly similar to sp|P36097 Saccharomyces cerevisiae YKL033w similarity to hypothetical protein S. pombe singleton, start by similarity	0.30
YALI0E27115g		no similarity, hypothetical start	0.29
YALI0F22583g		no similarity, hypothetical start	0.28
YALI0C03201g		weakly similar to tr|Q9XWG2 Caenorhabditis elegans Y63D3A.7 protein, start by similarity	0.25
YALI0E04510g		similar to tr|Q9URL7 Candida albicans PTR2 Peptide transport protein, hypothetical start	0.25
YALI0B16852g		similar to DEHA0B12474g Debaryomyces hansenii IPF 10115.1, hypothetical start	0.23
YALI0E05819g		weakly similar to sp|P08640 Saccharomyces cerevisiae YIR019c STA1 extracellular	0.22

	alpha-1, 4-glucan glucosidase, hypothetical start	
YALI0A21263g	similar to sp|O93852 Candida albicans D-arabinono-1, 4-lactone oxidase, hypothetical start	0.21
YALI0D00363g	weakly similar to sp|P11636 Neurospora crassa Quinate permease, start by similarity	0.07
YALI0A21307g	similar to sp|P25297 Saccharomyces cerevisiae YML123c PHO84 high-affinity inorganic phosphate/H+ symporter, hypothetical start	0.05
YALI0A21461g	similar to sp|P36114 Saccharomyces cerevisiae YKR018c, hypothetical start	0.04
YALI0A21417g	similar to sp|O74267 Ashbya gossypii Threonine aldolase, hypothetical start	0.03
YALI0A21439g	no similarity	0.03
YALI0A21329g	weakly similar to CAGL0L02475g Candida glabrata, no start	0.01
YALI0D26642g	similar to sp|Q01961 Pichia pastoris Peroxisome assembly protein PAS10, hypothetical start	0.01

Table S5. Genes up-regulated (237) during the yeast to hyphae transition at 60 minues induced by GlNAc mediated by ZNC1.

Locus tag	Gene	Description	Fold change
YALI0F10373g		some similarities with sp|P18899 Saccharomyces cerevisiae YMR173w DDR48 heat shock protein, hypothetical start	40.82
YALI0C11165g		no similarity, hypothetical start	22.64
YALI0C09526g		weakly similar to sp|P25567 Saccharomyces cerevisiae YCL037c SRO9 La motif-containing proteins that modulate mRNA translation, hypothetical start	12.77
YALI0B11528g		no similarity	12.31
YALI0E03828g		similar to sp|P78795 Schizosaccharomyces pombe SPBC18H10.03 Probable eukaryotic translation initiation factor 3 RNA-binding subunit (eIF-3 RNA-binding subunit) (eIF3 p33) (Translation initiation factor eIF3, p33 subunit), hypothetical start	11.45
YALI0E23859g		similar to sp|P38361 Saccharomyces cerevisiae YBR296c PHO89 Na+-coupled phosphate transport protein, high affinity, hypothetical start	9.27
YALI0F18876g		some similarities with wi|NCU09365.1 Neurospora crassa NCU09365.1, hypothetical start	8.04
YALI0B04774g		weakly similar to tr|Q8R0W9 Mus musculus Hypothetical 38.2 kDa protein Fragment, hypothetical start	7.69
YALI0C11209g		no similarity, hypothetical start	7.52
YALI0B16764g		no similarity, hypothetical start	7.45
YALI0B09779g		highly similar to DEHA0G23012g Debaryomyces hansenii IPF 4913.1, start by similarity	7.10
YALI0E01584g		similar to DEHA0E11000g Debaryomyces hansenii, hypothetical start	6.49
YALI0D15224g		no similarity, hypothetical start	6.27
YALI0B18326g		no similarity	6.18
YALI0D22638g		similar to DEHA0A10406g Debaryomyces hansenii IPF 7102.1, hypothetical start	6.06
YALI0E13255g		similar to tr|Q96VB9 Candida albicans CA1911 Chaperone protein CaMsi3p, CaSSE1 heat shock protein of HSP70 family, start by similarity	5.90
YALI0F17556g		highly similar to sp|P53163 Saccharomyces cerevisiae Putative 60S ribosomal protein L7/L12 homolog, mitochondrial, hypothetical start	5.73
YALI0B09581g		no similarity, hypothetical start	5.39
YALI0E17611g		weakly similar to tr|O43119 Aspergillus niger Peptide-N4-(N-acetyl-beta-D-glucosaminyl) asparaginase amidase N, hypothetical start	5.20
YALI0B08426g		no similarity, hypothetical start	5.17
YALI0A03839g		highly similar to sp|P32481 Saccharomyces cerevisiae YER025w GCD11 translation initiation factor eIF2 gamma chain, start by similarity	5.16
YALI0E23716g		similar to sp|Q02774 Saccharomyces cerevisiae YDL212w SHR3 endoplasmatic reticulum membrane protein, hypothetical start	5.06
YALI0D01980g		similar to sp|P40579 Saccharomyces cerevisiae YIR035c similarity to human corticosteroid 11-beta-dehydrogenase, start by similarity	4.75

YALI0E26125g	tr|Q8TFK5 Yarrowia lipolytica YlCWP1 Cell wall protein, start by similarity	4.70
YALI0E21505g	weakly similar to sp|P47118 Saccharomyces cerevisiae YJR067c YAE1, start by similarity	4.67
YALI0F09537g	no similarity, possibly noncoding, hypothetical start	4.62
YALI0D21956g	similar to sp|P22214 Saccharomyces cerevisiae YLR268w SEC22 synaptobrevin (V-SNARE) singleton, start by similarity	4.58
YALI0A02178g	similar to DEHA0G23056g Debaryomyces hansenii IPF 4911.1, hypothetical start	4.52
YALI0D08690g	similar to sp|P50136 Mus musculus 2-oxoisovalerate dehydrogenase alpha subunit, mitochondrial precursor, start by similarity	4.49
YALI0B09999g	weakly similar to tr|Q8ES18 Oceanobacillus iheyensis 2-nitropropane dioxygenase, hypothetical start	4.44
YALI0D26345g	similar to tr|Q12006 Saccharomyces cerevisiae YOL003c, hypothetical start	4.43
YALI0E30349g	weakly similar to DEHA0F21252g Debaryomyces hansenii IPF 6452.1, hypothetical start	4.40
YALI0E12595g	similar to tr|Q9V9A7 Drosophila melanogaster Putative propionyl-CoA carboxylase beta chain, mitochondrial precursor (EC 6.4.1.3) (PCCase beta subunit) (Propanoyl-CoA:carbon dioxide ligase beta subunit), hypothetical start	4.33
YALI0D12144g	similar to sp|Q06142 Saccharomyces cerevisiae YLR347c KAP95 karyopherin-beta singleton, start by similarity	4.26
YALI0B10021g	similar to sp|P38861 Saccharomyces cerevisiae YHR170w NMD3 nonsense-mediated mRNA decay protein singleton, hypothetical start	4.26
YALI0C06083g	similar to tr|Q9Y8A4 Aspergillus oryzae 5-aminolevulinic acid synthase, start by similarity	4.23
YALI0E25696g	similar to tr|Q9HGM9 Schizosaccharomyces pombe DNAJ domain protein, hypothetical start	4.22
YALI0E05313g	similar to tr|Q08777 Saccharomyces cerevisiae YOR306c, hypothetical start	4.22
YALI0F23485g	some similarities with wi|NCU02191.1 Neurospora crassa NCU02191.1 hypothetical protein, hypothetical start	4.14
YALI0C08052g	similar to sp|P10962 Saccharomyces cerevisiae YAL025c MAK16 nuclear viral propagation protein, hypothetical start	4.12
YALI0F02805g	highly similar to sp|P19882 Saccharomyces cerevisiae YLR259c HSP60 heat shock protein - chaperone, mitochondrial, start by similarity	4.05
YALI0E01100g	similar to sp|P21304 Saccharomyces cerevisiae YLR196w PWP1, start by similarity	4.05
YALI0D14102g	similar to sp|P40018 Saccharomyces cerevisiae YER029c SMB1 associated with U1 snRNP as part of the Sm-core that is common to all spliceosomal snRNPs, start by similarity	4.02
YALI0F16115g	similar to DEHA0F10681g Debaryomyces hansenii IPF 8292.1, start by similarity	4.00
YALI0B08052g	similar to DEHA0A06347g Debaryomyces hansenii, start by similarity	3.97
YALI0A00594g	similar to sp|P32527 Saccharomyces cerevisiae YGR285c ZUO1 zuotin, a putative Z-DNA binding protein, start by similarity	3.95
YALI0F24409g	some similarities with sp|P53721 Saccharomyces cerevisiae YNR018w, hypothetical start	3.87

YALI0E18854g	some similarities with sp|P87058 Schizosaccharomyces pombe La protein homolog (La ribonucleoprotein) (La autoantigen homolog), hypothetical start	3.79
YALI0B17930g	similar to sp|Q12325 Saccharomyces cerevisiae YLR092w SEL2 sulfate transporter P4.33.f3.1 or sp|P38359 Saccharomyces cerevisiae YBR294w SUL1 high-affinity sulfate transport protein P4.33.f3.1, hypothetical start	3.79
YALI0E21626g	similar to sp|P47122 Saccharomyces cerevisiae YJR072c, hypothetical start	3.79
YALI0B15598g	highly similar to sp|P38720 Saccharomyces cerevisiae YHR183w GND1 6-phosphogluconate dehydrogenase P2.360.f2.1 or sp|P53319 Saccharomyces cerevisiae YGR256w GND2 phosphogluconate dehydrogenase P2.360.f2.1, hypothetical start	3.75
YALI0C15092g	some similarities with sp|P41913 Saccharomyces cerevisiae GDS1 protein, hypothetical start	3.73
YALI0A18205g	similar to sp|P32905 Saccharomyces cerevisiae YGR214w NAB1A 40S ribosomal protein p40 homolog A, hypothetical start	3.71
YALI0F08701g	similar to sp|P25087 Saccharomyces cerevisiae YML008c ERG6 S-adenosyl-methionine and wi|NCU03006.1 Neurospora crassa, start by similarity	3.69
YALI0D23683g	similar to sp|P12695 Saccharomyces cerevisiae Dihydrolipoamide acetyltransferase component of pyruvate dehydrogenase complex, mitochondrial precursor (PDC-E2), hypothetical start	3.64
YALI0E30877g	weakly similar to sp|P34167 Saccharomyces cerevisiae YPR163c TIF3 translation initiation factor eIF4B singleton, start by similarity	3.58
YALI0B07051g	no similarity, hypothetical start	3.58
YALI0E16280g	weakly similar to tr|Q04177 Saccharomyces cerevisiae YDR398W snoRNA binding activity, start by similarity	3.55
YALI0F09427g	weakly similar to ca|CA3280|IPF6269.3 Candida albicans, hypothetical start	3.54
YALI0F22825g	similar to sp|Q12358 Saccharomyces cerevisiae YLL057c, start by similarity	3.43
YALI0E14707g	no similarity, hypothetical start	3.43
YALI0E08844g	weakly similar to CAGL0I06600g Candida glabrata, hypothetical start	3.43
YALI0A17985g	weakly similar to sp|P14906 Saccharomyces cerevisiae YOR254c SEC63 ER protein-translocation complex subunit NPL1, hypothetical start	3.42
YALI0F24893g	similar to sp|P00931 Saccharomyces cerevisiae YGL026c TRP5 tryptophan synthase, start by similarity	3.38
YALI0F27885g	weakly similar to sp|P40433 Saccharomyces cerevisiae YIL107c PFK26 6-phosphofructose-2-kinase, hypothetical start	3.37
YALI0E10549g	similar to CAGL0F08481g Candida glabrata, hypothetical start	3.37
YALI0D20768g	similar to sp|P09624 Saccharomyces cerevisiae YFL018c LPD1 dihydrolipoamide dehydrogenase precursor P3. 18.f3.1, hypothetical start	3.35
YALI0D18898g	no similarity, hypothetical start	3.33
YALI0E31713g	weakly similar to wi|NCU08741.1 Neurospora crassa NCU08741.1, hypothetical start	3.31

YALI0D15708g	similar to sp|P45954 Homo sapiens Acyl-CoA dehydrogenase, short/branched chain specific, mitochondrial precursor, start by similarity	3.29
YALI0C09031g	no similarity, hypothetical start	3.27
YALI0C09328g	similar to tr|Q12118 Saccharomyces cerevisiae YOR007c SGT2 similarity to protein phosphatases, start by similarity	3.27
YALI0E27940g	highly similar to sp|Q12522 Saccharomyces cerevisiae YPR016c TIF6 translation initiation factor 6 (eIF6), start by similarity	3.27
YALI0F08393g	similar to sp|Q02642 Saccharomyces cerevisiae YPL037c DNA-binding enhancer protein, start by similarity	3.25
YALI0F24739g	highly similar to sp|P05736 Saccharomyces cerevisiae YIL018w RPL5A 60S large subunit ribosomal protein L8.e [INTRON], start by similarity	3.23
YALI0F00462g	similar to sp|O43315 Homo sapiens Aquaporin 9 (Small solute channel 1), hypothetical start	3.23
YALI0C07843g	similar to tr|Q872S8 Neurospora crassa B8B8.060 Hypothetical protein, hypothetical start	3.22
YALI0B01386g	similar to sp|P32795 Saccharomyces cerevisiae YME1 protease of the SEC18/CDC48/PAS1 family of ATPases (AAA), hypothetical start	3.22
YALI0F12111g	similar to sp|P40564 Saccharomyces cerevisiae YIR004w and DEHA0E03685g Debaryomyces hansenii, start by similarity	3.21
YALI0E17919g	similar to sp|P48525 Saccharomyces cerevisiae YOL033w MSE1 glutamyl-tRNA synthetase, mitochondrial singleton, hypothetical start	3.16
YALI0B07755g	weakly similar to tr|Q9VXZ8 Drosophila melanogaster CG9009 protein (BCDNA.GH02901), hypothetical start	3.15
YALI0C19624g	similar to sp|P38071 Saccharomyces cerevisiae YBR026c (MRF1) mitochondrial respiratory function protein, hypothetical start	3.12
YALI0E31735g	no similarity	3.12
YALI0C11341g	highly similar to tr|Q12230 Saccharomyces cerevisiae Hypothetical 38.1 kDa protein YPL004C, hypothetical start	3.11
YALI0E22352g	highly similar to sp|P26321 Saccharomyces cerevisiae YPL131w RPL1 60S large subunit ribosomal protein L5.e, start by similarity	3.09
YALI0E31603g	similar to KLLA0B14839g Kluyveromyces lactis IPF 6866.1, hypothetical start	3.09
YALI0B21868g	weakly similar to tr|Q20683 Caenorhabditis elegans F52H3.5 protein, start by similarity	3.07
YALI0F28831g	similar to DEHA0C17006g Debaryomyces hansenii, hypothetical start	3.06
YALI0D03531g	similar to sp|Q01476 Saccharomyces cerevisiae YOR124c UBP2 ubiquitin-specific proteinase, hypothetical start	3.04
YALI0F27049g	no similarity	3.02
YALI0D27324g	similar to tr|O74907 Schizosaccharomyces pombe Hypothetical, hypothetical start	3.02
YALI0B03938g	no similarity, hypothetical start	3.01
YALI0D18106g	similar to CA5008|IPF12577 Candida albicans IPF12577 unknown function, hypothetical	3.01

		start	
YALI0E06193g		similar to sp|P24521 Saccharomyces cerevisiae YMR220w ERG8 phosphomevalonate kinase, start by similarity	3.00
YALI0E02882g		similar to DEHA0G25817g Debaryomyces hansenii and sp|P45976 Saccharomyces cerevisiae YJR093c FIP1 component of pre-mRNA polyadenylation factor PF I, possible transmembrane segment, start by similarity	3.00
YALI0E24673g		weakly similar to sp|P53301 Saccharomyces cerevisiae YGR189c CRH1, hypothetical start	3.00
YALI0D08734g		weakly similar to sp|P06843 Saccharomyces cerevisiae YER161c SPT2 multifunctional HMG-like chromatin protein, hypothetical start	3.00
YALI0E01210g		no similarity, hypothetical start	3.00
YALI0E07425g		similar to tr|Q12754 Saccharomyces cerevisiae YPL012w hypothetical protein, hypothetical start	3.00
YALI0E19965g		no similarity, hypothetical start	2.99
YALI0D17116g		highly similar to sp|Q12460 Saccharomyces cerevisiae YLR197w SIK1 involved in pre-rRNA processing, start by similarity	2.99
YALI0F21010g	YlADE1	sp|Q99148 Yarrowia lipolytica Bifunctional purine biosynthetic protein ADE1, identified start	2.97
YALI0B21516g		similar to sp|P53759 Saccharomyces cerevisiae YML080w similarity to A.brasilense nifR3 protein P3.77.f3. 1, start by similarity	2.96
YALI0B12474g		similar to sp|P38788 Saccharomyces cerevisiae YHR064c PDR13 regulator protein involved in pleiotropic drug resistance, start by similarity	2.94
YALI0C24101g		highly similar to sp|P11154 Saccharomyces cerevisiae YGL062w PYC1 pyruvate carboxylase 1, hypothetical start	2.92
YALI0B01980g		some similarities with sp|P87250 Kluyveromyces lactis mitochondrial replication protein MTF1, hypothetical start	2.91
YALI0E00550g		similar to sp|P36105 Saccharomyces cerevisiae YKL006w RPL14A ribosomal protein or sp|P38754 Saccharomyces cerevisiae YHL001w RPL14B ribosomal protein, hypothetical start	2.90
YALI0B21076g		highly similar to sp|P02406 Saccharomyces cerevisiae YGL103w CYH2 60S large subunit ribosomal protein L27a, hypothetical start	2.90
YALI0C24255g		similar to sp|Q03148 Saccharomyces cerevisiae YMR096w SNZ1 stationary phase protein, start by similarity	2.89
YALI0F08657g		weakly similar to tr|Q06679 Saccharomyces cerevisiae YDR324c, start by similarity	2.88
YALI0C04004g		highly similar to DEHA0B15224g Debaryomyces hansenii IPF 475.1, start by similarity	2.88
YALI0E17325g		some similarities with sp|P38314 Saccharomyces cerevisiae YBR214w SDS24, hypothetical start	2.83
YALI0F10153g		similar to sp|P33313 Saccharomyces cerevisiae YBR155w CNS1 and DEHA0D09999g	2.83

		Debaryomyces hansenii, hypothetical start	
YALI0C21846g		highly similar to sp|Q08965 Saccharomyces cerevisiae YPL217c BMS1 Ribosome biogenesis protein, start by similarity	2.83
YALI0E07898g		weakly similar to sp|P35190 Saccharomyces cerevisiae YGL215w CLG1 cyclin-like protein, hypothetical start	2.83
YALI0C19682g		similar to CA0795|IPF15641 Candida albicans IPF15641 unknown function, start by similarity	2.81
YALI0D01892g		similar to sp|P53064 Saccharomyces cerevisiae YGL244w RTF1 regulates DNA binding properties of TBP, start by similarity	2.79
YALI0B05434g		no similarity, hypothetical start	2.79
YALI0D24343g		weakly similar to sp|P47045 Saccharomyces cerevisiae Hypothetical 54.2 kDa protein in BTN1-PEP8 intergenic region, hypothetical start	2.78
YALI0D11110g		similar to sp|P00937 Saccharomyces cerevisiae Anthranilate synthase component II (EC 4.1.3.27) [Includes: Glutamine amidotransferase; Indole-3-glycerol phosphate synthase (EC 4.1.1.48) (PRAI)], start by similarity	2.77
YALI0A20614g		similar to tr|Q06506 Saccharomyces cerevisiae of the beta-transducin family of guanine nucleotide-binding, hypothetical start	2.77
YALI0E26917g		no similarity, hypothetical start	2.76
YALI0C02717g		similar to KLLA0E08767g Kluyveromyces lactis, start by similarity	2.75
YALI0D05159g		similar to sp|P38174 Saccharomyces cerevisiae YBL091c MAP2 methionine aminopeptidase, isoform 2, start by similarity	2.74
YALI0B00550g		weakly similar to DEHA0C09053g Debaryomyces hansenii IPF 1884.1, hypothetical start	2.73
YALI0F29205g		similar to sp|P53720 Saccharomyces cerevisiae YNR015w SMM1 tRNA dihydrouridine synthase, start by similarity	2.71
YALI0F14245g		similar to tr|O74978 Schizosaccharomyces pombe Putative RNA-binding protein, start by similarity	2.71
YALI0E08387g		highly similar to sp|P41927 Yarrowia lipolytica Metallothionein-I MTLI, start by similarity	2.70
YALI0C23452g		no similarity, hypothetical start	2.70
YALI0E05533g	YlLYC1	sp|P41929 Lysine acetyltransferase, identified start	2.70
YALI0D00693g		similar to sp|P38682 Saccharomyces cerevisiae YER122c GLO3 zinc finger protein, start by similarity	2.68
YALI0A12023g		some similarities with sp|P38637 Saccharomyces cerevisiae YIR011c STS1 required for transport of RNA15P from the cytoplasm to the nucleus, hypothetical start	2.68
YALI0B22550g		similar to tr|Q9VM58 Drosophila melanogaster CG10399 protein, start by similarity	2.67
YALI0B04180g		similar to sp|P40825 Saccharomyces cerevisiae Alanyl-tRNA synthetase, cytoplasmic (EC 6.1.1.7) (Alanine--tRNA ligase), start by similarity	2.66
YALI0E30217g		similar to sp|Q05022 Saccharomyces cerevisiae YMR229c RRP5 processing of pre-ribosomal RNA P2.264.f2.1, hypothetical start	2.65

YALI0D08668g	similar to sp|P35178 Saccharomyces cerevisiae YDR087c RRP1 involved in processing rRNA precursor species, start by similarity	2.65
YALI0B20108g	weakly similar to tr|Q05518 Saccharomyces cerevisiae Similar to S. CEREVISIAE hypothetical protein YHR097P, hypothetical start	2.64
YALI0E26686g	similar to tr|Q9HW37 Pseudomonas aeruginosa Hypothetical protein, start by similarity	2.63
YALI0D12400g	sp|P29407 Yarrowia lipolytica Phosphoglycerate kinase, identified start	2.62
YALI0B10802g	similar to tr|Q8X005 Neurospora crassa Glycine rich protein (het-COR), hypothetical start	2.62
YALI0E09691g	similar to sp|Q02608 Saccharomyces cerevisiae YPL013c, hypothetical start	2.60
YALI0B00264g	similar to sp|P32860 Saccharomyces cerevisiae YKL040c NFU1 iron homeostasis, hypothetical start	2.60
YALI0C16841g	no similarity, hypothetical start	2.59
YALI0F31251g	some similarities with tr|Q96WX5 Botrytis cinerea Bc-hch, hypothetical start	2.59
YALI0F16137g	weakly similar to sp|P53072 Saccharomyces cerevisiae YGL232w, start by similarity	2.58
YALI0B22374g	similar to sp|P32608 Saccharomyces cerevisiae Retrograde regulation protein 2, start by similarity	2.57
YALI0E21780g	similar to tr|AAO32553 Saccharomyces kluyveri AKR1, hypothetical start	2.56
YALI0C05731g	no similarity, hypothetical start	2.56
YALI0E20119g	similar to ca|CA0123|CaMXR1 Candida albicans CaMXR1 methionine sulfoxide reductase, hypothetical start	2.55
YALI0E21846g	similar to sp|P09032 Saccharomyces cerevisiae YOR260w GCD1 translation initiation factor eIF2bgamma subunit, hypothetical start	2.52
YALI0E16434g	similar to sp|P36107 Saccharomyces cerevisiae YKL004w AUR1 aureobasidin-resistance protein, hypothetical start	2.51
YALI0B01034g	highly similar to sp|P56286 Schizosaccharomyces pombe Eukaryotic translation initiation factor 2 alpha subunit (eIF-2- alpha), hypothetical start	2.50
YALI0B12716g	weakly similar to sp|P41546 Saccharomyces cerevisiae YFL031w HAC1 transcription factor singleton [INTRON], start by similarity	2.50
YALI0D10483g	no similarity, hypothetical start	2.50
YALI0E32967g	weakly similar to tr|Q97P71 Streptococcus pneumoniae SP1772 Cell wall surface anchor family protein, hypothetical start	2.49
YALI0B07425g	similar to sp|P79003 Saccharomyces pastorianus RER1 protein (Retention of ER proteins 1), start by similarity	2.46
YALI0B02222g	similar to sp|Q12184 Saccharomyces cerevisiae YPL252c Adrenodoxin homolog, mitochondrial precursor (Mitochondrial ferredoxin), hypothetical start	2.46
YALI0E14124g	similar to sp|P42847 Saccharomyces cerevisiae YNL306w MRPS18 ribosomal protein of the small subunit, mitochondrial, start by similarity	2.46
YALI0C01441g	weakly similar to tr|O42997 Schizosaccharomyces pombe Hypothetical 33.6 kDa protein, frameshift	2.44

YALI0C18381g	some similarities with tr|Q9UT57 Schizosaccharomyces pombe WD domian, G-beta repeat protein, hypothetical start	2.44
YALI0F04664g	similar to sp|P29469 Saccharomyces cerevisiae YBL023c MCM2 member of the MCM2P, MCM3P, CDC46P family, start by similarity	2.43
YALI0D11792g	similar to CA0750|IPF6284 Candida albicans IPF6284 unknown function, hypothetical start	2.43
YALI0F05808g	highly similar to sp|P05319 Saccharomyces cerevisiae YOL039w RPLA2 acidic ribosomal protein P2.beta, start by similarity	2.42
YALI0B12276g	similar to tr|Q06106 Saccharomyces cerevisiae Hypothetical 101.1 kDa protein P8283.19, start by similarity	2.42
YALI0B09471g	similar to tr|O60121 Schizosaccharomyces pombe Stomatin family protein, hypothetical start	2.42
YALI0C03443g	weakly similar to tr|Q89C37 Bradyrhizobium japonicum Blr7961 protein, hypothetical start	2.41
YALI0B16412g	similar to sp|P40445 Saccharomyces cerevisiae YIL166c similarity to allantoate permease DAL5P P10.2.f5.1, hypothetical start	2.41
YALI0D05005g	similar to sp|P34228 Saccharomyces cerevisiae YBL066c SEF1 putative transcription factor, start by similarity	2.41
YALI0D17424g	similar to sp|P32906 Saccharomyces cerevisiae YJR131w MNS1 ER alpha1, 2-mannosidase, start by similarity	2.40
YALI0E25003g	no similarity, possibly noncoding, hypothetical start	2.39
YALI0D06303g	highly similar to sp|P28241 Saccharomyces cerevisiae YOR136w IDH2 isocitrate dehydrogenase, hypothetical start	2.39
YALI0A10615g	similar to sp|P39925 Saccharomyces cerevisiae YER017c AFG3 protease of the SEC18/CDC48/PAS1 family of ATPases (AAA), start by similarity	2.39
YALI0D27170g	similar to sp|O60122 Schizosaccharomyces pombe Anthranilate phosphoribosyltransferase, hypothetical start	2.39
YALI0B11594g	similar to tr|Q96UQ4 Aspergillus niger Aminopeptidase B, hypothetical start	2.39
YALI0C06798g	highly similar to tr|Q9P5P3 Neurospora crassa Probable branching enzyme (be1), hypothetical start	2.38
YALI0D01265g	similar to sp|P38891 Saccharomyces cerevisiae YHR208w BAT1 branched chain amino acid aminotransferase, mitochondrial precursor, start by similarity	2.38
YALI0D10329g	weakly similar to tr|O42662 Schizosaccharomyces pombe Possible DNA methylase, hypothetical start	2.37
YALI0C03355g	highly similar to KLLA0D03608g Kluyveromyces lactis IPF 5171.1, start by similarity	2.36
YALI0F24387g	highly similar to sp|P02992 Saccharomyces cerevisiae YOR187w TUF1 translation elongation factor TU, mitochondrial, hypothetical start	2.34
YALI0B02574g	similar to sp|P39925 Saccharomyces cerevisiae Mitochondrial respiratory chain complexes assembly protein AFG3 (EC 3.4.24.-) (TAT-binding homolog 10), hypothetical	2.34

	start	
YALI0D20570g	highly similar to sp|P39077 Saccharomyces cerevisiae YJL014w CCT3 chaperonin of the TCP1 ring complex, cytosolic, hypothetical start	2.33
YALI0F23221g	similar to sp|P16120 Saccharomyces cerevisiae Threonine synthase (EC 4.2.3.1) (TS) YCR053w (o-p-homoserine p-lyase), start by similarity	2.33
YALI0B08118g	weakly similar to KLLA0C06072g Kluyveromyces lactis, hypothetical start	2.33
YALI0D26488g	similar to tr|Q9C0V7 Schizosaccharomyces pombe Putative arylsulfatase, hypothetical start	2.32
YALI0E10439g	similar to DEHA0C15400g Debaryomyces hansenii, hypothetical start	2.32
YALI0E01540g	no similarity, hypothetical start	2.32
YALI0F13453g	highly similar to sp|P17423 Saccharomyces cerevisiae YHR025w THR1 homoserine kinase and KLLA0E11528g Kluyveromyces lactis and CAGL0J00649g Candida glabrata, start by similarity	2.31
YALI0E17303g	weakly similar to tr|CAD60707 Podospora anserina CAD60707, hypothetical start	2.30
YALI0D23859g	highly similar to sp|P53941 Saccharomyces cerevisiae U3 small nucleolar ribonucleoprotein protein IMP4, start by similarity	2.30
YALI0E25025g	similar to sp|P05735 Saccharomyces cerevisiae YBR084ca RPL19B 60S large subunit ribosomal protein L19, hypothetical start	2.30
YALI0C16929g	similar to tr|Q96X51 Pholiota nameko and tr|O94342 Schizosaccharomyces pombe Metabolite transport protein, start by similarity	2.29
YALI0F23903g	similar to sp|P30606 Saccharomyces cerevisiae YOL103w ITR2 myo-inositol permease, hypothetical start	2.28
YALI0F10175g	no similarity, hypothetical start	2.28
YALI0F17204g	weakly similar to tr|Q9C228 Neurospora crassa Related to CSI2 protein, hypothetical start	2.27
YALI0B06116g	similar to sp|P38120 Saccharomyces cerevisiae Probable 40S ribosomal protein S9, mitochondrial precursor, hypothetical start	2.27
YALI0B09991g	some similarities with DEHA0D15972g Debaryomyces hansenii IPF 2966.1, hypothetical start	2.27
YALI0C20141g	similar to tr|Q05776 Saccharomyces cerevisiae YLR193c Similar to MSF1 protein, hypothetical start	2.27
YALI0B11946g	some similarities with tr|AAH43775 Xenopus laevis, hypothetical start	2.27
YALI0B12342g	weakly similar to sp|P40578 Saccharomyces cerevisiae MGA2 protein, hypothetical start	2.27
YALI0B22066g	highly similar to wi|NCU01680.1 Neurospora crassa NCU01680.1 plasma membrane ATPase (proton pump), hypothetical start	2.27
YALI0A02651g	similar to sp|P19968 Neurospora crassa NADH-ubiquinone oxidoreductase 21.3 kDa subunit (EC 1.6.5. 3) (EC 1.6.99.3), hypothetical start	2.27
YALI0F04730g	tr|Q8TFK3 Yarrowia lipolytica GTP-binding protein, identified start	2.26
YALI0E20163g	weakly similar to tr|Q9C0N5 Candida albicans CaNAG2 N-acetyl-glucosamine-6-	2.26

	phosphate deacetylase, hypothetical start	
YALI0F30393g	similar to sp|P25586 Saccharomyces cerevisiae YCL059c KRR1 unknown function, start by similarity	2.25
YALI0D13068g	similar to tr|Q08004 Saccharomyces cerevisiae Chromosome XII reading frame ORF YLR074C, zinc finger protein, start by similarity	2.25
YALI0F09834g	similar to sp|P32565 Saccharomyces cerevisiae YIL075c SEN3 26S proteasome regulatory subunit and DEHA0B11220g Debaryomyces hansenii, start by similarity	2.25
YALI0A03773g	similar to sp|P53883 Saccharomyces cerevisiae YNL175c NOP13, start by similarity	2.25
YALI0B01782g	similar to tr|Q96VT2 Aspergillus niger prolyl aminopeptidase A (EC 3.4.11.5), start by similarity	2.25
YALI0F00396g	similar to sp|Q10740 Saccharomyces cerevisiae YNL045w, hypothetical start	2.24
YALI0F06468g	similar to sp|P33330 Saccharomyces cerevisiae YOR184w SER1 phosphoserine transaminase, start by similarity	2.24
YALI0F05544g	highly similar wi|NCU01949.1 Neurospora crassa NCU01949.1 and sp|P05755 Saccharomyces cerevisiae YBR189w SUP46 ribosomal protein, start by similarity	2.24
YALI0F20372g	similar to tr|Q12136 Saccharomyces cerevisiae YDL153c SAS10 involved in silencing, hypothetical start	2.22
YALI0F31383g	similar to sp|P40160 Saccharomyces cerevisiae YNL207w, start by similarity	2.22
YALI0D25322g	no similarity, hypothetical start	2.22
YALI0D21934g	weakly similar to sp|Q02204 Saccharomyces cerevisiae YKR006c MRPL13 ribosomal protein YmL13, mitochondrial singleton, hypothetical start	2.21
YALI0C21362g	similar to sp|P39676 Saccharomyces cerevisiae YGR234w YHB1 flavohemoglobin, start by similarity	2.21
YALI0F24321g	similar to sp|P32502 Saccharomyces cerevisiae Translation initiation factor eIF-2B beta subunit (eIF-2B GDP-GTP exchange factor) (Guanine nucleotide exchange factor subunit GCD7) (GCD complex subunit GCD7) YLR291c, hypothetical start	2.20
YALI0F31053g	similar to sp|P32333 Saccharomyces cerevisiae YPL082c MOT1 transcriptional accessory protein, start by similarity	2.20
YALI0B16280g	similar to tr|Q9UV33 Ascobolus immersus Histone H1, hypothetical start	2.17
YALI0C21065g	some similarities with sp|P47011 Saccharomyces cerevisiae YJL137c GLG2 self-glucosylating initiator of glycogen synthesis P2.283.f2.1, hypothetical start	2.17
YALI0D13838g	weakly similar to sp|P45978 Saccharomyces cerevisiae YPR129w SCD6 suppressor of clathrin deficiency, start by similarity	2.17
YALI0F13475g	similar to DEHA0A13563g Debaryomyces hansenii IPF 7368.1, hypothetical start	2.16
YALI0C18645g	similar to tr|Q04052 Saccharomyces cerevisiae YDR421w positive transcription regulator of ARO9 and ARO10, hypothetical start	2.16
YALI0C14630g	no similarity, hypothetical start	2.16
YALI0B11572g	weakly similar to sp|Q02256 Saccharomyces cerevisiae YIR026c YVH1 protein tyrosine	2.15

	phosphatase singleton, hypothetical start	
YALI0F03498g	similar to sp|P50276 Saccharomyces cerevisiae YGR055w MUP1 high affinity methionine permease, hypothetical start	2.14
YALI0C19448g	similar to sp|P22135 Saccharomyces cerevisiae YJL180c ATP12 F1F0-ATPase complex assembly protein, hypothetical start	2.13
YALI0E26048g	similar to DEHA0D12364g Debaryomyces hansenii, hypothetical start	2.12
YALI0D06842g	similar to sp|P47089 Saccharomyces cerevisiae YJR014w Density-regulated protein homolog, start by similarity	2.12
YALI0E11891g	weakly similar to sp|P25710 Neurospora crassa NADH-ubiquinone oxidoreductase 21.3 kDa subunit, no start	2.11
YALI0E21241g	similar to DEHA0D08217g Debaryomyces hansenii, hypothetical start	2.11
YALI0C06446g	similar to sp|P46681 Saccharomyces cerevisiae Actin interacting protein 2, hypothetical start	2.10
YALI0F06226g	no similarity	2.10
YALI0B10736g	highly similar to sp|Q04013 Saccharomyces cerevisiae YMR241w YHM2 yeast suppressor gene of HM (mitochondrial histone) mutant (ABF2) singleton, hypothetical start	2.09
YALI0E12133g	similar to sp|P00445 Saccharomyces cerevisiae YJR104c SOD1 copper-zinc superoxide dismutase, start by similarity	2.08
YALI0E25608g	some similarities with tr|O14099 Schizosaccharomyces pombe Zinc finger protein, hypothetical start	2.07
YALI0B01320g	no similarity, hypothetical start	2.02

Table S6. Genes down-regulated (143) during the yeast to hyphae transition at 60 minues induced by GlNAc mediated by ZNC1.

Locus tag	Gene	Description	Fold change
YALI0A01067g		weakly similar to tr|Q08422 Saccharomyces cerevisiae Chromosome XV reading frame ORF YOR052C, hypothetical start	0.49
YALI0E18568g	YlPOT1	sp|Q05493 Yarrowia lipolytica 3-ketoacyl-CoA thiolase, peroxisomal precursor, identified start	0.49
YALI0C16995g		highly similar to sp|P10963 Saccharomyces cerevisiae YKR097w PCK1 phosphoenolpyruvate carboxykinase, hypothetical start	0.49
YALI0D19030g		weakly similar to tr|Q8J0P6 Paracoccidioides brasiliensis NADH-ubiquinone oxidoreductase, hypothetical start	0.48
YALI0C08767g		weakly similar to tr|Q28205 Bos taurus Cofactor D, involved in protein folding, hypothetical start	0.48
YALI0B14993g		weakly similar to tr|Q02950 Saccharomyces cerevisiae YPL118w MRP51 mitochondrial ribosomal protein of the small subunit, hypothetical start	0.48
YALI0C13926g		weakly similar to sp|P24870 Saccharomyces cerevisiae YDL155w CLB3 cyclin, G2/M-specific P6.2.f6.1, hypothetical start	0.48
YALI0F23551g		similar to tr|CAD60606 Podospora anserina, start by similarity	0.48
YALI0F24937g		similar to sp|P53111 Saccharomyces cerevisiae YGL157w unknown function, hypothetical start	0.47
YALI0B14102g		weakly similar to tr|Q9P7E8 Schizosaccharomyces pombe Actin binding protein with SH3 domains, start by similarity	0.47
YALI0F25707g		no similarity, hypothetical start	0.47
YALI0D13684g		some similarities with sp|P17121 Saccharomyces cerevisiae GTPase activating protein SAC7, hypothetical start	0.47
YALI0B16984g		weakly similar to sp|P01120 Saccharomyces cerevisiae YNL098c RAS2 GTP-binding protein P24.2.f4.3 or sp|P01119 Saccharomyces cerevisiae YOR101w RAS1 GTP-binding protein P24.2.f4.3, start by similarity	0.47
YALI0E06519g		weakly similar to sp|P34244 Saccharomyces cerevisiae YKL101w HSL1 ser/thr protein kinase, coupling septin ring assembly to cell cycle progression, hypothetical start	0.47
YALI0C21043g		similar to sp|O14313 Schizosaccharomyces pombe Peroxisomal membrane protein pmp20, start by similarity	0.47
YALI0D00275g		no similarity	0.46
YALI0C10450g		weakly similar to sp|Q04018 Saccharomyces cerevisiae YMR244w Hypothetical 37.4 kDa protein, hypothetical start	0.46
YALI0B13992g		weakly similar to sp|P22035 Saccharomyces cerevisiae MYB-like DNA-binding protein BAS1, start by similarity	0.46
YALI0D08536g		some similarities with sp|P40485 Saccharomyces cerevisiae YIL105c, start by similarity	0.46

YALI0B14311g	weakly similar to KLLA0B11792g Kluyveromyces lactis, hypothetical start	0.46
YALI0E01254g	similar to sp|P36605 Schizosaccharomyces pombe SPBC11B10.02C HIS3 Histidinol-phosphate aminotransferase, hypothetical start	0.46
YALI0B09273g	no similarity	0.46
YALI0F25685g	weakly similar to sp|P38355 Saccharomyces cerevisiae YBR287w unknown function, start by similarity	0.46
YALI0E07073g	highly similar to sp|P43619 Saccharomyces cerevisiae YFR047c putative nicotinate-nucleotide pyrophosphorylase [carboxylating], hypothetical start	0.46
YALI0C05621g	similar to sp|P01097 Saccharomyces cerevisiae YDL181w INH1 inhibitor of mitochondrial ATPase, start by similarity	0.46
YALI0C16148g	no similarity, hypothetical start	0.46
YALI0A04455g	no similarity, hypothetical start	0.46
YALI0D18359g	weakly similar to tr|Q8CE47 Mus musculus Hypothetical protein, start by similarity	0.45
YALI0F23441g	similar to sp|Q03557 Saccharomyces cerevisiae Probable glutamyl-tRNA(Gln) amidotransferase subunit A, mitochondrial precursor (EC 6.3.5.-) (Glu-ADT subunit A) YMR293c, hypothetical start	0.45
YALI0C14498g	highly similar to sp|P38988 Saccharomyces cerevisiae Putative mitochondrial carrier protein YHM1/SHM1, hypothetical start	0.45
YALI0C15895g	highly similar to sp|P46990 Saccharomyces cerevisiae YJL177w RPL20B 60s large subunit ribosomal protein L17.e P2.300.f2.1, start by similarity	0.45
YALI0C24145g	some similarities with tr|Q9HGM4 Schizosaccharomyces pombe Hypothetical 28.8 kDa protein, hypothetical start	0.45
YALI0C12100g	similar to DEHA0C00484g Debaryomyces hansenii IPF 2562.1, hypothetical start	0.45
YALI0E03586g	weakly similar to wi|NCU01320.1 Neurospora crassa NCU01320.1, hypothetical start	0.45
YALI0D19866g	similar to CAGL0I08613g Candida glabrata, hypothetical start	0.45
YALI0D05621g	similar to sp|Q04895 Saccharomyces cerevisiae YIR028w DAL4 allantoin permease, start by similarity	0.44
YALI0B00154g	no similarity, hypothetical start	0.44
YALI0F04103g	similar to sp|P04039 Saccharomyces cerevisiae YLR395c COX8 cytochrome-c oxidase chain VIII, hypothetical start	0.44
YALI0A17020g	no similarity, hypothetical start	0.44
YALI0B05104g	similar to tr|Q8J2N2 Aspergillus sojae Leucine aminopeptidase and to YDR415c Saccharomyces cerevisiae YDR415c strong similarity to bacterial leucyl aminopeptidase, hypothetical start	0.44
YALI0C15939g	no similarity, hypothetical start	0.44
YALI0E26026g	similar to DEHA0D12386g Debaryomyces hansenii, start by similarity	0.44
YALI0E06105g	no similarity, hypothetical start	0.43
YALI0E00154g	similar to sp|P38903 Saccharomyces cerevisiae YOR014w RTS1 potential regulatory	0.43

		subunit of protein phosphatase 2A, possible transmembrane segment, start by similarity	
YALI0F14333g		similar to DEHA0D13618g Debaryomyces hansenii IPF 3158.1, start by similarity	0.43
YALI0B11242g		weakly similar to sp|Q09910 Schizosaccharomyces pombe Hypothetical protein C30D11.11 in chromosome I, hypothetical start	0.43
YALI0F27159g		some similarities with sp|P47042 Saccharomyces cerevisiae YJL057c IKS1 probable serine/threonine-protein kinase, hypothetical start	0.43
YALI0C08613g		no similarity	0.43
YALI0B22198g		weakly similar to DEHA0E03333g Debaryomyces hansenii IPF 5576.1, start by similarity	0.43
YALI0E29227g		similar to wi|NCU01253.1 Neurospora crassa NCU01253. 1 predicted protein (3246 - 1843), hypothetical start	0.42
YALI0B08272g		no similarity	0.42
YALI0E25091g		similar to DEHA0G19030g Debaryomyces hansenii, hypothetical start	0.42
YALI0C18293g		weakly similar to tr|Q9N4S7 Caenorhabditis elegans Y51B11A.1 protein, hypothetical start	0.42
YALI0C05885g		similar to tr|Q8S564 Glycine max 4-coumarate:coenzyme A ligase (EC 6.2.1.12), hypothetical start	0.42
YALI0B21230g		similar to tr|Q8J289 Kluyveromyces lactis YGL104C, start by similarity	0.42
YALI0E34045g		similar to tr|Q8X8M5 Escherichia coli Orf, hypothetical protein, hypothetical start	0.42
YALI0E24651g		some similarities with DEHA0C03817g Debaryomyces hansenii, hypothetical start	0.41
YALI0F00242g		weakly similar to DEHA0G02002g Debaryomyces hansenii IPF 937.1, start by similarity	0.41
YALI0F22429g		no similarity, hypothetical start	0.41
YALI0C23034g		some similarities with tr|Q9UR39 Schizosaccharomyces pombe HST4P, hypothetical start	0.41
YALI0C06171g		similar to tr|Q884Q9 Pseudomonas syringae Oxidoreductase zinc-binding, hypothetical start	0.41
YALI0E14927g		weakly similar to KLLA0E20141g Kluyveromyces lactis, hypothetical start	0.40
YALI0E23474g	YlALK3	tr|O74129 Yarrowia lipolytica, identified start	0.40
YALI0D10043g		similar to tr|Q86ZH9 Neurospora crassa 64C2.200 putative tartrate transporter, hypothetical start	0.40
YALI0D24365g		weakly similar to sp|P05790 Bombyx mori Fibroin heavy chain precursor (Fib-H) (H-fibroin), hypothetical start	0.40
YALI0D05181g		no similarity, hypothetical start	0.40
YALI0C20265g		similar to sp|P33302 Saccharomyces cerevisiae YOR153w PDR5 pleiotropic drug resistance protein, start by similarity	0.40
YALI0F31229g		no similarity, hypothetical start	0.40
YALI0F16335g		some similarities with sp|P23179 Saccharomyces cerevisiae YHL022c SPO11 catalytic subunit of the meiotic double strand break transesterase, hypothetical start	0.40
YALI0B20636g		weakly similar to tr|Q89QU1 Bradyrhizobium japonicum Bll3033 protein, hypothetical start	0.40
YALI0B21318g		no similarity, hypothetical start	0.40

YALI0C22836g		some similarities with tr|Q8TFK5 Yarrowia lipolytica Cell wall protein, hypothetical start	0.39
YALI0E10329g		weakly similar to tr|Q08760 Saccharomyces cerevisiae YOR301w RAX1 similarity to S. pombe SPAC23G3. 05c, hypothetical start	0.39
YALI0A02423g		sp|P41928 Yarrowia lipolytica MT2_YARLI Metallothionein-II (MT-II), identified start	0.39
YALI0D12661g		similar to sp|Q9WU19 Mus musculus Hydroxyacid oxidase 1 (EC 1.1.3.15) (HAOX1), start by similarity	0.38
YALI0C13244g		no similarity, hypothetical start	0.38
YALI0B08206g	YlCRF1	sp|P45815 Yarrowia lipolytica Copper resistance protein CRF1, identified start	0.38
YALI0E24387g		no similarity, hypothetical start	0.38
YALI0C18491g		similar to tr|Q06593 Saccharomyces cerevisiae YPR194C, hypothetical start	0.38
YALI0B16632g		no similarity, hypothetical start	0.38
YALI0D16423g		some similarities with tr|Q03362 Saccharomyces cerevisiae YDR476c hypothetical protein, hypothetical start	0.38
YALI0B21450g		similar to sp|Q12127 Saccharomyces cerevisiae YLR110c strong similarity to FLO1P, start by similarity	0.38
YALI0D16687g		weakly similar to tr|Q9FY91 Arabidopsis thaliana SIR2-family protein, hypothetical start	0.38
YALI0A09383g		weakly similar to tr|Q9P6J0 Saccharomyces cerevisiae YGR260w TNA1 related to allantoate transport protein, start by similarity	0.38
YALI0E33627g	YlRIM9	tr|Q7Z8R5 Yarrowia lipolytica Rim9/PalI protein, involved in pH sensing, mating and meiosis, identified start	0.37
YALI0E30327g		weakly similar to tr|Q9C0W5 Schizosaccharomyces pombe SPBC800.14C Hypothetical 17.7 kDa protein, hypothetical start	0.37
YALI0D03949g		highly similar to tr|Q8WZU1 Neurospora crassa Hypothetical protein B8J24.130, start by similarity	0.37
YALI0D15862g		weakly similar to sp|P40317 Saccharomyces cerevisiae YDR006c SOK1 high copy suppressor of a cyclic AMP-dependent protein kinase, hypothetical start	0.37
YALI0D19338g		no similarity	0.37
YALI0C20405g		similar to sp|P10768 Homo sapiens Esterase D (EC 3. 1.1.1), hypothetical start	0.37
YALI0B14553g		similar to sp|P32829 Saccharomyces cerevisiae YMR224c MRE11 DNA repair and meiotic recombination protein singleton, start by similarity	0.37
YALI0A19910g		similar to sp|P47137 Saccharomyces cerevisiae Probable oxidoreductase YJR096W, hypothetical start	0.36
YALI0C15488g		highly similar to DEHA0D20427g Debaryomyces hansenii IPF 2659.1, start by similarity	0.35
YALI0A20988g		similar to CA5146|CaYMC2 Candida albicans CaYMC2 Carnitine/acylcarnitine translocase, hypothetical start	0.35
YALI0F22319g		no similarity, hypothetical start	0.35
YALI0E14729g		similar to sp|P33302 Saccharomyces cerevisiae YOR153w PDR5 pleiotropic drug resistance protein (YlABC1 Yarrowia lipolytica ABC transporter), hypothetical start	0.35

YALI0F07975g	similar to DEHA0G24398g Debaryomyces hansenii, hypothetical start	0.35
YALI0A21263g	similar to sp|O93852 Candida albicans D-arabinono-1, 4-lactone oxidase, hypothetical start	0.35
YALI0B05346g	similar to DEHA0E22990g Debaryomyces hansenii IPF 11107.1, start by similarity	0.34
YALI0E34749g	similar to DEHA0B16379g Debaryomyces hansenii IPF 367.1, start by similarity	0.34
YALI0E14509g	similar to DEHA0C00902g Debaryomyces hansenii, start by similarity	0.34
YALI0D15664g	weakly similar to sp|P38867 Saccharomyces cerevisiae YHR177w, start by similarity	0.34
YALI0C16951g	similar to tr|Q96X51 Pholiota nameko Probable metabolite transporter, start by similarity	0.34
YALI0B22506g	highly similar to tr|Q9Y790 Mycosphaerella graminicola NAD-dependent formate dehydrogenase (EC 1.2.1. 2), start by similarity	0.34
YALI0F00990g	no similarity, hypothetical start	0.34
YALI0B07007g	similar to tr|Q9Y763 Phanerochaete chrysosporium 1, 4-benzoquinone reductase, start by similarity	0.34
YALI0E11517g	weakly similar to tr|Q8TFK5 Yarrowia lipolytica CWP1 Cell wall protein, start by similarity	0.33
YALI0E29359g	weakly similar to tr|Q08921 Saccharomyces cerevisiae YPL180w SHD7, hypothetical start	0.33
YALI0E00264g	similar to sp|P46367 Saccharomyces cerevisiae YOR374w ALD4 aldehyde dehydrogenase, mitochondrial, possible transmembrane segment, hypothetical start	0.33
YALI0F25333g	similar to sp|P27614 Saccharomyces cerevisiae YJL172w CPS1 Gly-X carboxypeptidase YSCS precursor, hypothetical start	0.33
YALI0B00748g	no similarity, hypothetical start	0.32
YALI0E05907g	weakly similar to tr|Q9P5J4 Neurospora crassa B23L21.350, start by similarity	0.32
YALI0E25982g	highly similar to tr|O74127 Yarrowia lipolytica ALK1, start by similarity	0.31
YALI0E27247g	similar to sp|P53322 Saccharomyces cerevisiae YGR260w TNA1, hypothetical start	0.30
YALI0B05522g	similar to tr|Q96TK5 Coccidioides immitis Aspartyl aminopeptidase, start by similarity	0.30
YALI0D22660g	no similarity, hypothetical start	0.30
YALI0F03432g	weakly similar to DEHA0E09603g Debaryomyces hansenii IPF 12160.1, hypothetical start	0.30
YALI0D00154g	no similarity, hypothetical start	0.29
YALI0B20416g	similar to tr|O94524 Schizosaccharomyces pombe Protein with glutathione S transferase domain, start by similarity	0.28
YALI0E31889g	weakly similar to tr|Q8EL71 Oceanobacillus iheyensis Hypothetical conserved protein, hypothetical start	0.28
YALI0B08008g	some similarities with tr|CAD71013 Neurospora crassa 20H10.300, hypothetical start	0.28
YALI0F29249g	weakly similar to tr|Q9P5P9 Neurospora crassa related to a-agglutinin core protein AGA1, hypothetical start	0.27
YALI0D15906g	similar to tr|Q9P8F7 Yarrowia lipolytica Triacylglycerol lipase precursor, start by similarity	0.27
YALI0C22924g	weakly similar to sp|P08640 Saccharomyces cerevisiae YIR019c STA1 extracellular alpha-1, 4-glucan glucosidase, hypothetical start	0.27
YALI0E15015g	no similarity, hypothetical start	0.27

YALI0F03366g	some similarities with tr|Q9VUB7 Drosophila melanogaster CG32133 protein, hypothetical start	0.27
YALI0D18381g	similar to tr|Q8TGI8 Talaromyces emersonii Beta-glucosidase, hypothetical start	0.27
YALI0A00110g	similar to CA0442|CaIFC4 Candida albicans, start by similarity	0.26
YALI0B20768g	similar to DEHA0A00979g Debaryomyces hansenii IPF 65.1, hypothetical start	0.26
YALI0D25784g	weakly similar to tr|O74782 Schizosaccharomyces pombe Hypothetical protein, hypothetical start	0.25
YALI0C15004g	no similarity, hypothetical start	0.25
YALI0A05885g	no similarity, hypothetical start	0.22
YALI0B16852g	similar to DEHA0B12474g Debaryomyces hansenii IPF 10115.1, hypothetical start	0.20
YALI0C06424g	similar to sp|P10870 Saccharomyces cerevisiae YDL194w SNF3 high-affinity glucose transporter/regulatory protein, start by similarity	0.17
YALI0D02995g	similar to tr|Q02804 Saccharomyces cerevisiae YPL051w ARL3 ADP-ribosylation factor-like protein, member of the arf-sar family in the ras superfamily, no start	0.16
YALI0E34265g	similar to sp|P06115 Saccharomyces cerevisiae YGR088w CTT1 catalase T, cytosolic P2.391.f2.1, hypothetical start	0.16
YALI0E05819g	weakly similar to sp|P08640 Saccharomyces cerevisiae YIR019c STA1 extracellular alpha-1, 4-glucan glucosidase, hypothetical start	0.14
YALI0D22957g	weakly similar to sp|Q12303 Saccharomyces cerevisiae YLR121c YPS3 GPI-anchored aspartyl protease 3 (yapsin 3), hypothetical start	0.13
YALI0A21461g	similar to sp|P36114 Saccharomyces cerevisiae YKR018c, hypothetical start	0.04
YALI0A21417g	similar to sp|O74267 Ashbya gossypii Threonine aldolase, hypothetical start	0.01
YALI0A21439g	no similarity	0.01
YALI0A21329g	weakly similar to CAGL0L02475g Candida glabrata, no start	0.01
YALI0A21307g	similar to sp|P25297 Saccharomyces cerevisiae YML123c PHO84 high-affinity inorganic phosphate/H+ symporter, hypothetical start	0.01

Table S7. Genes up-regulated (153) during the yeast to hyphae transition at 180 minues induced by GlNAc mediated by ZNC1.

Locus tag	Gene	Description	Fold change
YALI0E23859g		similar to sp|P38361 Saccharomyces cerevisiae YBR296c PHO89 Na+-coupled phosphate transport protein, high affinity, hypothetical start	101.72
YALI0E31603g		similar to KLLA0B14839g Kluyveromyces lactis IPF 6866.1, hypothetical start	79.26
YALI0C11165g		no similarity, hypothetical start	59.96
YALI0F25839g		similar to sp|P10281 Aspergillus oryzae ribonuclease T2 precursor and tr|Q02933 Saccharomyces cerevisiae YPL123c RNY1 ribonuclease T2, hypothetical start	46.32
YALI0B08426g		no similarity, hypothetical start	12.96
YALI0D03465g	YlPHO2	sp|P30887 Yarrowia lipolytica Acid phosphatase precursor PHO2 gene, identified start	9.56
YALI0C04026g		similar to DEHA0E25410g Debaryomyces hansenii IPF 10943.1, hypothetical start	8.84
YALI0B09867g		no similarity, hypothetical start	8.32
YALI0E35222g		similar to sp|P08540 Kluyveromyces lactis Potential acid phosphatase (EC 3.1.3.2), hypothetical start	7.86
YALI0C20251g		similar to sp|Q12068 Saccharomyces cerevisiae YOL151w GRE2, start by similarity	7.53
YALI0F13937g		highly similar to tr|O93968 Candida boidinii Formate dehydrogenase or tr|O13437 Candida boidinii NAD-dependent formate dehydrogenase, start by similarity	6.99
YALI0E19921g		weakly similar to wi|NCU05887.1 Neurospora crassa NCU05887.1, hypothetical start	6.64
YALI0E25003g		no similarity, possibly noncoding, hypothetical start	6.33
YALI0F28765g		highly similar to tr|O13437 Candida boidinii NAD-dependent formate dehydrogenase, start by similarity	6.29
YALI0C05731g		no similarity, hypothetical start	5.93
YALI0E27181g		similar to DEHA0A00979g Debaryomyces hansenii, hypothetical start	5.63
YALI0C23474g		no similarity, hypothetical start	5.34
YALI0F23551g		similar to tr|CAD60606 Podospora anserina, start by similarity	5.14
YALI0E19965g		no similarity, hypothetical start	4.97
YALI0A01023g		similar to sp|Q12691 Saccharomyces cerevisiae YDR038c ENA5 P-type ATPase involved in Na+ efflux, start by similarity	4.88
YALI0C16368g		similar to sp|P40565 Saccharomyces cerevisiae YIR005w IST3 similarity to RNA-binding proteins, hypothetical start	4.85
YALI0D25102g		no similarity, hypothetical start	4.84
YALI0B23408g		highly similar to sp|P40046 Saccharomyces cerevisiae YER072w VTC1 Negative Regulator of Cdc Fourty two (CDC42) singleton	4.78
YALI0C10252g		similar to sp|P43585 Saccharomyces cerevisiae YFL004w VTC2 putative polyphosphate synthetase, start by similarity	4.72
YALI0D15466g		no similarity	4.61
YALI0F29359g		weakly similar to tr|Q99296 Saccharomyces cerevisiae YLR149c unknown function,	4.53

		hypothetical start	
YALI0B19602g		similar to tr|Q9Y7W9 Yarrowia lipolytica Mycelial growth factor-1, start by similarity	4.43
YALI0C10945g		similar to CA0697|CaVTC4 Candida albicans CaVTC4 putative polyphosphate synthetase, start by similarity	4.35
YALI0C23991g		similar to tr|Q8J2S3 Hericium erinaceum Ribonuclease T2, hypothetical start	4.26
YALI0C11209g		no similarity, hypothetical start	4.15
YALI0E18546g		no similarity	3.98
YALI0E26686g		similar to tr|Q9HW37 Pseudomonas aeruginosa Hypothetical protein, start by similarity	3.75
YALI0C01859g		similar to sp|Q02253 Rattus norvegicus Methylmalonate-semialdehyde dehydrogenase (acylating), mitochondrial precursor (EC 1.2.1.27) (MMSDH), start by similarity	3.64
YALI0B03575g		weakly similar to sp|P21734 Saccharomyces cerevisiae UBC1_YEAST Ubiquitin-conjugating enzyme (Ubiquitin- protein ligase), hypothetical start	3.62
YALI0A08360g		weakly similar to sp|P09230 Yarrowia lipolytica Alkaline extracellular protease precursor (EC 3.4.21.-) (AEP), hypothetical start	3.53
YALI0C03564g		no similarity, hypothetical start	3.51
YALI0D22352g		highly similar to sp|P41797 Candida albicans Heat shock protein SSA1, start by similarity	3.50
YALI0D17622g		weakly similar to sp|P37610 Escherichia coli Alpha-ketoglutarate-dependent taurine dioxygenase, start by similarity	3.45
YALI0F08217g		weakly similar to sp|O14169 Schizosaccharomyces pombe gene SPAC4D7.02C and Saccharomyces cerevisiae YPL206c, hypothetical start	3.39
YALI0C05258g		weakly similar to ca|CA2975|CaARO9 Candida albicans aromatic amino acid aminotransferase II (by homology), start by similarity	3.38
YALI0F02607g		similar to tr|Q9C1L3 Neurospora crassa Putative 3-hydroxyisobutyrate dehydrogenase G6G8.5., hypothetical start	3.35
YALI0E21307g		similar to sp|P00175 Saccharomyces cerevisiae YML054c CYB2 lactate dehydrogenase cytochrome b2, hypothetical start	3.34
YALI0C07843g		similar to tr|Q872S8 Neurospora crassa B8B8.060 Hypothetical protein, hypothetical start	3.33
YALI0E06809g		some similarities with tr|Q12057 Saccharomyces cerevisiae YOR104w, hypothetical start	3.33
YALI0C06798g		highly similar to tr|Q9P5P3 Neurospora crassa Probable branching enzyme (be1), hypothetical start	3.32
YALI0D26488g		similar to tr|Q9C0V7 Schizosaccharomyces pombe Putative arylsulfatase, hypothetical start	3.32
YALI0E00110g		similar to sp|P08540|PHOX_KLULA Kluyveromyces lactis KLLA0B14839g Potential acid phosphatase, hypothetical start	3.24
YALI0E17787g	YlADH2	tr|Q9UW07 Yarrowia lipolytica Alcohol dehydrogenase 2, identified start	3.18
YALI0B07359g		similar to tr|Q870T8 Neurospora crassa B11H7.110 Probable nitrilase (NIT3) Neurospora crassa, start by similarity	3.15
YALI0C16951g		similar to tr|Q96X51 Pholiota nameko Probable metabolite transporter, start by similarity	3.15

YALI0E34507g	weakly similar to tr|Q9P8F7 Yarrowia lipolytica Triacylglycerol lipase precursor (EC 3.1.1.3), hypothetical start	3.13
YALI0F01628g	similar to DEHA-IPF7297.1 Debaryomyces hansenii, hypothetical start	3.09
YALI0A16588g	weakly similar to sp|P53552 Saccharomyces cerevisiae YNL139c RLR1 regulatory protein, hypothetical start	3.07
YALI0E08932g	no similarity, hypothetical start	3.04
YALI0F29909g	weakly similar to sp|Q03104 Saccharomyces cerevisiae YML128c GIN3 unknown function, start by similarity	2.99
YALI0C12518g	no similarity, hypothetical start	2.96
YALI0B21494g	some similarities with ca|CA1652|CaRNH1.exon1 Candida albicans Ribonuclease H, hypothetical start	2.92
YALI0E14190g	similar to sp|P17505 Saccharomyces cerevisiae YKL085w MDH1 malate dehydrogenase precursor, mitochondrial and tr|O94137 Piromyces sp. Malate dehydrogenase (EC 1.1.1. 37), no start	2.91
YALI0A15774g	similar to sp|P47159 Saccharomyces cerevisiae YJR124c, hypothetical start	2.87
YALI0D09339g	weakly similar to tr|O74251 Emericella nidulans Medusa transcriptional regulator, hypothetical start	2.85
YALI0F02651g	no similarity, hypothetical start	2.82
YALI0E19030g	no similarity	2.81
YALI0E00924g	weakly similar to sp|P38151 Saccharomyces cerevisiae YBR233w PBP2 PAB1 binding protein, hypothetical start	2.79
YALI0D11308g	similar to sp|P36059 Saccharomyces cerevisiae YKL151c similarity to C.elegans hypothetical protein R107. 2 and tr|O94347 Schizosaccharomyces pombe Conserved hypothetical protein, start by similarity	2.78
YALI0E14146g	some similarities with tr|Q96WV4 Schizosaccharomyces pombe SPBPJ4664.05 Unspecified membrane protein, hypothetical start	2.76
YALI0D24145g	weakly similar to tr|Q08991 Saccharomyces cerevisiae YPL279C, hypothetical start	2.76
YALI0C09240g	some similarities with sp|Q86JZ0|emb|AAO51355 Dictyostelium discoideum Hypothetical protein, hypothetical start	2.75
YALI0E20999g	weakly similar to tr|Q9P4F1 Aspergillus parasiticus Monooxigenase, hypothetical start	2.74
YALI0F22121g	similar to wi|NCU09058.1 Neurospora crassa NCU09058. 1 hypothetical protein, hypothetical start	2.74
YALI0E01562g	no similarity, hypothetical start	2.69
YALI0C20060g	similar to sp|P41928 Yarrowia lipolytica MTP2 Metallothionein-II, start by similarity	2.69
YALI0B18744g	no similarity, hypothetical start	2.67
YALI0D05005g	similar to sp|P34228 Saccharomyces cerevisiae YBL066c SEF1 putative transcription factor, start by similarity	2.67
YALI0E26279g	similar to tr|Q8DLR0 Synechococcus elongatus tll0418 Sulfolipid	2.66

		sulfoquinovosyldiacylglycerol biosynthesis protein, start by similarity	
YALI0D11660g		similar to tr|Q8RCT3 Thermoanaerobacter tengcongensis Uncharacterized enzyme involved in pigment biosynthesis, start by similarity	2.65
YALI0A16379g	YlADH3	tr|Q9UW06 Yarrowia lipolytica Alcohol dehydrogenase 3, identified start	2.65
YALI0E31581g		weakly similar to tr|Q871S2 Neurospora crassa 7F4. 170 Related to acid sphingomyelinase, hypothetical start	2.65
YALI0A18469g	YlHOY1	sp|Q99160 Yarrowia lipolytica Homeobox protein HOY1, identified start	2.62
YALI0B21846g		similar to sp|Q00415 Coccidioides immitis 4-hydroxyphenylpyruvate dioxygenase (EC 1.13.11.27) (4HPPD) (HPD) (HPPDase), start by similarity	2.61
YALI0C00847g		similar to DEHA0A10670g Debaryomyces hansenii IPF 7135.1, hypothetical start	2.59
YALI0F01650g		weakly similar to tr|Q9WYD3 Thermotoga maritima Oxidoreductase, short chain dehydrogenase/reductase family, hypothetical start	2.58
YALI0F18414g		similar to tr|Q03920 Saccharomyces cerevisiae YDR140w, start by similarity	2.57
YALI0D09889g		no similarity	2.57
YALI0F18084g		similar to tr|Q9P3B9 Neurospora crassa Related to myo-inositol transport protein ITR1, start by similarity	2.56
YALI0B23100g		weakly similar to DEHA0E01430g Debaryomyces hansenii IPF 5716.1, hypothetical start	2.56
YALI0D10725g		no similarity, hypothetical start	2.55
YALI0E18502g		similar to DEHA0F24992g Debaryomyces hansenii, hypothetical start	2.55
YALI0E12859g		similar to sp|P38137 Saccharomyces cerevisiae YBR222c PCS60 AMP-binding protein, peroxisomal-coenzyme A synthetase, hypothetical start	2.53
YALI0C23452g		no similarity, hypothetical start	2.53
YALI0B04004g			2.53
YALI0C19338g		no similarity, possibly noncoding, hypothetical start	2.50
YALI0F16401g		similar to wi|NCU08356.1 Neurospora crassa NCU08356. 1 hypothetical protein, start by similarity	2.50
YALI0A12419g		no similarity	2.48
YALI0E05313g		similar to tr|Q08777 Saccharomyces cerevisiae YOR306c, hypothetical start	2.47
YALI0D21076g		similar to CA2439|IPF7514 Candida albicans unknown function, hypothetical start	2.45
YALI0D24057g		weakly similar to sp|P53195 Saccharomyces cerevisiae YGL005c weak similarity to Xenopus kinesin-related protein Eg5, hypothetical start	2.42
YALI0F05038g		similar to tr|Q9P5K8 Neurospora crassa Probable 3-methyl-2-oxobutanoate dehydrogenase (Lipoamide)E1 beta chain, hypothetical start	2.41
YALI0D07942g		similar to sp|P47771 Saccharomyces cerevisiae YMR170c ALD5 aldehyde dehydrogenase 2 (NAD+), start by similarity	2.41
YALI0C01485g		similar to sp|Q04174 Saccharomyces cerevisiae SMP3 protein kinase C pathway protein, start by similarity	2.41
YALI0F26191g		similar to sp|P38067 Saccharomyces cerevisiae YBR006w UGA2 succinate	2.41

	semialdehyde dehydrogenase, hypothetical start	
YALI0C19668g	similar to sp|Q12335 Saccharomyces cerevisiae YDR032c PST2, start by similarity	2.40
YALI0F04378g	similar to sp|Q04782 Candida albicans Lanosterol synthase (EC 5.4.99.7) (Oxidosqualene--lanosterol cyclase) (2, 3-epoxysqualene--lanosterol cyclase) (OSC), hypothetical start	2.40
YALI0E27093g	no similarity, hypothetical start	2.40
YALI0F26587g	similar to CA0994|IPF10077 Candida albicans, hypothetical start	2.39
YALI0E03586g	weakly similar to wi|NCU01320.1 Neurospora crassa NCU01320.1, hypothetical start	2.38
YALI0E27511g	similar to sp|Q03558 Saccharomyces cerevisiae YHR179w OYE2 NADPH dehydrogenase (old yellow enzyme), isoform 1, hypothetical start	2.38
YALI0E23672g	weakly similar to CA2830|CaRBT1 Candida albicans CaRBT1 repressed by TUP1 protein 1, hypothetical start	2.36
YALI0F22847g	no similarity, hypothetical start	2.36
YALI0E17677g	similar to tr|Q03419 Saccharomyces cerevisiae YDR492w or tr|Q12442 Saccharomyces cerevisiae YOL002C, hypothetical start	2.35
YALI0C01397g	weakly similar to DEHA0B05676g Debaryomyces hansenii IPF 9611.1, hypothetical start	2.34
YALI0E18634g	similar to sp|P36013 Saccharomyces cerevisiae YKL029c MAE1 malic enzyme, hypothetical start	2.33
YALI0E00638g	similar to tr|Q9TEM3 Emericella nidulans MCSA Methylcitrate synthase precursor, hypothetical start	2.33
YALI0C02981g	weakly similar to sp|P47000 Saccharomyces cerevisiae YJL159w HSP150 member of the PIR1P/HSP150P/PIR3P family P5.2.f4.1, hypothetical start	2.32
YALI0C06534g	no similarity	2.32
YALI0B16522g	similar to sp|P19145 Saccharomyces cerevisiae YKR039w GAP1 general amino acid permease, hypothetical start	2.32
YALI0D07260g	no similarity, hypothetical start	2.31
YALI0A00759g	similar to sp|Q01317 Neurospora crassa Ankyrin repeat protein nuc-2, start by similarity	2.30
YALI0C23276g	no similarity, hypothetical start	2.30
YALI0F11077g	similar to sp|P38841 Saccharomyces cerevisiae YHR138c, hypothetical start	2.29
YALI0D08690g	similar to sp|P50136 Mus musculus 2-oxoisovalerate dehydrogenase alpha subunit, mitochondrial precursor, start by similarity	2.29
YALI0E03212g	similar to sp|P32891 Saccharomyces cerevisiae YDL174c DLD1 D-lactate ferricytochrome C oxidoreductase (D-LCR), possible transmembrane segment, hypothetical start	2.29
YALI0A16841g	weakly similar to sp|P41696 Saccharomyces cerevisiae YOR113w AZF1 asparagine-rich zinc finger protein, hypothetical start	2.28
YALI0F22209g	some similarities with sp|P40011 Saccharomyces cerevisiae YER010c, hypothetical start	2.27
YALI0C16049g	some similarities with sp|P26642 Xenopus laevis Elongation factor 1-gamma type 1 (EF-	2.27

	1-gamma) (P47), start by similarity	
YALI0C21450g	similar to sp|P47085 Saccharomyces cerevisiae YJR008w, start by similarity	2.27
YALI0C04433g	highly similar to sp|Q92413 Emericella nidulans Ornithine aminotransferase (EC 2.6.1.13) (Ornithine--oxo-acid aminotransferase), start by similarity	2.26
YALI0E30327g	weakly similar to tr|Q9C0W5 Schizosaccharomyces pombe SPBC800.14C Hypothetical 17.7 kDa protein, hypothetical start	2.26
YALI0B02544g	similar to tr|Q96VK5 Emericella nidulans ABC transporter protein, hypothetical start	2.26
YALI0C05753g	highly similar to DEHA0B09460g Debaryomyces hansenii IPF 9880.1, hypothetical start	2.25
YALI0F04169g	similar to sp|P06738 Saccharomyces cerevisiae YPR160w GPH1 glycogen phosphorylase, hypothetical start	2.25
YALI0C08811g	similar to tr|Q9K6C9 Bacillus halodurans 3-hydroxybutyryl-CoA dehydrogenase, hypothetical start	2.24
YALI0E04422g	no similarity, hypothetical start	2.24
YALI0E02266g	weakly similar to tr|Q9P3Q3 Neurospora crassa B24P7. 320 Related to cytosolic Cu/Zn superoxide dismutase, hypothetical start	2.22
YALI0E18238g	similar to sp|P17649 Saccharomyces cerevisiae YGR019w UGA1 4-aminobutyrate aminotransferase (GABA transaminase), hypothetical start	2.22
YALI0F19140g	no similarity, hypothetical start	2.22
YALI0D23815g	similar to tr|Q9VXY3 Drosophila melanogaster CG5599 protein (LD43554P), hypothetical start	2.21
YALI0B14685g	no similarity, hypothetical start	2.21
YALI0C10626g	no similarity, hypothetical start	2.20
YALI0D05041g	some similarities with DEHA0A12749g Debaryomyces hansenii IPF 7296.1, probable Zn finger transcription factor, start by similarity	2.19
YALI0A02629g	no similarity	2.18
YALI0F23749g	highly similar to wi|NCU02291.1 Neurospora crassa NCU02291.1 hypothetical protein, probable Glutaryl-CoA dehydrogenase, hypothetical start	2.18
YALI0C16566g	highly similar to sp|P04050 Saccharomyces cerevisiae YDL140c RPO21 DNA-directed RNA polymerase II, 215 KD subunit	2.17
YALI0C15466g	no similarity, hypothetical start	2.15
YALI0D10813g	similar to tr|Q9P866 Candida albicans Phosphatidylinositol phospholipase C, hypothetical start	2.10
YALI0C21065g	some similarities with sp|P47011 Saccharomyces cerevisiae YJL137c GLG2 self-glucosylating initiator of glycogen synthesis P2.283.f2.1, hypothetical start	2.09
YALI0C19800g	weakly similar to tr|Q91255 Petromyzon marinus NF-180, hypothetical start	2.09
YALI0F30041g	similar to tr|Q06593 Saccharomyces cerevisiae YPR194c OPT2 oligopeptide transporter, hypothetical start	2.07
YALI0C00209g	similar to sp|P32784 Saccharomyces cerevisiae YBL011w SCT1 suppresses a choline-	2.07

	transport mutant, hypothetical start	
YALI0D07986g	weakly similar to tr|Q9FUL6 Perilla frutescens Diacylglycerol acyltransferase, hypothetical start	2.07
YALI0C24145g	some similarities with tr|Q9HGM4 Schizosaccharomyces pombe Hypothetical 28.8 kDa protein, hypothetical start	2.06
YALI0D08250g	similar to wi|NCU01449.1 Neurospora crassa NCU01449. 1 hypothetical protein, hypothetical start	2.06
YALI0E11407g	weakly similar to tr|Q8J2U0 Aspergillus oryzae MOXY monooxygenase, hypothetical start	2.05
YALI0F04059g	similar to DEHA0G23474g Debaryomyces hansenii IPF 4877.1, start by similarity	2.03

Table S8. Genes down-regulated (102) during the yeast to hyphae transition at 180 minues induced by GlNAc mediated by ZNC1.

Locus tag	Gene	Description	Fold change
YALI0A19140g		similar to tr|Q07897 Saccharomyces cerevisiae YLR003c, hypothetical start	0.50
YALI0C00539g		similar to DEHA0G13255g Debaryomyces hansenii IPF 3883.1, start by similarity	0.48
YALI0D02519g		weakly similar to sp|Q04223 Saccharomyces cerevisiae YMR130w, hypothetical start	0.48
YALI0E09196g		similar to DEHA0C15686g Debaryomyces hansenii, start by similarity	0.48
YALI0B05368g		highly similar to sp|P28274 Saccharomyces cerevisiae CTP synthase 1 (EC 6.3.4.2) (UTP--ammonia ligase 1), hypothetical start	0.48
YALI0F21725g		similar to sp|P40010 Saccharomyces cerevisiae Hypothetical GTP-binding protein (YER006w), start by similarity	0.47
YALI0E25047g		no similarity, hypothetical start	0.47
YALI0B21516g		similar to sp|P53759 Saccharomyces cerevisiae YML080w similarity to A.brasilense nifR3 protein P3.77.f3. 1, start by similarity	0.47
YALI0F24739g		highly similar to sp|P05736 Saccharomyces cerevisiae YIL018w RPL5A 60S large subunit ribosomal protein L8.e [INTRON], start by similarity	0.47
YALI0F31317g		similar to sp|P33201 Saccharomyces cerevisiae YKL009w MRT4 mRNA turnover 4, start by similarity	0.47
YALI0B03278g		some similarities with tr|Q8R2M2 Mus musculus Similar to acidic 82 kDa protein mRNA, hypothetical start	0.46
YALI0C08877g		similar to tr|O74965 Schizosaccharomyces pombe Hypothetical 63.5 kDa protein, start by similarity	0.46
YALI0D24299g		similar to sp|P78954 Schizosaccharomyces pombe Eukaryotic translation initiation factor 4E-1 (eIF-4E-1) (eIF4E-1) (mRNA cap-binding protein 1) (eIF-4F 25 kDa subunit 1), hypothetical start	0.45
YALI0E19338g		similar to tr|Q96X39 Debaryomyces hansenii Plasma membrane Na+ ATPase, hypothetical start	0.45
YALI0E11517g		weakly similar to tr|Q8TFK5 Yarrowia lipolytica CWP1 Cell wall protein, start by similarity	0.45
YALI0A03817g		weakly similar to CA5921|IPF8930 Candida albicans unknown function, hypothetical start	0.45
YALI0B11462g		no similarity, hypothetical start	0.45
YALI0C11946g		weakly similar to sp|P19658 Saccharomyces cerevisiae YJL085w EXO70 70 kDa exocyst complex protein, hypothetical start	0.45
YALI0C03267g		no similarity, hypothetical start	0.44
YALI0E18854g		some similarities with sp|P87058 Schizosaccharomyces pombe La protein homolog (La ribonucleoprotein) (La autoantigen homolog), hypothetical start	0.43
YALI0A10310g		similar to sp|P51601 Saccharomyces cerevisiae YGR267c FOL2 GTP cyclohydrolase I, start by similarity	0.43
YALI0C01738g		similar to sp|P35184 Saccharomyces cerevisiae Ribosome assembly protein SQT1, start	0.43

	by similarity	
YALI0E31779g	similar to CA0241|IPF15630 Candida albicans IPF15630, hypothetical start	0.43
YALI0F20658g	similar to sp|Q06706 Saccharomyces cerevisiae YLR384c IKI3 confers sensitivity to killer toxin, hypothetical start	0.43
YALI0D14630g	similar to sp|P51996 Saccharomyces cerevisiae YGL210w YPT32 small GTP-binding protein essential for Golgi function, start by similarity	0.42
YALI0B12738g	no similarity, hypothetical start	0.42
YALI0D10989g	weakly similar to tr|Q07527 Saccharomyces cerevisiae YDL112w TRM3 tRNA (guanosine-2 -O-)-methyltransferase, hypothetical start	0.42
YALI0A00286g	highly similar to sp|P39729 Saccharomyces cerevisiae YAL036c FUN11 probable GTP-binding protein, start by similarity	0.42
YALI0F08019g	weakly similar to tr|Q12015 Saccharomyces cerevisiae YOR223w, hypothetical start	0.42
YALI0D14410g	weakly similar to DEHA0C15796g Debaryomyces hansenii IPF 3508.1, start by similarity	0.42
YALI0B02222g	similar to sp|Q12184 Saccharomyces cerevisiae YPL252c Adrenodoxin homolog, mitochondrial precursor (Mitochondrial ferredoxin), hypothetical start	0.41
YALI0D21010g	similar to KLLA0B05709g Kluyveromyces lactis IPF 7503.1, hypothetical start	0.41
YALI0F06182g	similar to sp|P46669 Saccharomyces cerevisiae YOR340c RPA43 DNA-directed RNA polymerase I, 36 KD subunit, hypothetical start	0.41
YALI0B18040g	similar to sp|P36095 Saccharomyces cerevisiae YKL041w VPS24 endosomal Vps protein complex subunit singleton, start by similarity	0.40
YALI0B23342g	highly similar to sp|P53261 Saccharomyces cerevisiae YGR103w similarity to zebrafish essential for embryonic development gene pescadillo singleton, start by similarity	0.40
YALI0B09119g	similar to sp|P25040 Saccharomyces cerevisiae YOL022c, hypothetical start	0.40
YALI0E06039g	similar to sp|P48240 Saccharomyces cerevisiae YGR158c MTR3 involved in mRNA transport and DEHA0A13343g Debaryomyces hansenii, start by similarity	0.40
YALI0A07106g	weakly similar to DEHA0F09801g Debaryomyces hansenii IPF 8358.1, start by similarity	0.39
YALI0D20416g	similar to tr|O74534 Schizosaccharomyces pombe Stxbp-unc-18-sec1 family protien transport protein	0.39
YALI0D17116g	highly similar to sp|Q12460 Saccharomyces cerevisiae YLR197w SIK1 involved in pre-rRNA processing, start by similarity	0.39
YALI0C16500g	no similarity, hypothetical start	0.38
YALI0C21648g	similar to sp|P14284 Saccharomyces cerevisiae DNA polymerase zeta catalytic subunit (EC 2.7.7.7), start by similarity	0.38
YALI0B08514g	similar to sp|Q05636 Saccharomyces cerevisiae Exosome complex exonuclease RRP45 (EC 3.1.13.-) (Ribosomal RNA processing protein 45), hypothetical start	0.37
YALI0E26389g	similar to sp|Q04660 Saccharomyces cerevisiae YMR049c, hypothetical start	0.37
YALI0F04708g	similar to sp|P36080 Saccharomyces cerevisiae YKL082c, start by similarity	0.36
YALI0F12529g	similar to wi|NCU03084.1 Neurospora crassa NCU03084. 1 hypothetical protein, start by	0.36

	similarity	
YALI0C21868g	highly similar to tr|Q03940 Saccharomyces cerevisiae YDR190c RVB1 RUVB-like protein P2.38.f2.1, start by similarity	0.36
YALI0B12210g	similar to sp|P41391 Schizosaccharomyces pombe Ran GTPase activating protein 1 (Protein rna1), start by similarity	0.36
YALI0E20669g	similar to KLLA0F03058g Kluyveromyces lactis, hypothetical start	0.36
YALI0D14102g	similar to sp|P40018 Saccharomyces cerevisiae YER029c SMB1 associated with U1 snRNP as part of the Sm-core that is common to all spliceosomal snRNPs, start by similarity	0.35
YALI0E12397g	similar to sp|P47173 Saccharomyces cerevisiae YJR142w, hypothetical start	0.35
YALI0E05819g	weakly similar to sp|P08640 Saccharomyces cerevisiae YIR019c STA1 extracellular alpha-1, 4-glucan glucosidase, hypothetical start	0.35
YALI0E10351g	weakly similar to sp|Q9Y7N2 Schizosaccharomyces pombe RNA polymerase II mediator complex protein rox3, hypothetical start	0.35
YALI0D23859g	highly similar to sp|P53941 Saccharomyces cerevisiae U3 small nucleolar ribonucleoprotein protein IMP4, start by similarity	0.34
YALI0B10560g	similar to sp|P40991 Saccharomyces cerevisiae YNL061w NOP2 nucleolar protein, start by similarity	0.34
YALI0F15235g	similar to DEHA0C12419g Debaryomyces hansenii, start by similarity	0.33
YALI0D11792g	similar to CA0750|IPF6284 Candida albicans IPF6284 unknown function, hypothetical start	0.33
YALI0F30877g	similar to sp|P40202 Saccharomyces cerevisiae YMR038c LYS7 copper chaperone for superoxide dismutase, start by similarity	0.33
YALI0F31339g	weakly similar to wi|NCU06553.1 Neurospora crassa NCU06553.1 hypothetical protein (AF309689) protein G6G8.9, hypothetical start	0.33
YALI0B09581g	no similarity, hypothetical start	0.32
YALI0C13596g	weakly similar to DEHA0G04499g Debaryomyces hansenii IPF 725.1, hypothetical start	0.32
YALI0E17303g	weakly similar to tr|CAD60707 Podospora anserina CAD60707, hypothetical start	0.31
YALI0E09306g	weakly similar to KLLA0E08899g Kluyveromyces lactis, hypothetical start	0.31
YALI0E26939g	similar to sp|P54964 Saccharomyces cerevisiae YLR059c YNT20, hypothetical start	0.31
YALI0F04686g	no similarity, hypothetical start	0.30
YALI0D21648g	some similarities with DEHA0G17006g Debaryomyces hansenii IPF 273.1, hypothetical start	0.30
YALI0E19052g	weakly similar to DEHA0D10505g Debaryomyces hansenii, hypothetical start	0.30
YALI0B01782g	similar to tr|Q96VT2 Aspergillus niger prolyl aminopeptidase A (EC 3.4.11.5), start by similarity	0.30
YALI0F12771g	some similarities with tr|Q12400 Saccharomyces cerevisiae Chromosome XV reading frame ORF YOL093W, hypothetical start	0.29

YALI0F08635g	weakly similar to sp|P32349 Saccharomyces cerevisiae YPR190c RPC82 DNA-directed RNA polymerase III, 82 KD subunit, start by similarity	0.29
YALI0F08657g	weakly similar to tr|Q06679 Saccharomyces cerevisiae YDR324c, start by similarity	0.28
YALI0F23969g	similar to wi|NCU02176.1 Neurospora crassa NCU02176. 1, conserved hypothetical protein, hypothetical start	0.27
YALI0A03509g	similar to sp|P37838 Saccharomyces cerevisiae YPL043w NOP4 nucleolar protein P15, start by similarity	0.26
YALI0B05764g	weakly similar to sp|P38758 Saccharomyces cerevisiae Hypothetical 57.0 kDa protein in SOD2-RPL27A intergenic region, start by similarity	0.26
YALI0B18414g	weakly similar to tr|Q8BH76 Mus musculus DNA polymerase delta subunit 3, start by similarity	0.26
YALI0B03784g	similar to tr|Q9HEC4 Neurospora crassa Conserved hypothetical protein, hypothetical start	0.26
YALI0D05511g	similar to sp|P38792 Saccharomyces cerevisiae YHR069c RRP4 3 -5 exoribonuclease required for 3 end formation of 5.8S rRNA (Exosome complex), start by similarity	0.26
YALI0E16280g	weakly similar to tr|Q04177 Saccharomyces cerevisiae YDR398W snoRNA binding activity, start by similarity	0.26
YALI0F26609g	similar to sp|P28789 Saccharomyces cerevisiae YDL205c HEM3 porphobilinogen deaminase, start by similarity	0.25
YALI0B01980g	some similarities with sp|P87250 Kluyveromyces lactis mitochondrial replication protein MTF1, hypothetical start	0.23
YALI0C14388g	similar to tr|O74529 Schizosaccharomyces pombe Probable methyltransferase, start by similarity	0.23
YALI0D07788g	similar to sp|P17890 Saccharomyces cerevisiae YNL151c RPC31 DNA-directed RNA polymerase III, 31 KD subunit, hypothetical start	0.23
YALI0D26884g	weakly similar to tr|O15387 Homo sapiens MOV34 ISOLOG, hypothetical start	0.23
YALI0F16731g	weakly similar to tr|Q06522 Saccharomyces cerevisiae YPR147c, hypothetical start	0.23
YALI0F29205g	similar to sp|P53720 Saccharomyces cerevisiae YNR015w SMM1 tRNA dihydrouridine synthase, start by similarity	0.22
YALI0D17028g	similar to sp|Q12001 Saccharomyces cerevisiae YOR002w ALG6 glucosyltransferase, hypothetical start	0.22
YALI0F10747g	similar to sp|P25631 Saccharomyces cerevisiae YCR051w and DEHA0F25608g Debaryomyces hansenii and KLLA0C09108g Kluyveromyces lactis, start by similarity	0.22
YALI0F28083g	similar to sp|P53742 Saccharomyces cerevisiae YNR053c NOG2 GTPase, start by similarity	0.20
YALI0F13475g	similar to DEHA0A13563g Debaryomyces hansenii IPF 7368.1, hypothetical start	0.19
YALI0A21263g	similar to sp|O93852 Candida albicans D-arabinono-1, 4-lactone oxidase, hypothetical start	0.18

YALI0F09856g	similar to sp|P40055 Saccharomyces cerevisiae YER082c and DEHA0B10747g Debaryomyces hansenii, hypothetical start	0.17
YALI0A20614g	similar to tr|Q06506 Saccharomyces cerevisiae of the beta-transducin family of guanine nucleotide-binding, hypothetical start	0.16
YALI0C09526g	weakly similar to sp|P25567 Saccharomyces cerevisiae YCL037c SRO9 La motif-containing proteins that modulate mRNA translation, hypothetical start	0.14
YALI0B16852g	similar to DEHA0B12474g Debaryomyces hansenii IPF 10115.1, hypothetical start	0.14
YALI0E19481g	similar to tr|Q9C245 Neurospora crassa ubiquitin related modifier URM1, hypothetical start	0.12
YALI0C15004g	no similarity, hypothetical start	0.11
YALI0E17545g	weakly similar to sp|P09950 Saccharomyces cerevisiae YDR232w HEM1 5-aminolevulinate synthase, hypothetical start	0.11
YALI0A21307g	similar to sp|P25297 Saccharomyces cerevisiae YML123c PHO84 high-affinity inorganic phosphate/H+ symporter, hypothetical start	0.02
YALI0A21329g	weakly similar to CAGL0L02475g Candida glabrata, no start	0.02
YALI0A21461g	similar to sp|P36114 Saccharomyces cerevisiae YKR018c, hypothetical start	0.01
YALI0A21417g	similar to sp|O74267 Ashbya gossypii Threonine aldolase, hypothetical start	0.01
YALI0A21439g	no similarity	0.01

Table S9a. Up-regulated genes (3) at 15, 60 and 180 minutes of induction.

			Fold change		
**Locus tag**	**Gene**	**Description**	**15 min**	**60 min**	**180 min**
YALI0E23859g		similar to sp|P38361 Saccharomyces cerevisiae YBR296c PHO89 Na+-coupled phosphate transport protein, high affinity, hypothetical start	5.09	9.27	101.70
YALI0C11165g		no similarity, hypothetical start	8.97	22.64	59.96
YALI0B08426g		no similarity, hypothetical start	2.43	5.17	12.96

Table S9 b. Up-regulated genes (22) at 15 and 60 minutes of induction.

			Fold change		
**Locus tag**	**Gene**	**Description**	**15 min**	**60 min**	**180 min**
YALI0F30393g		similar to sp|P25586 Saccharomyces cerevisiae YCL059c KRR1 unknown function, start by similarity	4.93	2.25	-
YALI0F23485g		some similarities with wi|NCU02191.1 Neurospora crassa NCU02191.1 hypothetical protein, hypothetical start	1.75	4.14	-
YALI0F00462g		similar to sp|O43315 Homo sapiens Aquaporin 9	3.29	3.23	-
YALI0E14707g		no similarity, hypothetical start	2.11	3.43	-
YALI0E05533g	YILYC1	sp|P41929 Lysine acetyltransferase, identified start	2.65	2.70	-
YALI0E01584g		similar to DEHA0E11000g Debaryomyces hansenii, hypothetical start	2.06	6.49	-
YALI0E01210g		no similarity, hypothetical start	5.41	3.00	-
YALI0E01100g		similar to sp|P21304 Saccharomyces cerevisiae YLR196w PWP1, start by similarity	9.86	4.05	-
YALI0E00550g		similar to sp|P36105 Saccharomyces cerevisiae YKL006w RPL14A ribosomal protein or sp|P38754 Saccharomyces cerevisiae YHL001w RPL14B ribosomal protein, hypothetical start	2.26	2.90	-
YALI0D24343g		weakly similar to sp|P47045 Saccharomyces cerevisiae Hypothetical 54.2 kDa protein in BTN1-PEP8 intergenic region, hypothetical start	2.82	2.78	-
YALI0D17116g		highly similar to sp|Q12460 Saccharomyces cerevisiae YLR197w SIK1 involved in pre-rRNA processing, start by similarity	2.36	2.99	-

YALI0D11792g		similar to CA0750|IPF6284 Candida albicans IPF6284 unknown function, hypothetical start	3.19	2.43	-
YALI0D05159g		similar to sp|P38174 Saccharomyces cerevisiae YBL091c MAP2 methionine aminopeptidase, isoform 2, start by similarity	3.19	2.74	-
YALI0C21362g		similar to sp|P39676 Saccharomyces cerevisiae YGR234w YHB1 flavohemoglobin, start by similarity	2.07	2.21	-
YALI0C19624g		similar to sp|P38071 Saccharomyces cerevisiae YBR026c	2.24	3.12	-
YALI0C11341g		highly similar to tr|Q12230 Saccharomyces cerevisiae Hypothetical 38.1 kDa protein YPL004C, hypothetical start	2.50	3.11	-
YALI0C02717g		similar to KLLA0E08767g Kluyveromyces lactis, start by similarity	2.41	2.75	-
YALI0B09779g		highly similar to DEHA0G23012g Debaryomyces hansenii IPF 4913.1, start by similarity	3.20	7.10	-
YALI0B08052g		similar to DEHA0A06347g Debaryomyces hansenii, start by similarity	6.11	3.97	-
YALI0B07051g		no similarity, hypothetical start	20.34	3.58	-
YALI0B02222g		similar to sp|Q12184 Saccharomyces cerevisiae YPL252c Adrenodoxin homolog, mitochondrial precursor	2.46	2.46	-
YALI0A20614g		similar to tr|Q06506 Saccharomyces cerevisiae of the beta-transducin family of guanine nucleotide-binding, hypothetical start	2.86	2.77	-

Table S9 c. Up-regulated genes (2) at 15 and 180 minutes of induction.

			Fold change		
**Locus tag**	**Gene**	**Description**	**15 min**	**60 min**	**180 min**
YALI0F13937g		highly similar to tr|O93968 Candida boidinii Formate dehydrogenase or tr|O13437 Candida boidinii NAD-dependent formate dehydrogenase, start by similarity	10.60	-	6.99
YALI0C20251g		similar to sp|Q12068 Saccharomyces cerevisiae YOL151w GRE2, start by similarity	2.64	-	7.53

Table S9 d. Up-regulated genes (14) at 60 and 180 minutes of induction.

Locus tag	Gene	Description	Fold change		
			15 min	60 min	180 min
YALI0C05731g		no similarity, hypothetical start	-	2.56	5.93
YALI0C06798g		highly similar to tr|Q9P5P3 Neurospora crassa Probable branching enzyme	-	2.38	3.32
YALI0C07843g		similar to tr|Q872S8 Neurospora crassa B8B8.060 Hypothetical protein, hypothetical start	-	3.22	3.33
YALI0C11209g		no similarity, hypothetical start	-	7.52	4.15
YALI0C21065g		some similarities with sp|P47011 Saccharomyces cerevisiae YJL137c GLG2 self-glucosylating initiator of glycogen synthesis P2.283.f2.1, hypothetical start	-	2.17	2.09
YALI0C23452g		no similarity, hypothetical start	-	2.70	2.53
YALI0D05005g		similar to sp|P34228 Saccharomyces cerevisiae YBL066c SEF1 putative transcription factor, start by similarity	-	2.41	2.67
YALI0D08690g		similar to sp|P50136 Mus musculus 2-oxoisovalerate dehydrogenase alpha subunit, mitochondrial precursor, start by similarity	-	4.49	2.29
YALI0D26488g		similar to tr|Q9C0V7 Schizosaccharomyces pombe Putative arylsulfatase, hypothetical start	-	2.32	3.32
YALI0E05313g		similar to tr|Q08777 Saccharomyces cerevisiae YOR306c, hypothetical start	-	4.22	2.47
YALI0E19965g		no similarity, hypothetical start	-	2.99	4.97
YALI0E25003g		no similarity, possibly noncoding, hypothetical start	-	2.39	6.33
YALI0E26686g		similar to tr|Q9HW37 Pseudomonas aeruginosa Hypothetical protein, start by similarity	-	2.63	3.75
YALI0E31603g		similar to KLLA0B14839g Kluyveromyces lactis IPF 6866.1, hypothetical start	-	3.09	79.26

Table S10a. Down-regulated genes (9) at 15, 60 and 180 minutes of induction.

Locus tag	Gene	Description	Fold change 15 min	60 min	180 min
YALI0A21263g		similar to sp|O93852 Candida albicans D-arabinono-1, 4-lactone oxidase, hypothetical start	0.21	0.35	0.18
YALI0A21307g		similar to sp|P25297 Saccharomyces cerevisiae YML123c PHO84 high-affinity inorganic phosphate/H+ symporter, hypothetical start	0.05	0.01	0.02
YALI0A21329g		weakly similar to CAGL0L02475g Candida glabrata, no start	0.01	0.01	0.01
YALI0A21417g		similar to sp|O74267 Ashbya gossypii Threonine aldolase, hypothetical start	0.03	0.01	0.01
YALI0A21439g		no similarity	0.03	0.01	0.01
YALI0A21461g		similar to sp|P36114 Saccharomyces cerevisiae YKR018c, hypothetical start	0.04	0.04	0.01
YALI0B16852g		similar to DEHA0B12474g Debaryomyces hansenii IPF 10115.1, hypothetical start	0.23	0.20	0.14
YALI0E05819g		weakly similar to sp|P08640 Saccharomyces cerevisiae YIR019c STA1 extracellular alpha-1, 4-glucan glucosidase, hypothetical start	0.22	0.14	0.35
YALI0E11517g		weakly similar to tr|Q8TFK5 Yarrowia lipolytica CWP1 Cell wall protein, start by similarity	0.48	0.33	0.45

Table S10b. Down-regulated genes (10) at 15 and 60 minutes of induction.

Locus tag	Gene	Description	Fold change 15 min	60 min	180 min
YALI0A19910g		similar to sp|P47137 Saccharomyces cerevisiae Probable oxidoreductase YJR096W, hypothetical start	0.45	0.36	-
YALI0B20636g		weakly similar to tr|Q89QU1 Bradyrhizobium japonicum Bll3033 protein, hypothetical start	0.39	0.40	-
YALI0B20768g		similar to DEHA0A00979g Debaryomyces hansenii IPF 65.1, hypothetical start	0.41	0.26	-
YALI0B21318g		no similarity, hypothetical start	0.45	0.40	-
YALI0C20405g		similar to sp|P10768 Homo sapiens Esterase D	0.49	0.37	-

YALI0D19866g		similar to CAGL0I08613g Candida glabrata, hypothetical start	0.33	0.45	-
YALI0D22957g		weakly similar to sp|Q12303 Saccharomyces cerevisiae YLR121c YPS3 GPI-anchored aspartyl protease 3	0.33	0.13	-
YALI0E34265g		similar to sp|P06115 Saccharomyces cerevisiae YGR088w CTT1 catalase T, cytosolic P2.391.f2.1, hypothetical start	0.38	0.16	-
YALI0F22319g		no similarity, hypothetical start	0.32	0.35	-
YALI0F25685g		weakly similar to sp|P38355 Saccharomyces cerevisiae YBR287w unknown function, start by similarity	0.37	0.46	-

Table S10c. Down-regulated genes (0) at 15 and 180 minutes of induction.

			Fold change		
Locus tag	Gene	Description	15 min	60 min	180 min
-	-	-	-	-	-

Table S10d. Down-regulated genes (1) at 60 and 180 minutes of induction.

			Fold change		
Locus tag	Gene	Description	15 min	60 min	180 min
YALI0C15004g		no similarity, hypothetical start	-	0.25	0.11
