# Supplementary material for: Identification of the Transcription Factor Znc1p, which Regulates the Yeast-to-Hypha Transition in the Dimorphic Yeast Yarrowia lipolytica
Source: PLoS One. 2013 Jun 24;8(6):e66790. doi: 10.1371/journal.pone.0066790 (PMC3691278; doi:10.1371/journal.pone.0066790)
Supplement: Files S2 — Contains Table S11. (PDF) [file pone.0066790.s005.pdf]

TABLE S11. Plasmid used in this study

Plasmid	Genotype	Source
pINA443	Replicative form with *ARS68*, *URA3*	C. Gallardin[a]
pINA445	Replicative form with *ARS68*, *LEU2*	C. Gallardin[a]
pJCTGCAN18	Plasmid recovered from the genomic DNA library, complementing the fil$^-$ phenotype in CHY33188 strain. Replicative form with *ARS68*, *LEU2*.	This study
pJCTGCAN203	Plasmid constructed from pJCTGCAN18, *ZNC1* tagged with *GFP* in the *Spe*I site.	This study
pJCTGCAN227	The 2.2-kbp *Sal*I-*Xba*I fragment from pJCTGCAN18 containing the *ZNC1* gene cloned into pBlueScript II SK(+).	This study
pJCTGCAN277	From pJCTGCAN227. Two cysteine residues within the Znc1p zinc finger region were replaced by two arginines ($C_{22}C_{25}/R_{22}R_{25}$).	This study
pJCTGCAN282	From pJCTGCAN227. Two leucine residues within the Znc1p leucine zipper region were replaced by two arginines ($L_{437}L_{438}/R_{437}R_{438}$).	This study
pJCTGCAN294	The 2.2-kbp *Sal*I-*Xba*I fragment from pJCTGCAN203 expressing Znc1p-GFP was replaced with the 2.2-kbp *Sal*I-*Xba*I fragment from pJCTGCAN277. (Znc1p $C_{22}C_{25}/R_{22}R_{25}$-GFP).	This study
pJCTGCAN297	The 2.2-kbp *Sal*I-*Xba*I fragment from pJCTGCAN203 expressing Znc1p-GFP was replaced with the 2.2-kbp *Sal*I-*Xba*I fragment from pJCTGCAN282. (Znc1p $L_{437}L_{438}/R_{437}R_{438}$-GFP).	This study

[a]C. Gaillardin. UFR Microbiologie et Génétique Moléculaire, AgroParisTech, Centre de Biotechnologie Agro-Industrielle, Thiverval-Grignon, France.
